# Dipoid-Specific Genome Stability Genes of *S. cerevisiae*: Genomic Screen Reveals Haploidization as an Escape from Persisting DNA Rearrangement Stress

**DOI:** 10.1371/journal.pone.0021124

**Published:** 2011-06-17

**Authors:** Malgorzata Alabrudzinska, Marek Skoneczny, Adrianna Skoneczna

**Affiliations:** 1 Laboratory of Mutagenesis and DNA Repair, Institute of Biochemistry and Biophysics, Polish Academy of Science, Warsaw, Poland; 2 Department of Genetics, Institute of Biochemistry and Biophysics, Polish Academy of Science, Warsaw, Poland; National Cancer Institute, United States of America

## Abstract

Maintaining a stable genome is one of the most important tasks of every living cell and the mechanisms ensuring it are similar in all of them. The events leading to changes in DNA sequence (mutations) in diploid cells occur one to two orders of magnitude more frequently than in haploid cells. The majority of those events lead to loss of heterozygosity at the mutagenesis marker, thus diploid-specific genome stability mechanisms can be anticipated. In a new global screen for spontaneous loss of function at heterozygous forward mutagenesis marker locus, employing three different mutagenesis markers, we selected genes whose deletion causes genetic instability in diploid *Saccharomyces cerevisiae* cells. We have found numerous genes connected with DNA replication and repair, remodeling of chromatin, cell cycle control, stress response, and in particular the structural maintenance of chromosome complexes. We have also identified 59 uncharacterized or dubious ORFs, which show the genome instability phenotype when deleted. For one of the strongest mutators revealed in our screen, *ctf18Δ/ctf18Δ* the genome instability manifests as a tendency to lose the whole set of chromosomes. We postulate that this phenomenon might diminish the devastating effects of DNA rearrangements, thereby increasing the cell's chances of surviving stressful conditions. We believe that numerous new genes implicated in genome maintenance, together with newly discovered phenomenon of ploidy reduction, will help revealing novel molecular processes involved in the genome stability of diploid cells. They also provide the clues in the quest for new therapeutic targets to cure human genome instability-related diseases.

## Introduction

Living cells have developed various mechanisms to detect and repair DNA lesions, to minimize changes and preserve genomic integrity. A variety of biological processes are involved: DNA replication and repair, DNA damage signal transmission and detection, and the pathways coordinating DNA metabolism with progression of the cell cycle [1]. Almost all of these mechanisms are shared by all life forms, from simple unicellular prokaryotes to higher organisms including humans. On the other hand, malfunction of the machinery governing genome inheritance leads to destabilization of the genome and, in the case of human cells, can manifest itself in phenotypes such as aging or development of diseases, particularly cancer [2]. Thus, elucidation of the rules that govern genome maintenance and identification of all genes involved in this process is extremely important from the human perspective.

It is generally accepted that somatic mutations and rearrangements are important triggers of the onset of malignancy [3]. In mammalian cells the frequency of spontaneous mutagenesis measured at heterozygous loci is in the range from 1×10^−5^ to 2×10^−4^ depending on cell type, the marker used and the age of the organism [4]. Most of the events observed in those experiments were due to loss of heterozygosity (LOH) at the marker locus. The mutagenesis frequency at hemizygous loci in the same cell lines was 10 to 30 fold lower [5], [6].

Yeast *Saccharomyces cerevisiae* is a model organism often used in genome stability studies. For technical reasons, including greater simplicity of molecular genetics manipulations, haploid cells were employed in the vast majority of those studies, including those employing various whole-genomic approaches [7]–[10]. However, *S. cerevisiae* cells can be cultivated and studied as both haploids and diploids; it has been shown that there is a two orders of magnitude difference in the frequencies of spontaneous DNA changes at *CAN1* marker between a haploid genome and diploid *CAN1/can1Δ* heterozygous genome [11]. Notably, there was no difference in the level of point mutations leading to canavanine resistance, like frameshifts, transversions and transitions; the much higher number of spontaneous DNA changes in diploid cells was due to LOH through gene conversion, allelic crossover, and chromosome loss events, much like mammalian heterozygous markers [11], [12]. Although events leading to genome instability in haploid and diploid cells are essentially different, being mainly point mutations in haploid cells and mostly recombination events in diploid cells, they all provoke changes in the DNA sequence i.e. mutations. So it seems that the difference in the magnitudes and varieties of mutagenic events, between heterozygous diploid and haploid markers is true both for mammals and for simple unicellular eukaryotes [4], [11]–[13]. This implies essential distinctions in the mechanisms of maintenance of haploid and diploid genomes and justifies the use of yeast as a model for studying these mechanisms. Needless to say, gross chromosomal rearrangements do occur in haploid cells [14]–[16] and their rate can be measured [17]–[19]. The level of gross chromosomal rearrangements (GCR) demonstrated in haploid cells is in the range of 10^−9^ to 10^−10^ per cell, per generation [14], indicating that their rate in wild type haploid yeast cells is 10^5^ fold lower than in diploid cells [11], [12], and is even lower than the haploid point mutation frequency, which falls between 10^−7^ and 10^−9^ depending on the marker used or mutagenic event considered [11], [20]–[23].

Yet the frequency of mutagenic events in diploid cells exceeds all of these by as much as two orders of magnitude, implying the existence of a true distinction between haploid and diploid genomes in terms of genome stability, indicating additional threats against the latter, most likely brought about by extensive recombination. While the advantages and disadvantages of having two copies of the genetic material have been analyzed theoretically [24], [25], cellular functions and mechanisms dedicated to diploid genome maintenance until recently did not attract as much attention as they deserve. We can expect that there are still undiscovered genes responsible for maintaining genome stability specifically in diploids. This gave us the impetus to undertake an extensive examination of genome maintenance processes in diploid cells, using the unique *S. cerevisiae* collection of knock-out strains (YKO), created by the *Saccharomyces* Genome Deletion Project [26] coupled with microarray technology.

This approach is widely used to rank the sensitivity or resistance of deletion clones to various agents on the genomic scale [27], [28]. It is also used in genomic screens for synthetic lethality [29], [30]. In the present study we employed this approach to screen the diploid deletion collection for clones that have an increased level of **s**pontaneous **l**oss of function at a heterozygous forward **m**utagenesis marker locus (SLM). The yeast knock-out (YKO) collection of more than 5000 homozygous diploid deletion mutants (HD) and over 1100 heterozygous diploid strains from the essential gene collection (ESS) of *S. cerevisiae* cells together with barcode microarrays were used. Three independent mutagenesis screens were applied with three markers: the inherently heterozygous mating type locus located on chromosome III and two newly created heterozygous loci on chromosome V: *CAN1/can1Δ* and *URA3/ura3Δ*.

The accumulated data identify new genes responsible for maintaining genome integrity of diploid cells. Our screens revealed the genome instability phenotype caused by deletion of several uncharacterized or dubious ORFs. We have also found that numerous well characterized genes not previously associated with genome maintenance seem to be functionally linked to this process. The attributed function of many known genes selected in our screen suggests a mutator phenotype of the deletion, although it was never shown in a direct assay. The most interesting was the finding that the diploid strain missing both copies of *CTF18* gene, encoding a protein important for sister chromatid cohesion, has the ability to become haploid by losing an entire chromosome set from its genome. After the conversion of *ctf18Δ/ctf18Δ* diploid into a haploid, cells become genetically more stable and have higher chances for survival. Our data suggest the existence of an intriguing mechanism of escape from rearrangement catastrophe through the conversion to haploid. Since we found that several other deletion clones, besides *ctf18Δ*, exist as haploids within the homodiploid YKO collection, the observed phenomenon may also be triggered by other deficiencies that lead to diploid genome instability.

## Results

Our approach to this study was to make it as thorough as possible, by performing three independent whole-genomic screens with three mutagenesis markers. The inclusion in our screens of the diploid collection of clones lacking one copy of essential genes (ESS) allowed us to distinguish potential gene dosage effects on spontaneous loss of function mutagenesis (SLM) frequency, in addition to the lack-of-function phenotype detectable among homozygous diploid (HD) clones.

The mutagenesis markers used were the mating type locus located on chromosome III, and *CAN1* and *URA3*. The *URA3* and *CAN1* genes are both located on chromosome V; *CAN1* is located distally, whereas *URA3* is separated from the end of chromosome by a number of essential genes. It is highly probable that in the *CAN1* mutagenesis screen we could select deletion strains with an increased rate of chromosome arm loss that would be absent from the *URA3* mutagenesis screen. Hence, the existence of marker specific mutator strains in the analyzed population is to be expected. It is known from published data [31] that, at least for haploid cells, mutation spectra for *CAN1* and *URA3* markers are different. It has also been shown that in *rad5Δ* strains the frequency of UV-induced forward mutations at the *CAN1* locus is enhanced, but the reversion frequency of various ochre alleles is lowered [32]. To make screens for *CAN1* and *URA3* markers more reliable, control experiments were performed to detect genes whose deletion is sufficient to enable yeast cells with functional *CAN1* or *URA3* to grow in the presence of canavanine or 5′-fluoroorotic acid (5′-FOA) respectively. In addition, to include in the analysis slow-growing deletion clones, a comparison was performed to detect genes whose deletion extends the doubling time of yeast cells.

### Genomic screen for SLM at the *URA3/ura3Δ* and *CAN1/can1Δ* loci

The loss of functional *CAN1* gene (encoding arginine permease) makes the cell resistant to the toxic arginine analog canavanine, enabling use of this compound in tests for mutagenesis frequency. The lack of the functional *URA3* gene (encoding orotidine-5′-phosphate decarboxylase, which can convert 5′-fluoroorotic acid into toxic 5′-fluorouracil) makes the cell resistant to 5′-FOA. To employ these markers in our screens, we converted diploid HD and ESS clone collections into derivative pools containing heterozygous *URA3/ura3Δ and CAN1/can1Δ* marker loci. To create the derivative libraries we used a *can1::LEU2* cassette to perform simultaneous disruption of the entire clone pool, thus making the library heterozygous with respect to the *CAN1* gene (*CAN1/can1::LEU2*). To prepare the *URA3/ura3Δ* pool a linear DNA fragment carrying the *URA3* gene was used for transformation leading to restoration of wild-type *URA3* at one of the two *ura3Δ* loci. We optimized transformation conditions to preferentially convert only one copy of the target gene in each cell, to create heterozygous markers (see Supplementary Materials and Methods S1, supplementary Figures S1, S2).

We performed three independent SLM experiments with each of the *CAN1/can1::LEU2* and *URA3/ura3Δ* derivative pools. Cells were subject to selective growth for four days on synthetic complete (SC) plates supplemented with 30 µg/ml canavanine or 1 mg/ml 5′-FOA, respectively. Deletion clones displaying higher loss of function (LOF) rate acquired canavanine or 5′-FOA resistance more frequently, leading to higher than average representation in the population grown under selective pressure. Changes in relative abundance of individual deletion clones were evaluated by comparative hybridization of samples from cells of appropriate derivative pool grown in the presence and in the absence of selection conditions; this allowed identification of genes whose deletion causes an increase in SLM. In parallel, we performed control experiments to reveal the intrinsic resistance of some clones to canavanine or 5′-FOA. Such clones, if they exist, would be able to grow under selective conditions even with a functional mutagenesis marker gene. To test for canavanine resistance approximately 2.5×10^6^ cells of the original YKO diploid pool were subjected to selection on SC plates with canavanine. Similarly, 2.5×10^6^ cells of the derivative *URA3/ura3Δ* YKO pool were subject to selection on SC plates with 5′-FOA. In both resistance experiments, pools grown in the presence of canavanine or 5′-FOA were compared to those grown without selection, exactly as in SLM experiments. These experiments revealed that YKO clones resistant to either canavanine or 5′-FOA do indeed exist. A detailed analysis of this phenomenon is beyond the aim of this study, yet we did notice among the selected deletion clones overrepresentation of genes belonging to several distinct functional categories.

Another consideration was the defect in growth rate or cell viability that is quite often observed in the absence of genes involved in genome stability. Indeed, we did see higher variability in the colony size on the selection plates, where population was enriched with the mutator clones, than that seen on the control plates. To avoid distortion of our data by this variability, an additional control experiment was performed in which the relative abundance of every deletion strain in a newly inoculated YKO diploid pool culture was compared with its abundance in the same culture after approximately eight division cycles. The number of generations chosen was based on our estimation that mutant cells growing under selection underwent approximately eight doublings more than those from a control population grown without selection. By doing this comparison we could include in our selection the deletion clones that, due to the slow growth phenotype, are often overlooked in the genome-wide screens.

**Figure 1:**
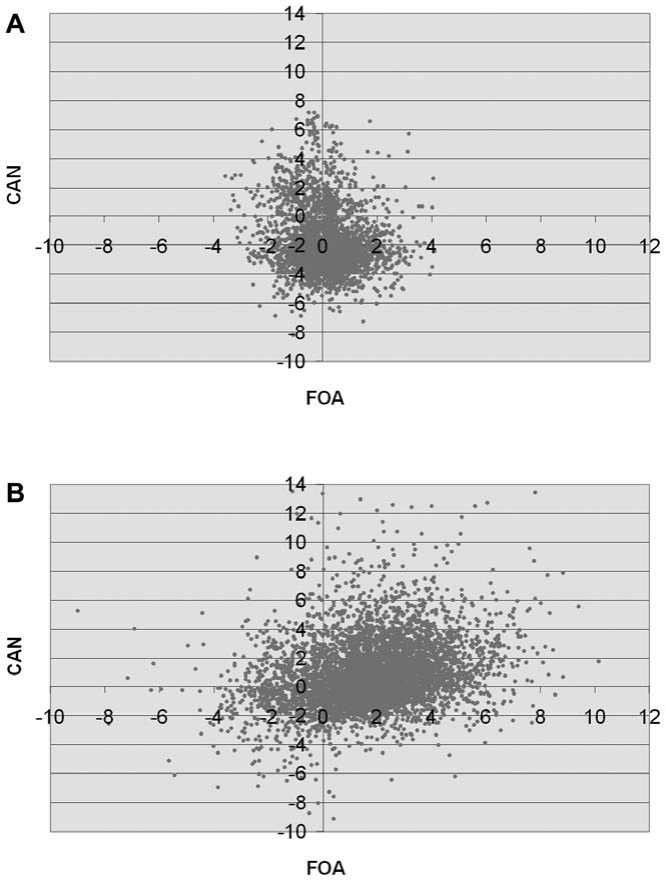
Comparison of genome-wide SLM screen results for *CAN1* and *URA3* markers. SLM screen results expressed as averaged LogRatio of relative abundance of each deletion clone obtained for *CAN1* and *URA3* markers were plotted against each other. LogRatio data derived only from the screens for mutator phenotypes show little correlation (A), whereas after subtracting the LogRatio data expressing resistance to the selection conditions and the LogRatio data expressing growth rate for each deletion strain (B) such a correlation exists.

For every gene the value of LogRatio expressing overrepresentation of deletion clone due to its resistance to canavanine and LogRatio expressing underrepresentation of deletion clone due to its slow growth were subtracted from the LogRatio defining the level of SLM for that clone obtained with *CAN1* marker. Likewise, LogRatios expressing resistance to 5′-FOA together with LogRatio expressing slow growth phenotype were subtracted from LogRatios defining the level of SLM with *URA3* marker. Figure 1 shows the comparison, in the form of a correlation plot, of LogRatios derived from *CAN1* SLM screen *vs* LogRatios derived from *URA3* screen, with (B) and without (A) subtracting the resistance and slow growth LogRatios. As can be seen, the inclusion of these controls increases the correlation between SLM results for canavanine and that for 5′-FOA. This post-processing of large scale data increased also the correlation between those data and the results of semi-quantitative spontaneous mutagenesis tests done on selected individual deletion clones (see below).

### Genomic screen for mutagenesis at the mating type locus

In this screen, the *MAT* locus from chromosome III was employed as a marker. Wild-type diploid cells are normally heterozygous at *MAT* locus and do not mate due to co-dominant suppression of haploid-specific cell differentiation pathways. The loss of either *MATa* or *MATα* locus restores the mating competence, and the mating type becomes that of the remaining allele. Mutagenic events in this assay are predominantly LOH due to recombination between homologous chromatids, gene conversion, chromosomal rearrangement or truncation, but can also be due to chromosome loss (diploid yeasts can be stably monosomic for chromosome III) [33], [34]. The rate of spontaneous loss of either of *MAT* alleles in wild-type cells is 2 to 4×10^−5^ [35]. In our genomic screen we crossed diploid YKO pool with sex tester strains, HB1-4Da or HB2-1Aα, and then identified by microarray the deletion strains that are either *MATa* or *MATα* maters at high rates (see Supplementary Figure S3).

The strains appearing in this screen would include also gene deletions leading to chromosome loss, which might not be seen in two other selections. From published data it is obvious that there is little or no loss of chromosome V, where the *URA3* and *CAN1* genes are located [33]. Among the selected deletion strains one can expect also to find those that display various perturbations in the sexual cycle. Diploids lacking both copies of such a gene may become mating competent and enter conjugation without any lesions in the mating locus.

The results of the three screens are summarized in supplementary Table S1. The final list contains genes that appeared in least two of the three SLM screens. The complete list of those 249 genes categorized by functional annotation is shown in Table 1. A more extensive description of these genes, including the results of all three screens and the description as appears in SGD (http://www.yeastgenome.org/) is shown in supplementary Table S2. The table includes also the data concerning the phenotypes of gene deletions or mutations that are relevant to genome maintenance. It should be emphasized that 105 out of 249 genes identified in our study have such phenotype annotations.

**Table 1:** 249 genes selected in SLM screens grouped on the basis of Biological Process functional annotation.

Biological process	Number of ORFs	Gene name
unknown	62	*AIM38, BRP1, DAN2, FMP46, KRE9, NAB6, PIH1, RBG1, RTS3, SCS22, SIP18, SKG3, TED1, UBP13, YAL065C, YAR047C, YBL096C, YBR032W, YBR116C, YBR197C, YBR300C, YBR124W, YDL062W, YDR193W, YDR209C, YDR290W, YDR370C, YER067C-A, YGR021W, YGR127W, YHL029C, YIL001W, YIL055C, YIL057C, YIL089W, YIL091C, YJL009W, YJL016W, YJR141W, YKL111C, YKR075C, YLR137W, YLR253W, YLR414C, YML079W, YML090W, YML131W, YMR111C, YMR185W, YMR194C-A, YMR206W, YMR279C, YNL046W, YNL086W, YNL140C, YNL143C, YNR065C, YOL079W, YOL087C, YOR139C, YOR304C-A, YPL238C*
genome integrity	42	cell cycle control: *BFA1, CDC16, HSL7, MAD1, NDD1, VHS1*cell division: *AKL1, BUD3, DDC1, DOM34, IML3, MCD1, LGE1, MPS3*chromatin maintenance: *ELF1, RLF2, RSC4, RSC9, SIF2, SWR1, VPS72*chromosome segregation: *BRN1, GIP3, SPC25, STS1*DNA replication and repair: *ABF2, CTF8, CTF18, DPB3, RAD1, RAD9, RFC5, KRE29, MPH1, MSH6, RAD24, RAD59*maintenance of genetic stability: *DUT1*sporulation: *YBR174C, YJR037W*telomere maintenance: *CGI121, PBP2*
metabolic processes	32	amino acid biosynthesis: *ARG4, CPA1, CPA2, ILV3, MEU1, THI80, TMT1*ceramide synthesis: *LAC1, LIP1*glycosylation: *ALG1, ALG14, GPI13, GTB1, OST5*dNTP biosynthetic pathway: *ADE3, ADE8, HIS1, RNR3*sterol biosynthesis: *CYB5, ERG10, ERG13*another metabolic processes: *ATP4, CAT2, DAL2, FUM1, HSD1, MAE1, MIS1, PDC1, UPS1, YAT1, YIL083C*
RNA metabolism	20	mRNA: *ABD1, CWC2, JSN1, SGN1, SKI3, YTH1*rRNA: *HAS1, IPI3, MPP10, RRP46, UTP13, YJL010C*tRNA: *MSM1, MTO1*RNA turnover: *SUV3*Diverse groups of RNA: *LSM4, POP8, PTI1, SLX9, YDR067C*
transport	18	Particles: *FUN26, KAP95, MUP1, NUP1, NUP57, PEX10, PEX22, TOM22, VPS51, YOL163W*Vesicular: *APS3, GEA1, GYP8, RAV1, SEC1, SEC15, SEC2, TRS120*
stress response	17	high Na+ alkaline pH or cell wall stress: *FRT2*osmoregulatory glycerol response: *SGD1*oxidative stress, response: *AHP1, ALO1, GPX2, OCA1, RIM15, YBR014C*response to drug: *AFG2, BLM10, PHM6, SSD1, TPS1*response to pheromone: *PRM9*response to starvation: *GCN2*unfolded proteins and HS response: *HSP26, SSA2*
transcription	15	*BRF1, CTI6, HDA3, HIR2, MKS1, MTF1, NUT2, RPA34, SSU72, STP2, TAF11, TOF2, WHI5, XBP1, YRR1*
translation	13	*GCD6, GCD11, MAK21, MRPL7, MRPL15, MRPL16, MRPL28, MRPL39, MRPS16, MRPS5, RPL4A, RPS22A, RSM24*
protein regulation	11	protein folding: *CCT4, CNS1, PAC10, PET100*protein modification: *PIB1, RUB1, RXT3, TUL1*protein degradation: *HLJ1, UBC1 UFD1*
mitochondrion maintenance	6	*CYC2, DNM1, MDM35, MDM36, PET191 YMC2*
cytoskeleton organization	5	*PAN1, PFY1, ROM2, SIW14, SLG1*
metal homeostasis	4	*LPE10, NBP35, YGL260W, YKE4*
cell wall organization	3	*CCW14, ECM33, PKH3*
microautophagy	1	*MEH1*

### Semi-quantitative drop assay of SLM for individual deletion clones

To validate our genome-wide LOF mutagenesis screen, it was important to confirm the mutator phenotype shown in the global approach by a mutagenesis frequency assay on individual deletion clones. These individual SLM tests were carried out on a sizable sample of deletion clones. To enable testing of a large number of strains, we developed a semi-quantitative drop assay of SLM (see Materials and Methods). All chosen strains needed a marker for LOF prepared before testing. We prepared 98 strains that are heterozygous at the mutagenesis marker; 83 of them were in the HD YKO collection and 15 were from ESS YKO library. We disrupted the *CAN1* locus with the *can1::LEU2* cassette in 51 diploid strains (including 6 ESS) and introduced one wild type *URA3* gene into 47 different strains (38 HD, 9 ESS) (see Supplementary Table S3). We performed our drop assay of SLM on at least 5 independent isolates of each analyzed strain.

**Figure 2:**
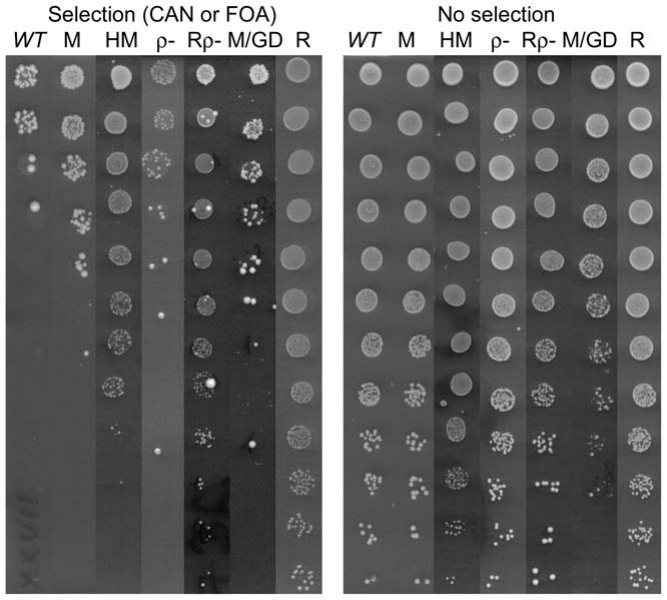
Example of results of the semi-quantitative SLM drop assay showing various categories of mutator phenotype. Cell suspensions were serially diluted and spotted onto selection plate (with canavanine or 5′-FOA) and onto dilution control plate as described in Materials and Methods. *WT* – SLM level in parental strain, M - increased SLM phenotype, HM - high SLM phenotype, ρ^−^ - increased SLM due to respiratory incompetence in *WT* ρ^−^ strain, Rρ^−^ – resistance to selection conditions acquired along with the loss of respiratory competence, M/GD - high SLM phenotype accompanied by decreased survival rate, seen also without selection, R - full resistance to selection conditions.

Data from such individual tests not only helped to confirm the mutator phenotypes of selected deletion clones or to reject false positives, but also revealed some details of the mechanisms by which yeast cells acquire the ability to grow on canavanine or 5′-FOA supplemented media. As shown in Figure 2, in addition to SLM occurring at various levels in most of the strains tested (lanes M, HM and M/GD), full resistance to the applied selection is also observed (lane R). For some particular deletions, the resistance to selection conditions was acquired as a result of losing respiratory competence (lane Rρ^−^); in the BY4743 background respiratory incompetence itself results in the increase of SLM (lane ρ^−^).

The results obtained for a significant sample of selected deletion clones in individual SLM tests revealed around 80% accuracy of high throughput screening for each of the *CAN1* and *URA3* markers (see Supplementary Table S3). Among the remaining 20%, which in individual tests showed a different phenotype than expected from microarray data, are strains which are either hypersensitive to applied selection or slow growers (see Supplementary Table S3). Thus, the inaccurate signal observed in microarray data is probably due to the extremely low representation of some deletion clones in the analyzed population.

### DNA content analysis of deletion strains with strong mutator phenotype

**Figure 3:**
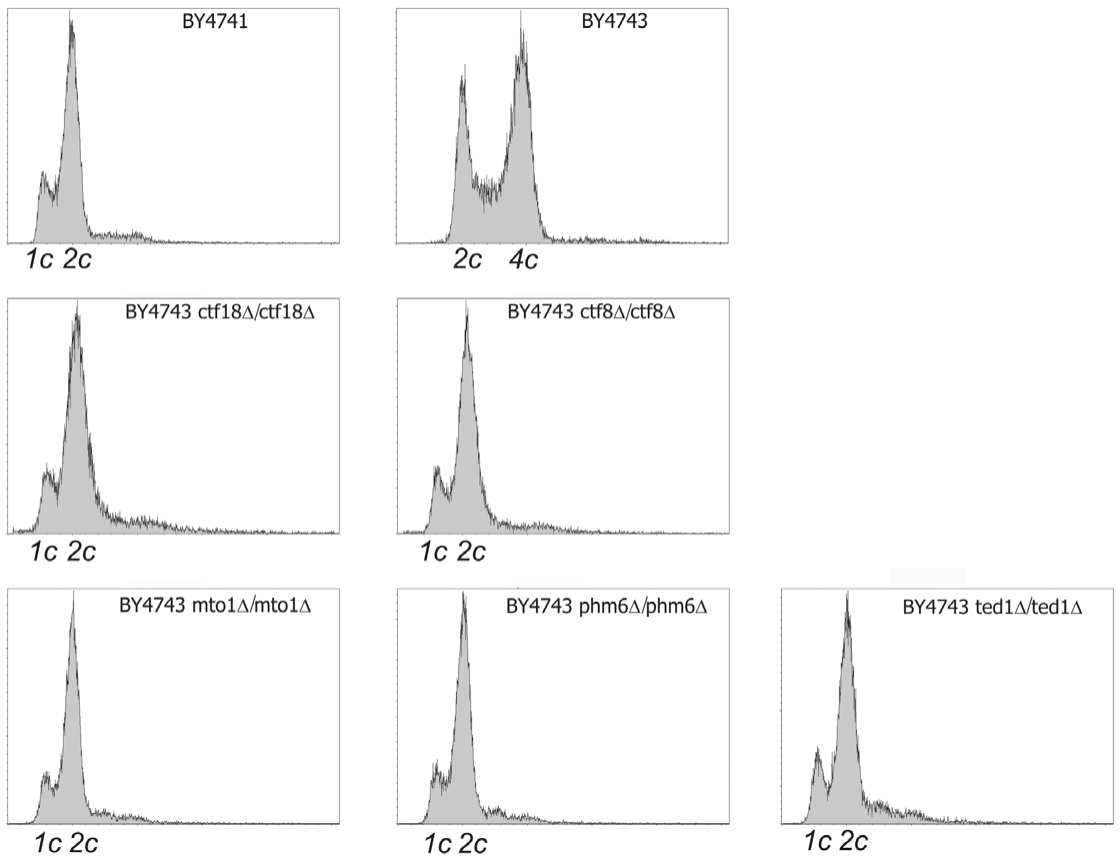
DNA content analysis of mutator strains in BY4743 background from homodiploid YKO collection. DNA content analysis of *ctf18Δ/ctf18Δ*, *ctf8Δ/ctf8Δ*, *mto1Δ/mto1Δ*, *phm6Δ/phm6Δ* and *ted1Δ/ted1Δ* strains in BY4743 background from homodiploid YKO collection. Wild-type BY4741 (1n) and BY4743 (2n) strains served as controls for DNA content. Propidium iodide stained cells were analyzed by FACS as described in Materials and Methods.

Chromosomal rearrangements may lead to abnormalities in DNA content within the cell. We have used fluorescence-activated cell sorting (FACS) analysis after propidium iodide staining to assess DNA content in cells of a number of individual homodiploid deletion clones that showed an overall strong mutator phenotype in our screens (see Supplementary Table S2). To our surprise five of them, carrying deletions of *CTF18*, *CTF8*, *MTO1*, *TED1* and *PHM6* genes had DNA content typical for haploid rather than diploid cells (see Figure 3). The simplest explanation would be the erroneous placement of a haploid deletion clone within the homodiploid collection by its creators. In that case when the *can1::LEU2* disruption cassette is introduced into a haploid strain it becomes canavanine resistant, mimicking a strong mutator phenotype with canavanine selection. Haploid strains would also be mating competent. Yet the *URA3/ura3Δ* locus was created by introducing a healthy copy of *URA3*, so a haploid strain would not show up in our FOA resistance screen. Still, the *ctf18Δ/ctf18Δ*, *mto1Δ/mto1Δ*, *ted1Δ/ted1Δ* and *phm6Δ/phm6Δ* strains from YKO collection, in BY4743 background, had also high scores of SLM at *URA3/ura3Δ* locus. This made us to believe that *ctf18Δ*, *ctf8Δ*, *mto1Δ*, *ted1Δ* and *phm6Δ* strains with unexpected DNA content did not appear in the homodiploid collection as a result of human error, but rather that the change in DNA content in those cells was a consequence of the lack of respective gene products.

To further investigate the phenotype of the absence of these genes we created new homozygous diploid *ctf18Δ/ctf18Δ*, *ctf8Δ/ctf8Δ*, *mto1Δ/mto1Δ*, *ted1Δ/ted1Δ* and *phm6Δ/phm6Δ* strains, by crossing freshly made haploid deletion constructs of both mating types. These strains allowed mutagenesis tests in diploid cells. As shown in Table 2 all strains displayed mutator phenotype with both canavanine and 5′-FOA selection, confirming the earlier findings. However, in case of the strains with *CTF8* and *CTF18* gene deletions this phenotype was much stronger than in case of the remaining three deletion strains.

**Table 2:** SLM levels in diploid cells lacking *CTF18*, *CTF8*, *MTO1*, *PHM6* and *TED1* gene products.

Strain	*CAN1* SLM (Can^R^/10^4^)	*URA3* SLM (FOA^R^/10^4^)	*CAN1* SLM relative to WT	*URA3* SLM relative to WT
2n	0.94	0.24	1.00	1.00
2n ctf18	18.43	7.29	19.58	30.51
2n ctf8	9.44	3.35	10.02	14.04
2n mto1	1.11	0.68	1.18	2.84
2n phm6	1.06	0.44	1.12	1.85
2n ted1	1.20	0.34	1.27	1.42

SLM levels in freshly prepared 2n ctf18, 2n ctf8, 2n mto1, 2n phm6 and 2n ted1 homodiploid deletion strains and 2n (WT) strain at two mutagenesis markers: *CAN1* and *URA3*. The numbers represent medians from eight cultures of the independently prepared constructs for each strain. SLM was measured using semi-quantitative drop assay as described in Materials and Methods.

We excluded the possibility that *ctf18Δ*, *ctf8Δ*, *mto1Δ*, *ted1Δ* and *phm6Δ* strains from homodiploid YKO collection became haploid due to increased sporulation frequency; no sporulation of these strains was observed in rich medium. Moreover, as shown in Table 3, all five deletion strains showed three to fifteen-fold lowered sporulation frequency compared to wild-type parental strain, in sporulation medium. This is most likely a result of defects caused by the lack of respective genes.

**Table 3:** Sporulation frequency in diploid cells lacking *CTF18*, *NDT80*, *CTF8*, *MTO1*, *PHM6* and *TED1* gene products.

Strain	Average number of tetrads (%)	SD	relative to WT
2n	10.05	1.53 (n = 20)	1.00
2n ctf18	0.64	0.63 (n = 20)	0.06
2n ctf8	2.38	0.33 (n = 8)	0.23
2n mto1	3.50	1.15 (n = 8)	0.34
2n phm6	2.19	0.28 (n = 8)	0.21
2n ted1	0.64	0.19 (n = 8)	0.06
2n ndt80	0.15	0.31 (n = 20)	0.01
2n ndt80 ctf18	0.09	0.23 (n = 20)	0.01

Sporulation frequency was determined in freshly prepared 2n ctf18, 2n ctf8, 2n mto1, 2n phm6, 2n ted1 2n ndt80 and 2n ndt80 ctf18 homodiploid deletion strains and 2n (WT) strain. The frequency is expressed as a percent of tetrads scored relative to all cells counted (see Materials and Methods for details). Average values and standard deviations (SD) were calculated from the data for 8 or 20 cultures of independently prepared constructs for each genotype.

### The consequences of the absence of *Ctf18* protein in diploid yeast cells

**Figure 4:**
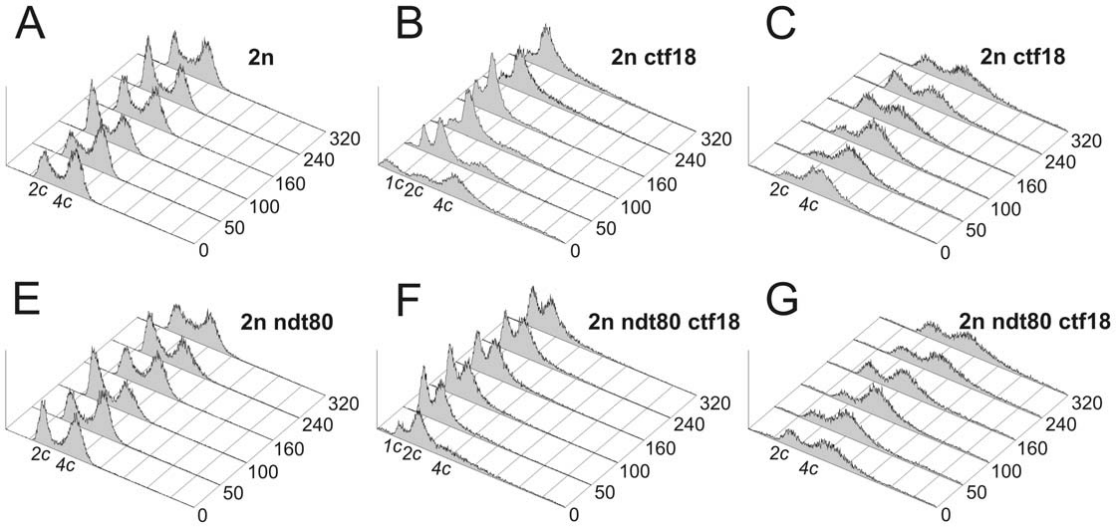
The changes of DNA content in cells of 2n, 2n ndt80, 2n ctf18 and 2n ndt80 ctf18 strains during prolonged growth. DNA content analysis was done after: 0, 50, 100, 160, 240 and 320 generations. Please note that “0” represents the starting point of the experiment. In fact, as we estimate, at this point the clones originating from the single zygotes had already grown for about 50 generations. Propidium iodide stained cells were analyzed by FACS as described in Materials and Methods.

Finally we explored striking possibility that the lack of a gene whose product is involved in genome stability might cause abnormalities in chromosome segregation resulting in the precise loss of one chromosome set, thereby converting diploid to haploid. For this test we used freshly made homodiploid strains of three genotypes: *ndt80Δ/ndt80Δ*, *ctf18Δ/ctf18Δ* and *ndt80Δ/ndt80Δ ctf18Δ/ctf18Δ*. Freshly made homodiploid strain with the wild-type copies of both genes was used as a reference. *NDT80* is the meiosis-specific transcription factor that is required for exit from pachytene [36], [37]. *ndt80Δ/ndt80Δ* diploids do not sporulate (see Table 3) so we added this deletion to our experiment design to diminish even further the likelihood that haploidization could occur as a result of sporulation. All strains contained also heterozygous mutagenesis marker loci *can1Δ/CAN1* and *ura3Δ/URA3*. Twenty independent diploid clones of each genotype were used in this experiment. Eight of twenty *ctf18Δ/ctf18Δ* clones that were used in prior pilot experiment were prepared by crossing eight *MATa* deletion clones with eight *MATα* deletion clones and purified by triple re-streaking on selective plates. All the remaining clones were isolated by catching zygotes after crossing freshly made haploid cells of both mating types bearing the appropriate deletions (see Supplementary Materials and Methods S1 for details). This latter method of strain preparation while being faster gave us full confidence that initially all clones were indeed diploid and were the progeny of a single cell. Their authenticity was further confirmed by testing their growth requirements. The resulting twenty homodiploids of each genotype were maintained for many generations on YPD plates at 28°C by transferring cells onto a fresh plate every 24 or 48 hours (depending on growth rate). We estimated that each such refreshing of the culture occurred after approximately 16 generations. After 50, 100, 160, 240 and 320 generations the DNA content within the propidium iodide stained cells was measured using FACS. Eighteen out of twenty *ctf18Δ/ctf18Δ* clones and eighteen out of twenty *ndt80Δ/ndt80Δ ctf18Δ/ctf18Δ* clones showed, with increasing generation number enhanced variation in DNA content of the cell population, manifesting as a broadening of the 4c peak with a shift in its maximum towards the right. Interestingly, for two out of twenty *ctf18Δ/ctf18Δ* clones and two out of twenty *ndt80Δ/ndt80Δ ctf18Δ/ctf18Δ* clones, a considerable fraction of cell population shows a DNA content characteristic for haploid cells after as little as 50 generations, and haploid cells dominate after further generations. On the other hand all wild-type and all *ndt80Δ/ndt80Δ* clones remained diploid throughout the experiment. Figure 4 shows, representative for each genotype, overlaid FACS profiles depicting DNA content changes with passing generations. For *ctf18Δ/ctf18Δ* and *ndt80Δ/ndt80Δ ctf18Δ/ctf18Δ* genotypes two profiles are shown for the clones in which haploidization occurred and for the clones that became aneuploid. Complete results for all clones of each genotype are shown in supplementary Figures S4, S5, S6, S7. Remarkably, for the clones that became haploid we do not see a gradual shift in DNA content to the left, rather there is a rapid appearance of haploid cells that were able to out-compete the rest of the population.

**Figure 5:**
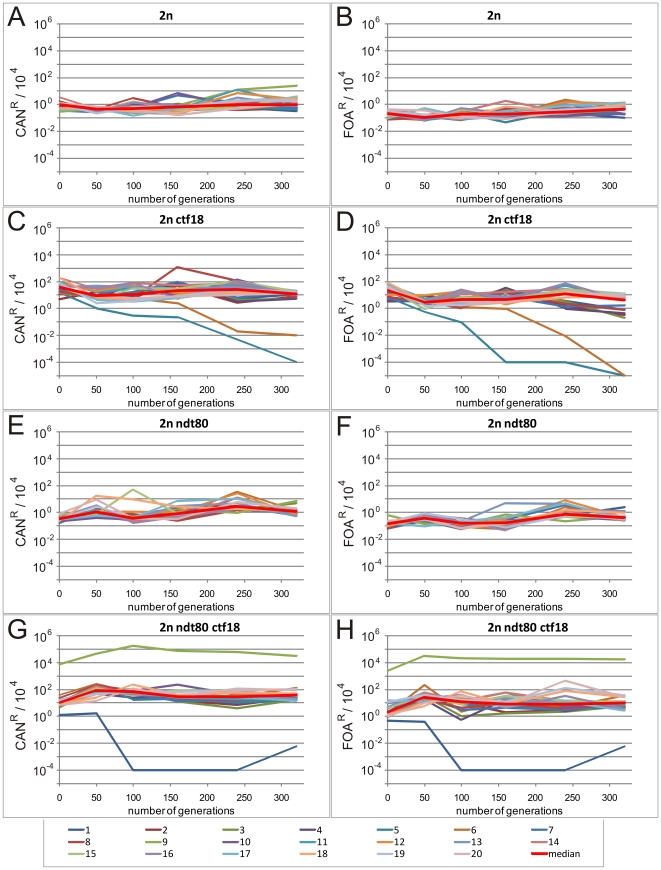
The changes of SLM levels in cells of 2n, 2n ndt80, 2n ctf18 and 2n ndt80 ctf18 strains during prolonged growth. SLM profiles for twenty independent clones of each genotype after growth for the indicated number of generations. SLM profiles for strains: 2n (A), 2n ctf18 (C), 2n ndt80 (E) and 2n ndt80 ctf18 (G) at *CAN1* locus. SLM profiles for strains: 2n (B), 2n ctf18 (D), 2n ndt80 (F) and 2n ndt80 ctf18 (H) at *URA3* locus. The plots for individual clones are marked with different colors; the plots of the median calculated from the data collected for twenty clones after particular number of generations are indicated by thicker red lines. SLM was measured using semi-quantitative drop assay as described in Materials and Methods.

In parallel, we tested the SLM at the *CAN1/can1Δ* and *URA3/ura3Δ* loci for all clones after 50, 100, 160, 240 and 320 generations. As seen in Figure 5, all wild-type and *ndt80Δ/ndt80Δ* clones and most of *ctf18Δ/ctf18Δ* and *ndt80Δ/ndt80Δ ctf18Δ/ctf18Δ* clones displayed stable level of SLM throughout the experiment, much higher for those with the deletion of *CTF18* gene. However two *ctf18Δ/ctf18Δ* clones and one *ndt80Δ/ndt80Δ ctf18Δ/ctf18Δ* clone that became haploid showed the decrease in mutation frequency. This is due to LOF mutagenesis in wild-type haploid *S. cerevisiae* cells being two orders of magnitude lower than in diploids. The second *ndt80Δ/ndt80Δ ctf18Δ/ctf18Δ* clone that converted to haploid became canavanine and 5′-FOA resistant apparently by losing the chromosome with wild-type *CAN1* and *URA3* genes. Remarkably, for all the clones that became haploid we noted an increase in average cell viability and shortened doubling time (data not shown).

We performed additional tests to study the nature of these presumably haploid cells. All the clones after 320 generations were crossed with haploid sex tester strains of both mating types. Only the clones that displayed the haploid DNA content were able to mate with either *MATa* or *MATα* tester strain.

On a subset of clones we tested also whether strains initially heterozygous at *URA3/ura3Δ* or *CAN1/can1::LEU2* preserved their heterozygosity after 240 generations, by PCR amplification of the respective genomic regions, using appropriate primers and examining the number and size of the resulting DNA fragments. Obtaining a doublet of PCR products of the sizes compatible with the sizes of wild-type genes and deletions would indicate that the heterozygosity was preserved. Such doublets were consistently amplified in all diploid and aneuploid clones, whereas two *ctf18Δ* clones that had haploid DNA content showed only single PCR products characteristic of wild-type *URA3* or *CAN1* alleles. Thus it appears that indeed those clones have lost heterozygosity at all three analyzed loci. Taken together with DNA content data, it is likely that those two *ctf18Δ/ctf18Δ* clones as well as two *ndt80Δ/ndt80Δ ctf18Δ/ctf18Δ* clones indeed underwent conversion to haploid.

**Figure 6:**
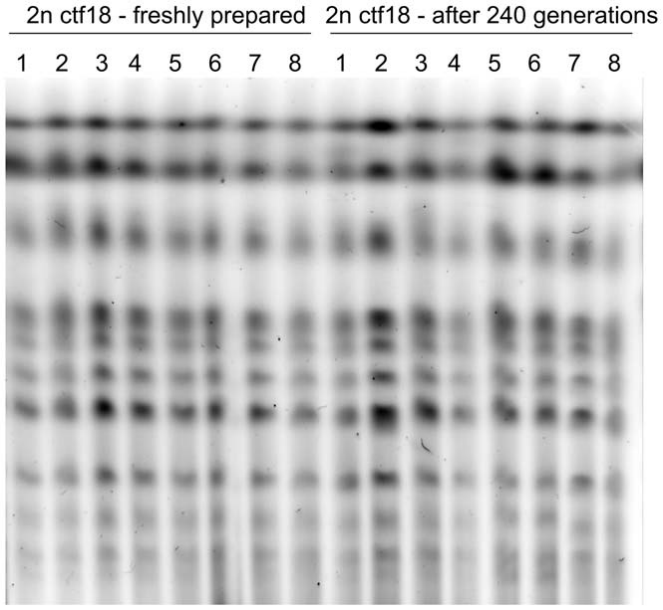
PFGE analysis of chromosomes from 2n ctf18 clones before and after prolonged growth. PFGE analysis of chromosomes isolated from eight freshly prepared 2n ctf18 clones (numbered 1 to 8) and from the same clones grown for 240 generations. See Materials and Methods for detailes.

To exclude the possibility that DNA content differences between the 2n ctf18 strains after 240 generations arose from severe chromosomal aberrations rather than ploidy reduction we analyzed the sizes of chromosomes of eight *ctf18Δ/ctf18Δ* clones before and after 240 generations by Pulsed-Field Gel Electrophoresis (PFGE). As shown on Figure 6 there are no visible differences in mobility and sharpness of chromosome bands between freshly made clones and those that underwent 240 generations irrespective of the DNA content.

## Discussion

### Chosing the strategy for identification of *S. cerevisiae* diploid deletion clones displaying the mutator phenotype

The collections of *Saccharomyces cerevisiae* strains with knockout of almost every gene present in the genome of this organism (YKO collections) constitute an invaluable and powerful tool enabling diverse functional tests on a genome-wide scale. Those tests can be done not only on individual strains but also on the mixed cell population containing all deletion clones in one culture, since each deletion strain is uniquely bar-coded with two 20 bp DNA sequences. The changes in relative abundance of individual clones in any mixture subjected to selection conditions can be monitored by PCR-amplification and labeling of the barcode sequences followed by comparative hybridization to barcode microarray [38], [39]. The collections have also proven to be a powerful tool for studying genetic interactions.

The screen for genes whose deletion results in genome instability holds one major difficulty. The strains deficient in such genes, being genetically unstable are less viable and, further, they will over time accumulate additional changes in their genomes. The strains that we intend to isolate, are at the same time the most difficult to preserve in their original state. Parental BY4743 contains two heterozygous markers *MET15/met15Δ* and *LYS2/lys2Δ* that could be conveniently used in LOF screen but in our experience heterozygosity of those loci is often lost, regardless of any defect in genome stability. Moreover, some of the potential mutators are slow growers and might be difficult to score as mutators in a high throughput screen. The barcode microarray-based SLM screen that we have devised establishes an improved method of detecting the mutator phenotype and provides the solution to these and other challenges. The key novelty of this method was the introduction of two new heterozygous markers *CAN1/can1Δ* and *URA3/ura3Δ* to the entire YKO collection. Equally important was the choice of the method of marker introduction. In theory the most reliable method of creating the collection of diploids homozygous for the deletion of every yeast gene and containing heterozygous LOF marker would be to introduce the marker into each clone of e.g. MATa deletion collection and then to cross each resulting clone with the respective clone from MATα deletion collection. There are, however, potential dangers that could compromise the quality of the clone set obtained in that way. Some deletion clones may mate inefficiently or not mate at all. One could reasonably expect that some of the clones defective in genome stability will fall into that category and thus will be excluded from the collection from the very beginning. Another obstacle would be the lack of methionine or lysine auxotrophy in some clones from the haploid collection making simple selection of diploids on drop-out medium impossible and necessitating the use of micromanipulator to catch diploid zygotes. Less laborious and less perfect would be to introduce the heterozygous marker into individual homozygous diploid deletion clones. With this approach, the inevitable failure of some difficult clones to transform successfully on the first attempt would require repeating, perhaps several times, the transformation procedure on a subset of the deletion strains. Thus the imperative to bring the derivative collection to perfection would increase time, labor and frustration. Moreover, any of these laborious approaches might turn out to be unproductive if we take into account that the strains we are most interested in are at the same time the least stable. Even collections prepared meticulously could soon become useless for genome instability selection. Thus we came to understand that the most streamlined approach would be the best and decided to introduce the LOF markers in a single transformation reaction done on the mixture of all deletion clones. With that approach it was achievable to prepare two separate derivative homodiploid clone mixtures with *CAN1/can1Δ* and *URA3/ura3Δ* markers, allowing whole-genomic estimates of SLM frequencies with more than one locus. Furthermore, we could set the starting point for DNA changes accumulation that was common for all deletion clones, and we could also narrow the time period between marker introduction and SLM assay to as little as 4 days, the equivalent of approximately 30 cell divisions. By optimizing the transformation procedure we could assure a single correctly targeted insertion of marker in as many as 99.9% of cells.

It is worth mentioning that a number of deletion strains clearly identified as mutators in our screens and selected for phenotype confirmation with the individual semi-quantitative test, later turned out to be extremely resistant to individual LOF marker introduction. So in retrospect we can say that in terms of deletion collection coverage and selection accuracy, the strategy chosen was at least as good as other, more laborious alternatives.

This method has of course its own shortcomings. We were aware that individual deletion strains might behave differently compared to the majority. Some may differ in transformation efficiency. Should it be lower than average, the clone would be underrepresented and the sensitivity of SLM detection for that clone will be lowered accordingly. Higher than average transformation efficiency does not cause any problems provided that marker cassette is still introduced in the right place and in single copy. By comparing the relative abundance of deletion clones before and after marker introduction using the same barcode microarray hybridization technique that was used for determination of SLM, we could assure that the derivative clone mixture containing the selection markers remained representative of the library. Another drawback of this method is the impossibility of performing any quality tests for correct marker insertion into the individual deletion clones. Although, on average, the great majority of Leu+ cells had a single copy of *CAN1* replaced by *can1Δ* and the great majority of Ura+ cells got a single copy of *ura3Δ* replaced by *URA3*, some individual clones may display different behavior as a result of specific gene deletion. Since marker insertion involves the mechanisms of homologous DNA recombination, deletion strains defective in aspects of genome stability might be among those with an improperly inserted marker. It seems, however, that any inaccuracies in marker insertion had minor influence on the results obtained with the derivative clone pool. If the LOF marker is inserted at some frequency in the incorrect locus then some cells would still have two wild-type copies of the *CAN1* gene and hence the frequency of SLM will be lowered. On the other hand, *URA3* inserted randomly but in single copy would likely form a functional marker as good as that when it is inserted in place of *ura3Δ*. Multiple nonhomologous insertions of *URA3* marker cassette would exclude that cell from the 5′-FOA resistance screen, whereas multiple nonhomologous insertions of *can1Δ* marker cassette would do no harm to the canavanine resistance screen as long as a single *CAN1* gene is replaced by *can1Δ* cassette. It should be borne in mind that our derivative clone pools would contain around fifty independent transformation clones of each original deletion strain. Even should some of them be faulty and do not participate in selection for canavanine or 5-FOA resistance, the remaining ones should still respond as expected. The only effect would be lowered sensitivity of mutator phenotype detection for that strain. If, for any given deletion strain, all transformation clones are incorrect then the relevant gene would be lost to our screen. Yet such problematic strains would likely be missing also from the derivative set composed of strains transformed individually.

To make this method effective as a screen for increased SLM, two important conditions have to be met. *Firstly*, the derivative pools heterodiploid with respect to mutagenesis markers must remain representative. To assure this, we prepared *CAN1/can1::LEU2* and *URA3/ura3Δ* heterodiploid pools with 58- and 42-fold coverage of yeast genome, respectively. The representativeness of both derivative pools was confirmed by comparison, using barcode microarrays, to the original HD+ESS pool. We observed that, despite our effort to assure the balance of the original pool (see Materials and Methods), less than 3% of all strains consistently gave a signal that was so low as to preclude them from the analyses. Among them could be the strains growing extremely slowly that despite of it were allocated to the homozygous diploid collection rather than to the essential heterodiploid collection. Also, the presence of faulty barcodes in some of the deletion clones resulting in low or no hybridization cannot be excluded [40]. Of the remaining over 97% deletion clones, only three were 15 to 10 fold underrepresented and another fifty were 10 to 5 fold underrepresented, relative to the parental pool. A further three hundred were 5 to 2 fold underrepresented. Thus, in our judgment the derivative pools remained sufficiently representative.

*Secondly*, the mixed population subject to canavanine or 5′-FOA selection should contain a sufficient number of cells of each deletion clone. Unlike in typical sensitivity or resistance screens where all tested cells carrying a given gene deletion behave similarly, only a small fraction of cells of each clone, determined by its mutator phenotype, would acquire a mutation at the marker gene locus (*CAN1* or *URA3*). Therefore, to make this screen representative, the average number of cells of each clone used in the assay should be several-fold greater than the inverse of mutation frequency of the wild-type strain. Our tests revealed that SLM frequency in BY474X genetic background is 8.2×10^−7^ for Can^R^ and 6×10^−7^ for 5′-FOA^R^ in haploid cells, and is approximately two orders of magnitude higher, namely 1.5×10^−4^ and 1.4×10^−5^, respectively, in diploid cells. This is in accordance with published data [11], [12], [41]. Thus, for the screen to be representative, the initial number of cells per single deletion clone should be at least 10^5^ and the total number of cells in the whole population should be at least 10^9^ (see Supplementary Figures S1 and S2).

### Contribution of our SLM screen data to the genome maintenance field

Much large-scale data pertaining to the genome maintenance in *S. cerevisiae* exists in literature, including screens for the mutator phenotype in haploid cells [7], [8], for increased LOH phenotype in diploid cells [41], or for genome instability genes relevant to cancer [10]. The results of numerous global screens of sensitivity to various genotoxic stress are also available [9], [42]. There is only modest overlap of our gene list with any of the published studies, but they are also quite dissimilar (see Supplementary Table S2). Although superficially one would expect that screens for related phenotypes should produce similar gene lists, it should be kept in mind that each screen approach is different. In practice dissimilarities of the gene lists contents should be anticipated regardless of which phenotype is assessed or which biological process is explored with genome-wide approaches. To us it is clear indication that, in the case of genome stability, the search for genes involved should continue and that diverse screening conditions may reveal distinct functions related to this biological process. Nonetheless for almost half of genes from our list data exist suggesting the involvement of their gene products in the genome stability (see Supplementary Table S2).

Although our approach involved diploid cells, it was not limited to LOH events. Rather than focusing on this phenomenon, already extensively studied in excellent work of Andersen et al. [41], we aimed at identifying genes whose deletion or insufficiency (for essential genes) causes increased frequency of any DNA changes that could be detected with the employed markers. Those would include, besides LOH, point mutations, small deletions, epigenetic changes, or poorly characterized events. Rather than assigning mechanistic functions for gene products known for their involvement in genome stability, we were interested in finding new functional interconnections linking genome stability to other cellular processes. To make our screens more far-reaching, thus encompassing new, potentially interesting, functional groups of genes, two of them were performed on exponentially growing cells where any deficiency in genome stability systems will be better exposed than in postdiauxic or stationary phase cells. Both screens were done on the complete YKO collections with newly introduced heterozygous mutagenesis markers, *CAN1/can1Δ* or *URA3/ura3Δ*. The inclusion of the heterodiploid collection of essential gene deletions allowed us to study gene dosage effects for those genes.

### Genes implicated in the genome stability

Several remarkable trends emerged from our SLM screen. Essential genes comprise approximately a quarter of all genes (65 out of 249) that stabilize the genome. This underlines importance to the cell of preservation of genomic integrity. The 249 genes could be allocated to separate groups: 190 (76.3%) are verified genes (even though only 40 have known genome stability associations), 36 (14.46%) are uncharacterized and 23 (9.24%) are considered dubious (see Supplementary Table S2).

### Nuclear and mitochondrial localization predominates among gene products selected in SLM screen

**Figure 7:**
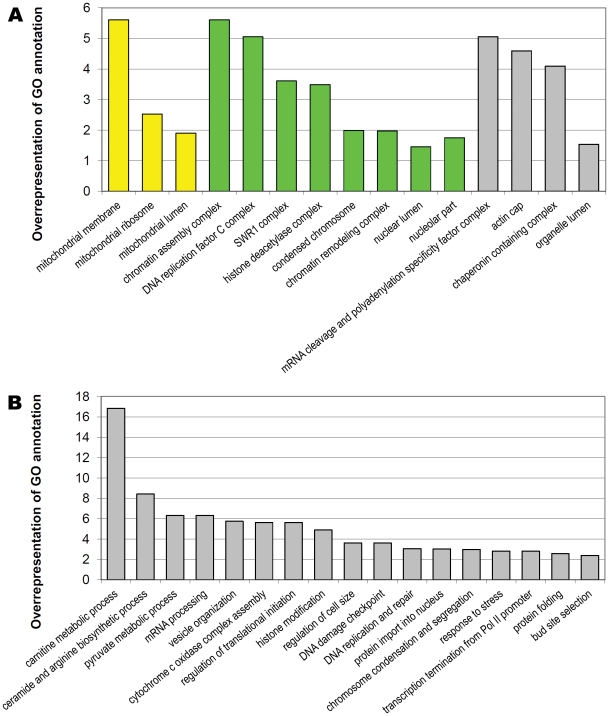
Overrepresentation of GO annotations in the group of 249 genes selected in genomic SLM screen. The analysis of overrepresentation of Gene Ontology annotations in the group of 249 genes selected in our large scale SLM screen was done with the help of GeneMerge on-line tool (http://genemerge.cbcb.umd.edu/); e<0.1. A) Overrepresentation of Cellular Component annotations. Annotations pertaining to nucleus are shown in green whereas those pertaining to mitochondria are shown in yellow. B) Overrepresentation of Biological Process annotations.

With respect to intracellular localization, the largest group of gene products can be found in the nucleus (32.12%, see Supplementary Table S2 and Supplementary Table S4, Figure 7A). Interestingly, a considerable fraction of these contains proteins located in the nucleolus (14 of 80 genes). This resembles the observation in *Caenorhabditis elegans* cells that links genome integrity and post-transcriptional RNA regulation functions via diverse RNA metabolic processes [43]. Although the presence of RNAi in *S. cerevisiae* cells has not been documented, several lines of evidence indicate the existence of posttranscriptional regulation in yeast cells. It is known that the loss of function of the exosome component Rrp6 leads to stabilization of *PHO84* antisense transcripts and subsequent inhibition of *PHO84* gene transcription. The data indicate that *PHO84* repression is not due to transcription interference, but results from antisense RNA-induced histone deacetylation by the Hda1/2/3 complex [44], [45]. In our screen we have found RNA degrading enzymes (*RRP46, SKI3*) and different components of histone deacetylating complexes (*HDA3, RTX3, SIF2*). Thus, we anticipate the existence in yeast cells of a posttranscriptional mechanism of gene expression modulation that influences genome stability in response of genotoxic stress.

Our data also confirmed the observation that abnormalities in ribosome biogenesis, which in turn lead to START delay and affect the cell cycle, can provoke genome instability [46]–[48]. In our screen we have found not only nucleolar genes responsible for rRNA processing and ribosome assembly (*IPI3, LSM4, MPP10, NOP9, POP8, PTI1, RRP46, SLX9, UTP13*), but also genes encoding: ribosomal subunits (*RPL4A, RPS22A, RSM24*, especially mitochondrial ones: *MRPL7, MRPL15, MRPL16, MRPL28, MRPL39, MRPS16, MRPS5*), proteins engaged in RNA transport (*HAS1, MAK21, NUP1*) and necessary for RNA turnover (*SUV3*), proteins involved in the synthesis of rRNA (*RSC9*) and rDNA silencing (*TOF2*) and, finally, START regulators, *WHI5* and *LGE1*, gene products whose role is tied to sensing the intracellular ribosome level (Table 1, Figure 7B).

Another considerable group of gene products is localized in the mitochondria. This can be explained in several ways, but most probably abnormal reactive oxygen species (ROS) production connected with deletion of a variety of mitochondrial genes results in an increase in endogenous premutagenic lesion formation [49]. An alternative explanation involves the essential role of mitochondria in the formation of iron-sulfur clusters, which perform catalytic and structural functions in many cellular proteins, among them DNA repair proteins, and as was recently shown, the maturation step of these proteins is required for the maintenance of nuclear genome integrity [50]. It is also possible that the imbalance in cytosolic dNTP pools due to mitochondrial dysfunction leads to chromosomal instability, as shown in human cells by Desler et al. [51]. In agreement with the last explanation is the observation that among deletion strains displaying genome instability is a group defective in dNTP biosynthetic pathways (*ADE3, ADE8, HIS1, RNR3*). Whatever the mechanism, the experimental data show that intact mitochondria are crucial for preservation of genomic integrity.

Many genes identified in the screen encode molecules located in vesicles, suggesting the participation of a vesicular path in the response to endogenous genotoxic stress. It is possible that response to stress requires the redistribution of protein(s) to an appropriate compartment. A number of genes whose products were connected with spindle pole body, bud neck, cytoskeleton and cellular wall were also found; these are likely to be engaged in proper cell division.

### Genome-wide SLM screen reveals genes whose products are involved in various mechanisms assuring genome stability as well as numerous genes unassigned to any biological process within the cell

The Gene Ontology (GO) annotations indicate that the most abundant group identified in our screen **has not been assigned** previously to any biological process (Table 1). This suggests that our knowledge concerning the maintenance of genome stability in diploid cells is rather incomplete and substantiates the motives that encouraged us to undertake this study. On the other hand, the known annotations of the remaining gene groups confirm the correctness of our experimental approach. Our data point to numerous molecular processes engaged in genome maintenance. As was expected, many genes encoding proteins engaged in DNA replication and repair (*ABF2, CGI121, DPB3, DUT1, KRE29, MPH1, MSH6, PBP2, RAD1, RAD5, RAD9, RAD24, RFC5*), cell cycle regulation (*BFA1, CDC16, HSL7, MAD1, NDD1, VHS1*) and cell division (*AKL1, BUD3, DDC1, DOM34, IML3, MCD1, LGE1, MPS3*) have been revealed. We have also identified a significant group of gene deletions that influence the chromatin state (*ELF1, RLF2, RSC4, RSC9, SIF2, SWR1, VPS72*), which in turn destabilizes genome integrity, because maintenance of chromatin assures chromosome stability.

Another interesting group of genes revealed by our screen are *DDC1*, *FRT2*, *MSH6*, *NUP1*, *RAD9*, *RAV1*, *SKG3*, *WHI5* and *XBP1*. These genes encode proteins that are either already documented or potential substrates for Cdc28p cyclin-dependent kinase, which, as recently shown by Enserink *et al.* [52], regulates proteins involved in DNA damage response and genome maintenance.

In addition, we have found a sizable group of genes whose products are involved in cellular stress responses (*FRT2, SGD1, AHP1, ALO1, GPX2, OCA1, RIM15, YBR014C, AFG2, BLM10, PHM6, SSD1, TPS1, PRM9, GCN2, HSP26, SSA2,*). Dysfunction in the stress response affects the ability of the cell to deal effectively with emerging problems that, as a natural consequence, manifests in genome destabilization.

### The genome-wide SLM screen reveals the components of ‘structural maintenance of chromosome’ (SMC) complexes

Among the gene products revealed by our genome-wide approach we found some that have especially drawn our attention. We found *MCD1*, *BRN1* and *KRE29* genes on microarray output list. These three essential genes encode subunits of three different complexes involved in assembling proper chromosome structure: cohesion complex, condensin complex and Smc5,6 complex, respectively. Two of these three ‘structural maintenance of chromosome’ (SMC) complexes directly regulate chromosome dynamics. The third, Smc5/6, functions mainly in homologous recombination and in completing DNA replication [53]. However, upon a double-strand break (DSB), cohesin complex is recruited to the DSB region through phosphorylation of H2AX and binding of another SMC complex, MRX (Mre11, Rad50, Xrs2) to the break site [54]. As can be expected, mutations affecting these complexes lead to chromosome aberrations. This phenotype has been shown mainly in meiotic cells, which demonstrate unequal division of genetic material, but for some mutations in SMC related genes, it has been also shown that they may cause aneuploidy in mitotic cells [55]. The fact that strains depleted in genes encoding essential subunits of different SMC complexes appeared in the screen for LOF mutator genes made us curious why other subunits engaged in building these complexes did not appear. Examination of the whole dataset revealed that some of the genes were missing because the strength of the deletion phenotypes caused the disappearance of the respective clones from the analyzed population. Others were present and displayed a mutator phenotype in high throughput screens, but at lower significance than the selected cut-off value. Comparison of the microarray data with the individual tests done on a small sample of clones that had a high mutator score in the microarray screen, but with too high a p-value, indeed revealed a quite good correlation. Hence, we decided to search all our microarray data, including those rejected because of a high p-value, for other components of SMC complexes. The results are presented in supplementary Figure S8. One can see the representation of all known SMC complexes, which regulate higher-order chromosome structure: cohesion complex (*MCD1, SMC1, SCC3*), condensin complex (*BRN1, SMC4, YCG1, YCS4*), Smc5,6 complex (*KRE29, NSE3, NSE5, SMC5*) and finally MRX complex (*XRS2, RAD50*) engaged in DSB repair. Further analysis revealed also other genes from SLM screen, encoding proteins responsible for physical interaction with cohesion Ctf4 protein, which binds also to Pol1 allowing it to access DNA (*CTF4, POL1*) and Ctf18-replication factor C (*CTF18, CTF8, RFC5*), which loads proliferating cell nuclear antigen (PCNA) on DNA. PCNA functions as a sliding clamp for replicative DNA polymerase and as a docking site for other proteins required for DNA replication and repair. We also noted the Rad24-replication factor C and its DNA binding partner from the 9-1-1 complex (*RAD24, RFC5, DDC1*), which form a platform enabling DNA polymerases to access the DNA template at the site of damage. We also observed *DPB3* encoding DNA polymerase-ε major subunit. Depletion of this gene is known already to have a mutator phenotype. These results show not only the involvement of SMC complexes in the maintenance of genome stability but, in addition, through their various interactions, suggest possible mechanisms of emergence of DNA alterations.

### Escape from rearrangement catastrophe through conversion to haploid

In light of these remarks the appearance of *CTF18* among the genes whose deletion shows the strongest mutator phenotype was not surprising. Unexpectedly, many of those deletion strains appeared as haploids residing within the homodiploid collection. If those arose as false positives due to strain misplacement their presence in our dataset would undermine the credibility of our results. However, we were able to prove that the lack of those genes in diploid yeast cells does result in the mutator phenotype. We also showed that the mutator phenotype of the deletion of *CTF18* is manifested by the conversion of diploid strain into a haploid. Thus it is likely that the absence in diploid yeast cell of genes such as *CTF8*, *TED1*, *MTO1* and *PHM6* (and possibly as yet undiscovered genes), leads to diploid to haploid conversion by the same unknown mechanism. Now the most important question is what **is** that mechanism?

The mutator phenotype arising from the absence of *MTO1*, *TED1* and *PHM6* genes, and the existence of respective deletion strains as haploid in homodiploid collection indicates the excessive incidence of genomic DNA abnormalities when those genes are missing. Remarkably, they have not been previously linked to genome maintenance processes.

*TED1* gene encodes a phosphoesterase domain-containing protein that acts in endoplasmic reticulum to Golgi vesicle-mediated transport [56]. It is one of many genes engaged in vesicular trafficking that appeared in our screens and we discussed this matter earlier.

Phm6 is a protein of unknown function, whose expression is regulated by phosphate levels. While the link between phosphate homeostasis and genome stability is unclear at the moment it was shown that several phosphate regulated proteins, like Pho80, Pho85 and Pho4, together with Rad9, Rad53 and Cdc28, are employed in activation of checkpoint response on DNA damage in G1 phase of the cell cycle [57]. In one of the early transcriptome studies *PHM6*, together with *CTF19* encoding the component of the kinetochore, were listed as responding to PHO regulatory pathway and possessing Pho4 binding sites on their promoters [58]. It might also be that phosphate metabolism influences the levels of intracellular nucleotide triphosphate pools [59] or that there is an interconnection between phosphate levels and the synthesis of pyridoxal 5′-phosphate (PLP). The results of a recent genome-wide study showed clearly that PLP levels are crucial for GCR suppression by curtailing the appearance of DNA lesions during the cell cycle [7]. In any case our data support the hypothesis that there is a functional link between the metabolism of this crucial nutrient and the genome stability.

Mto1 is a mitochondrial protein. It forms a heterodimer complex with Mss1 that performs the 5-carboxymethylaminomethyl modification of the wobble uridine base of mitochondrial tRNAs [60]. In *mto1Δ* strain the levels of many classes of mitochondrial tRNA are significantly lowered. The critical role of Mto1 in modifications at U34 of tRNA-Lys, tRNA-Glu, and tRNA-Gln, in mitochondrial 21S and 25S rRNA stability, in translation of *COX1*, *COX2*, *COX3*, *ATP6*, *ATP9* and *CYTB* mRNAs, in the maintenance of mitochondrial genome, and subsequently in respiratory competence, has recently been demonstrated [61]. The chain of events starting with wobbling tRNA deficiency causing the absence of crucial mitochondrial proteins ultimately results in the loss of mitochondrial DNA. This, as we discussed earlier, would compromise the stability of the nuclear genome. Even though the increase of SLM in freshly made diploid *mto1Δ* strain is modest (see Table 2), it increases with time (data not shown). Moreover, we saw a decrease in the sporulation frequency (see Table 3) and we noticed the increased frequency of petite colonies during the construction of *mto1Δ* strains (data not shown). So it is conceivable that this phenotype, relatively weak soon after the deletion of the gene, may grow stronger leading to chromosomal rearrangements and haploidization after sufficient number of generations.

Haploid *ctf18Δ* was previously shown to lose individual chromosomes easily [35], [62]. It has also been shown that *CTF18* deficient strain is unable to grow as a tetraploid at restrictive temperature so it was referred to as ploidy-specific lethal mutation [63]. Recently it has been shown that Ctf18 interacts physically with DNA polymerase ε, origin recognition complex, Cdt1 and minichromosome maintenance proteins, which suggests important role of Ctf18 in regulating the initiation of DNA replication [64], [65]. *CTF18* encodes a major subunit of the Ctf18-replication factor C (see supplementary Figure S8) that loads PCNA sliding clamp on DNA, interacts with cohesion complex and is involved in chromosome segregation during cell division [66], [67]. Thus, the absence of Ctf18p will likely cause severe chromosomal aberrations [55], [68]. Yet to our knowledge, the phenomenon of losing an entire chromosome set from a diploid cell as a consequence of lack of *CTF18*, or any other gene, was never reported.

The phenomenon of ploidy loss was in fact reported but for tetraploid strains of *C.albicans* [69]. Recently, it has also been shown that after several hundred generations, ploidy reduction towards diploidy occurs also in both triploid and tetraploid lines of *S. cerevisiae* [70], [71]. The data presented in those papers suggest that the chromosome loss was not random but rather that full sets of chromosomes were lost at once. These results imply the existence of a mitotic mechanism allowing the elimination of an entire set of chromosomes in yeast, thereby reducing the ploidy level. Interestingly, polyploidy reduction observed in those studies always led to diploid cells. On the other hand, it has been shown that after sufficient number of generations haploid strains can also convert into diploids. In that case the conversion process requires more time, occurring after about 1800 generations [71]. The results of those studies clearly show that the diploid state is a favorable one for standard laboratory *S. cerevisiae* strain maintained in typical conditions.

The phenomenon that we have found for *ctf18Δ/ctf18Δ* strain is quite different. One can notice two alternative routes that differ in cell destiny: either the cells reduce the ploidy of their genome to the 1c level, which seems to be stable, or GCR in the genome will continue resulting in a very heterogeneous population of cells varying in their level of polyploidy or aneuploidy as well as in their viability. Remarkably, these changes are accompanied by an additional phenotype regarding SLM. When the cells continue to accumulate the rearrangements their average genome size increases and SLM remains high. Whereas, when the cells manage to reduce the ploidy of their genetic material (thus minimizing the possibility of rearrangements), SLM is diminished thereby increasing their chances for survival (see Figure 4 and 5). This is documented by the domination of the population by haploid cells in two clones of *ctf18Δ/ctf18Δ* genotype and two clones of *ndt80Δ/ndt80Δ ctf18Δ/ctf18Δ* genotype. It is further substantiated by our observation that the cells in those cultures had on average shorter doubling time and higher survival rate than the cells from the remaining cultures.

Therefore we postulate that the reduction in ploidy from 2c to 1c by the cells devoid of functional Ctf18 is not accidental but rather is a new mechanism of avoiding the severe condition of genomic instability. We envision this phenomenon of conversion into haploid as a route to escape from rearrangement catastrophe. The mechanism governing this process remains to be explained, but our data clearly indicate that it is triggered by the deficiency of Ctf18 protein. Several conjectures can be made about this phenomenon on the basis of our current knowledge. *S. cerevisiae* can grow vegetatively both as haploids and diploids. The fact that the rate of GCR events in diploids is so much higher than in haploids suggests that under the risk of severe DNA damage discarding of the extra genome may act in favor of the genome preservation and sufficiently outweigh the disadvantage of short term lack of genetic heterogeneity and other benefits of diploidy. This can easily be reestablished by conjugation once the stress conditions disappear. Building up of GCR during prolonged exposure to environmental stress would lead to so extensive rearrangements and aneuploidy, such that the disposal of precisely one chromosome set would be impossible. Therefore successful escape from rearrangement catastrophe should be undertaken soon after the conditions that triggered it as suggested by our results. Since haploid cells dominate the *ctf18Δ/ctf18Δ* population after as little as 50 to 100 generations they must have appeared quite early.

Two possibilities present themselves. This phenomenon might occur purely by chance, starting with an early sporadic event of losing an exact chromosome set as a direct result of the absence of *CTF18* gene. Alternatively, it may be an adaptive mechanism, encoded by some other genes, that increases the likelihood of survival of a cell subject to severe DNA abnormalities caused by the absence of *CTF18* gene. Ctf18 is engaged in double-strand break repair by homologous recombination [72], a biological process involving mitotic sister chromatid cohesion [73]. Absence of this protein leads to extensive aneuploidy clearly documented by our DNA content analysis. It is difficult to imagine how the diploid cell devoid of Ctf18 could lose whole chromosome set at once accidentally. A more likely possibility would be the gradual decrease of DNA content in such cells, but this is not what we see; there is either rapid conversion to haploid or gradual randomization of the DNA content drifting to values higher than 2n. While at first it seems difficult to accept that the mechanism of escape from rearrangement catastrophe through haploidization is adaptive, to us it is not unlikely and moreover, it sounds very appealing, especially considering that haploidization occurred by exactly the same means in separate cultures of clones lacking Ctf18. The ultimate mechanism must be based on experimental evidence; if one assumes that haploidization is adaptive, then it must have evolved in response to natural DNA abnormalities. What kind of naturally occurring stress resulting in conversion of diploid into haploid is imitated by *CTF18* and possibly also by *MTO1*, *TED1* and *PHM6* gene deletions? Is this phenomenon unique to diploid *S. cerevisiae* cells lacking Ctf18 protein or it is more general strategy of survival of diploid microorganisms in a hostile environment? These are important questions that should be resolved experimentally in a separate study.

Despite the distinctive phenotypes of their deletions *MTO1*, *TED1* and *PHM6*, identified with our approach, did not show up among the genes selected in two other genome-wide screens aiming at similar phenotypes, both employing crosses with diploid YKO collection strains: searching for diploid bimater strains [10] and looking for gene deletions that restore mating competence to diploid strains [74]. Only *ctf8Δ/ctf8Δ* and *ctf18Δ/ctf18Δ* from our list of haploid strains in diploid YKO collection were reported in those studies. It is therefore possible that the list of genes whose deletion results in 2c to 1c conversion is incomplete. Further genome-wide screens designed specifically for selection of haploids within homodiploid collection may reveal more genes with a role in genome stability, whose deletion results in a specific ploidy reduction. In addition, they will help to determine the overall quality of the homodiploid *S. cerevisiae* knock-out collection. Such experiment would certainly be useful for anyone using the collections. Regardless of the results of those screens, the performance of diploid *ctf18Δ/ctf18Δ* and other deletion strains of similar phenotype strongly suggest the need for redefining the ‘essential’ gene attribute. For practical reasons this category should also encompass the genes like *CTF18*. After several generations, strains carrying such a gene deletion accumulate so many secondary changes in its genome they are no longer the same strain. Effectively, the deletion of such genes does not permit the strain to exist in its original state, so in a sense that gene could be called ‘essential’. Alternatively, separate category could be established e.g. ‘genetically unstable’ to emphasize the characteristic of those deletion strains.

### Conclusions

In summary, the genome-wide SLM screen that we have designed is a powerful tool for investigating genome stability. We were able to find genes responsible for maintaining genome integrity of diploid cells. Our screen revealed a genetic instability phenotype of 59 strains associated with the deletions of uncharacterized or dubious ORFs. This implies the existence of new molecular functions and possibly new processes involved in genome maintenance. We have also found functional associations with genome integrity of many well characterized genes that were not previously linked to this process; the suggested mutator phenotype of the deletion had never been shown in a direct assay. Moreover we showed that the lack of some genes made the diploid yeast cells to display an exceptional phenotype, a tendency of conversion to haploid. We believe that our results revealed novel mechanism involved in the genome stability that helps the cell to survive the rearrangement catastrophe.

## Materials and Methods

### Strains and plasmids construction

*S. cerevisiae* gene knock-out collections version 2, created by *Saccharomyces* Genome Deletion Project (http://www-sequence.stanford.edu/group/yeast_deletion_project/) were obtained from Open Biosystems (Huntsville, USA) as deep-frozen glycerol stocks in 96 well microtiter plates. Detailed description of yeast strains and plasmids and growth conditions used in the study is given in Supplementary Materials and Methods S1.

### SLM assay for YKO *CAN1/can1::LEU2* or *URA3/ura3Δ* heterodiploid pools

10^9^ cells of *CAN1/can1::LEU2* or *URA3/ura3Δ* heterodiploid YKO pool (see Supplementary Materials and Methods S1 for a detailed description of pool preparation) were inoculated into 1 liter of liquid YPD-GPS medium and grown at 28°C with shaking at ∼200 rpm until they reached the density of 1–2×10^7^ cells per ml. The cells were collected, washed with, and resuspended in sterile 0.9% NaCl to a final density of 4×10^8^ cells per ml. Suspensions of *CAN1/can1::LEU2* or *URA3/ura3Δ* pools were plated (2×10^8^ cells per plate) on 150 mm SC-arginine plates supplemented with 30 µg per ml of canavanine, or on SC+uracil supplemented with 1 mg per ml 5′-FOA, respectively. At least 20 plates per pool were plated. In addition, the respective pools were plated, at about 2.5×10^6^ cells each, on canavanine or 5′-FOA plates to generate resistance control samples, and on SC plates to generate control without selection samples. The number of cells used gave over 400-fold coverage of the deletion collection. Various dilutions of each pool were also plated on media with and without selection to monitor the mutation frequency. All plates were incubated at 28°C for 4 days.

Colonies were washed from each plate with ∼8 ml of liquid YPD per plate with the aid of glass spreader. The combined cell suspension was mixed well and two aliquots containing 10^9^ cells each were taken for genomic DNA preparation. Cells were centrifuged and the pellets were flash frozen in liquid nitrogen and kept at −70°C until preparation of genomic DNA.

To generate the growth rate control samples, 10^9^ cells of YKO diploid pool were inoculated into 1 liter of liquid SC medium and cells were grown for about 15–16 h until they reached a density ∼2.5×10^8^ cells per ml, which is equivalent to eight generations. Two aliquots containing 10^9^ cells were harvested for preparation of genomic DNA.

### SLM assay at mating-type locus

10^9^ cells of YKO diploid pool were inoculated into 1 liter of liquid YPD-GPS medium. The sex tester strains HB1-4Da and HB2-1Aα were inoculated into 400 ml of liquid YPD. Cells were grown at 28°C with shaking at ∼200 rpm until they reached density of 1–2×10^7^ cells per ml. Cells were harvested and resuspended in liquid YPD to final density of 1×10^8^ cells per ml.

Suspensions of YKO diploid pool were mixed with either HB1-4Da or HB2-1Aα at a ratio of 1∶1 (10 ml of each) and left for 4 h at room temperature. The cells were then washed, resuspended in 10 ml of 0.9% NaCl solution and plated on twenty 150 mm YNB+2% glucose plates (∼2×10^8^ cells per plate) for each mating sample. YKO diploid cell pools were also plated onto 5 SC plates (5×10^6^ cells per plate) to generate the control sample. All plates were incubated at 28°C for 3 days. Colonies were washed off each plate series with ∼8 ml of liquid YPD per plate with the aid of glass spreader. The combined cell suspension was mixed well and two aliquots containing 10^9^ cells each were taken for genomic DNA preparation, as described above.

### DNA labeling and hybridization to Agilent Barcode Arrays

Genomic DNA was isolated from culture samples of 10^9^ cells after which the barcodes were labeled by PCR and hybridized to barcode microarrays (Agilent, Santa Clara, USA). Scanning and feature extraction was done using Axon GenePix 4000B scanner and GenePix Pro software (Molecular Devices, USA). See Supplementary Materials and Methods S1 for the detailed protocols of DNA isolation, labeling and hybridization.

### Data analysis

Statistical analysis of data was done using Acuity 4.0 (Molecular Devices, USA). Some manipulations were also done in Excel. When creating the YKO collection, the deletion of the majority of genes was accompanied with insertion of two barcodes, UPTAG and DOWNTAG, flanking the *kanMX* marker. However, 193 deletion clones were made with only UPTAG barcode. Also, we observed that for some deletions with both barcodes present, one of the barcodes was working and giving a reliable fluorescence signal, whereas the other one consistently did not. For these reasons, to avoid getting false negative data, we did not average the LogRatios for UPTAGs and DOWNTAGs, but instead, selected the results for the barcode that passed the statistic reliability criteria and gave higher LogRatio value.

### Data deposition

Microarray data are MIAME compliant and available at ArrayExpress (http://www.ebi.ac.uk/arrayexpress/), accession number E-MEXP-2685.

### Individual tests: Semi-quantitative drop assay of SLM

To estimate LOF level in the individual tests we have developed semi quantitative drop-assay for mutagenesis. In each deletion strain selected for individual test heterozygous *CAN1/can1::LEU2* or *URA3/ura3Δ* marker was created by transformation with linear DNA fragment containing the appropriate construct. All resulting strains were then grown in YPD medium with shaking at 28°C to a density of 1–2×10^7^ cells per ml. 5×10^7^ cells of each assayed clone were centrifuged, washed with 0.9% NaCl, resuspended in 150 µl of the same solution and placed into each of eight leftmost wells of microtiter plate (96 well format). This allowed to test eight clones on a single plate. Eleven 3-fold serial dilutions in 0.9% NaCl solution were made, using 8-channel pipette. 3.3 µl of each diluted cell suspension was spotted onto Omnitray (Nunc) plates containing selection medium (SC-arginine+canavanine or +5′-FOA) or SC medium for dilution control. Plates were incubated at 28°C for 2–3 days. Colonies grown on both types of plates on countable spots were totaled and mutation frequencies were calculated, taking the dilution factor into account. At least 5 independent *CAN1/can1::LEU2* or *URA3/ura3Δ* heterodiploid clones of each analyzed deletion strain were assayed, and the median was calculated to obtain the final SLM estimation.

### Quantitative tests for mutagenesis

Test for spontaneous mutagenesis was made as in [75] with some changes. Briefly, yeast cultures were grown in liquid SC medium at 24°C to mid-logarithmic phase (1–3×10^7^ cells per ml) and plated (in duplicate) on appropriate selective plates (SC+canavanine or +5′-FOA). Plates were incubated at 28°C for 4–5 days before the number of mutant colonies was counted. To calculate the frequency of mutations, the number of mutant colonies was normalized to the number of colonies grown on SC medium with no selection. In each experiment 6–10 independent cultures of the tested yeast strain were analyzed. The presented data are the mean value from at least three separate experiments.

### FACS analysis

DNA content in yeast cells was measured by fluorescence-activated cell sorting (FACS) as described by Asami *et al.* [76]. A 1 ml aliquot of cell suspension was sampled from the culture broth at 0.2–0.5 OD600. After removal of culture medium by centrifugation for 1 min, the cells were resuspended in 1 ml 70% EtOH solution chilled at −20°C. The resultant suspension was kept at least 2 hours at 4°C to ensure complete fixation of cells. Fixed cells were washed twice in FACS buffer (0.2 M Tris-HCl pH 7.4, 20 mM EDTA) and resuspended in FACS buffer supplemented with RNase A (to final concentration 1 mg/ml) followed by 2 hour digestion in 37°C. The cells were washed with PBS and stained with 100 µl propidium iodide (50 µg/ml diluted in PBS) overnight at 4° in the dark. After adding of 900 µl PBS the cells were vortexed vigorously, flow cytometric DNA content analysis was performed using Calibur FACS analyzer (Becton-Dickinson, USA).

### Sporulation frequency assay

Diploid strains were incubated in sporulation medium (0.1% Yeast extract, 1% potassium acetate, 0.05% glucose) for 7 days at 30°C with shaking. The frequency of sporulation was then determined by counting tetrads in a cell counting chamber and expressed as percent of tetrads relative to all cells counted. Average and standard deviation (SD) was calculated from the data for eight or twenty cultures of independently prepared constructs for each strain.

### Pulsed-Field Gel Electrophoresis of yeast chromosomes

The analysis of yeast chromosome sizes was done as described by Maringele and Lydall [77] with minor modifications. Cells grown overnight were embedded in 20 µl plugs of low melting point agarose and digested with Zymolyase followed by Proteinase K and RNase. Liberated chromosomes were separated on CHEF Mapper® XA Pulsed Field Electrophoresis System (Bio-Rad) for 22 h at 5.9 V/cm and 12°C with angle set to 120°, switch time to 60 s and 80 s with ramping 0.8. Separated chromosomes were stained with ethidium bromide illuminated with 302 nm UV light and digitized with a charge-coupled device camera.

## Supporting Information

Figure S1 **The strategy of microarray-based genome-wide SLM screen using** ***CAN1/can1Δ*** **derivative homodiploid YKO collection.**
(PDF)

Figure S2 **The strategy of microarray-based genome-wide SLM screen using** ***URA3/ura3Δ*** **derivative homodiploid YKO collection.**
(PDF)

Figure S3 **The strategy of microarray-based genome-wide SLM screen for SLM at** ***MATa/MATα*** **loci.**
(PDF)

Figure S4 **The changes of DNA content in cells of 2n strain during prolonged growth.** DNA content analysis was done after: 0, 50, 100, 160, 240 and 320 generations. Please note that “0” represents the starting point of the experiment. In fact, as we estimate, at this point the clones originating from the single zygotes had already grown for about 50 generations. The data for 20 independently obtained clones are shown. Propidium iodide stained cells were analyzed by FACS as described in Materials and Methods.
(TIF)

Figure S5 **The changes of DNA content in cells of 2n ctf18 strain during prolonged growth.** DNA content analysis was done after: 0, 50, 100, 160, 240 and 320 generations. Please note that “0” represents the starting point of the experiment. In fact, as we estimate, at this point the clones originating from the single zygotes had already grown for about 50 generations. The data for 20 independently obtained clones are shown. Propidium iodide stained cells were analyzed by FACS as described in Materials and Methods.
(TIF)

Figure S6 **The changes of DNA content in cells of 2n ndt80 strain during prolonged growth.** DNA content analysis was done after: 0, 50, 100, 160, 240 and 320 generations. Please note that “0” represents the starting point of the experiment. In fact, as we estimate, at this point the clones originating from the single zygotes had already grown for about 50 generations. The data for 20 independently obtained clones are shown. Propidium iodide stained cells were analyzed by FACS as described in Materials and Methods.
(TIF)

Figure S7 **The changes of DNA content in cells of 2n ndt80 ctf18 strain during prolonged growth.** DNA content analysis was done after: 0, 50, 100, 160, 240 and 320 generations. Please note that “0” represents the starting point of the experiment. In fact, as we estimate, at this point the clones originating from the single zygotes had already grown for about 50 generations. The data for 20 independently obtained clones are shown. Propidium iodide stained cells were analyzed by FACS as described in Materials and Methods.
(TIF)

Figure S8 **SMC complexes and their interactomes connected with genome stability.** Genes that appeared in our SLM screens with high LogRatio are shown in green. Genes with high LogRatio and low p-value present on final 249 SLM gene list (see Table S2) are underlined. Trapezoid features denote regulatory genes. SMC complexes are surrounded with green ovals. The diagram was prepared using Pathfinder tool from Biobase Knowledge Library (http://www.biobase-international.com) and supplemented with SGD BIOGRID information (http://thebiogrid.org).
(TIF)

Table S1 **Summary of results of all three large scale SLM screens.**
(PDF)

Table S2 **249 strains with elevated SLM.** The table lists the deletion strains with elevated SLM at both *CAN1* and *URA3 loci*, or with elevated SLM at *CAN1* or *URA3 locus* that mate with *MATα* or *MATa*; potential mutator strains. Strains in particular groups are listed in order of decreasing phenotype intensity. The descriptions of genes with two most prevalent cellular localizations, nuclear and mitochondrial, are highlighted in green and yellow respectively.
(PDF)

Table S3 **Correlation between the results of large scale SLM screens and semi-quantitative individual SLM assay.**
(PDF)

Table S4 **Subcellular localization of 249 ORFs selected in our genome-wide SLM screens.**
(PDF)

Materials and Methods S1 (DOC)
