# Correction: Dipoid-Specific Genome Stability Genes of *S. cerevisiae*: Genomic Screen Reveals Haploidization as an Escape from Persisting DNA Rearrangement Stress

**DOI:** 10.1371/annotation/77daccf9-9976-4d0e-b666-35a900cb2d17

**Published:** 2011-11-08

**Authors:** Malgorzata Alabrudzinska, Marek Skoneczny, Adrianna Skoneczna

The correct title is: "Diploid-Specific Genome Stability Genes of S. cerevisiae: Genomic Screen Reveals Haploidization as an Escape from Persisting DNA Rearrangement Stress."

The correct citation is: Alabrudzinska M, Skoneczny M, Skoneczna A (2011) Diploid-Specific Genome Stability Genes of S. cerevisiae: Genomic Screen Reveals Haploidization as an Escape from Persisting DNA Rearrangement Stress. PLoS ONE 6(6): e21124. doi:10.1371/journal.pone.0021124

