# Supplementary material for: Dipoid-Specific Genome Stability Genes of S. cerevisiae: Genomic Screen Reveals Haploidization as an Escape from Persisting DNA Rearrangement Stress
Source: PLoS One. 2011 Jun 17;6(6):e21124. doi: 10.1371/journal.pone.0021124 (PMC3117874; doi:10.1371/journal.pone.0021124)
Supplement: Figure S2 — The strategy of microarray-based genome-wide SLM screen using URA3/ura3Δ derivative homodiploid YKO collection. (PDF) [file pone.0021124.s002.pdf]

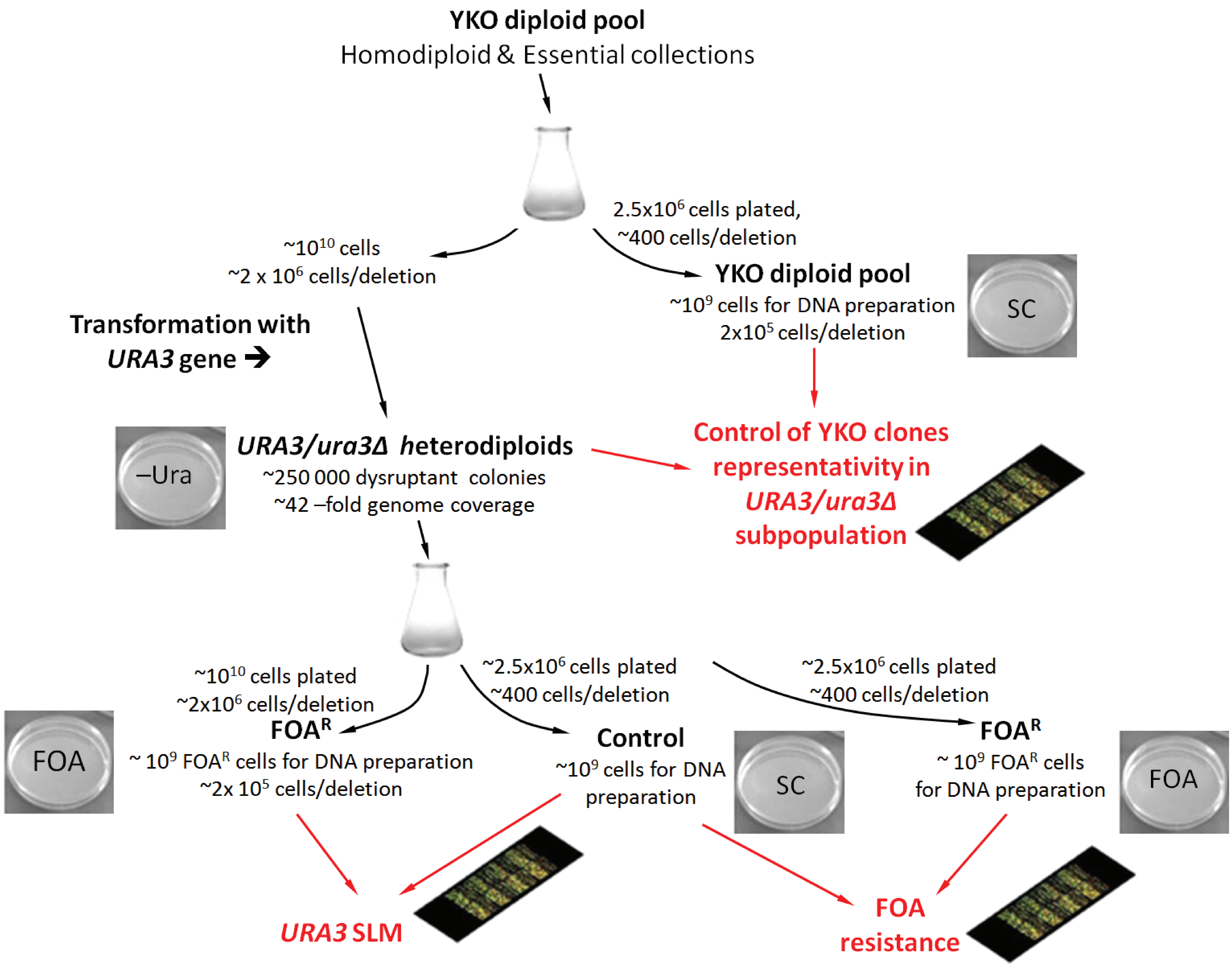

Figure S2. The strategy of microarray-based genome-wide SLM screen using *URA3/ura3Δ* derivative homodiploid YKO collection.
