# Supplementary figures and images for: Dipoid-Specific Genome Stability Genes of S. cerevisiae: Genomic Screen Reveals Haploidization as an Escape from Persisting DNA Rearrangement Stress
Source: PLoS One. 2011 Jun 17;6(6):e21124. doi: 10.1371/journal.pone.0021124 (PMC3117874; doi:10.1371/journal.pone.0021124)

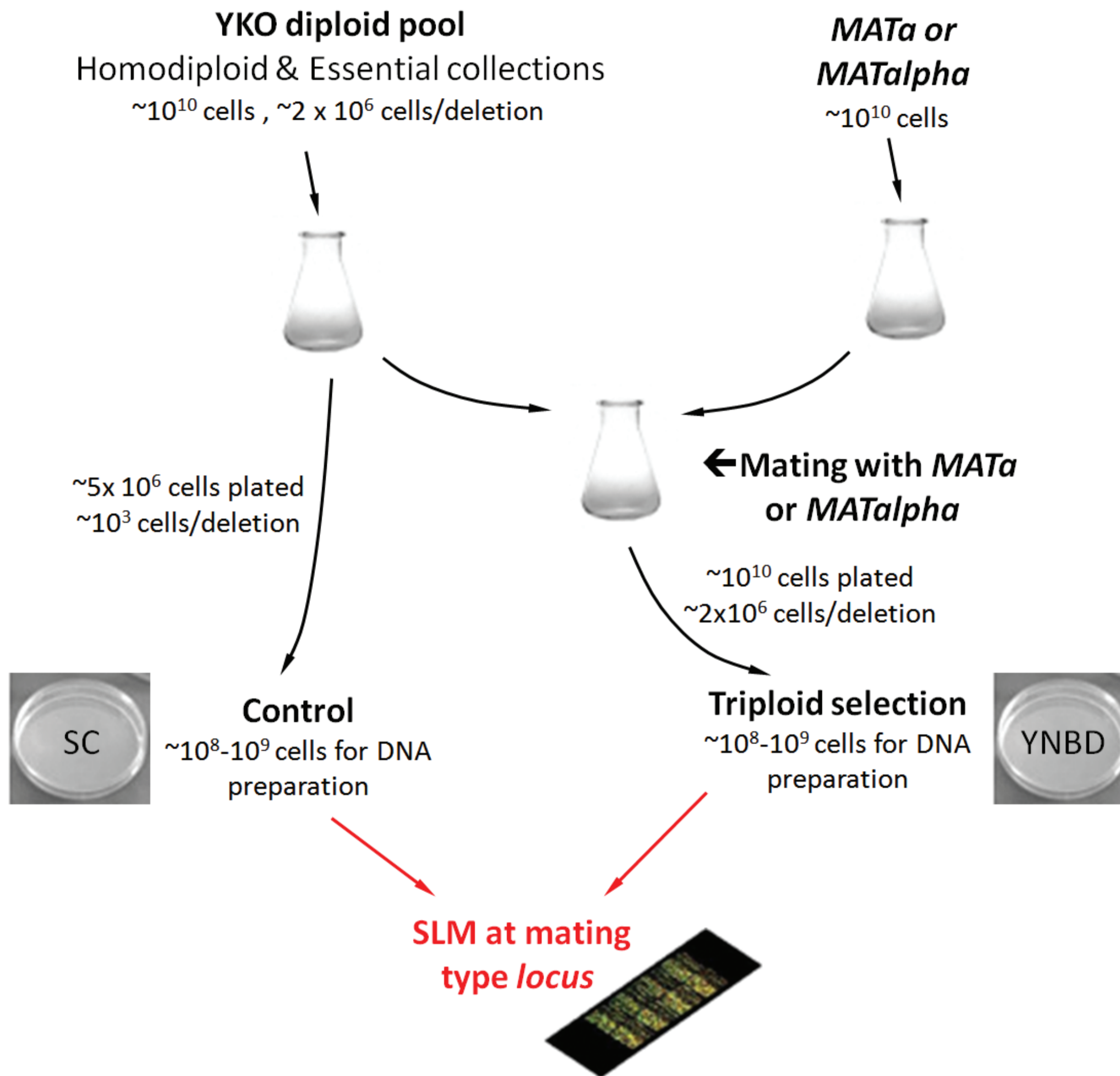

**Figure S3.** The strategy of microarray-based genome-wide screen for SLM at *MATa*/*MATα* loci.

Supplement: Figure S3 — The strategy of microarray-based genome-wide SLM screen for SLM at MATa/MATα loci. (PDF) [file pone.0021124.s003.pdf]

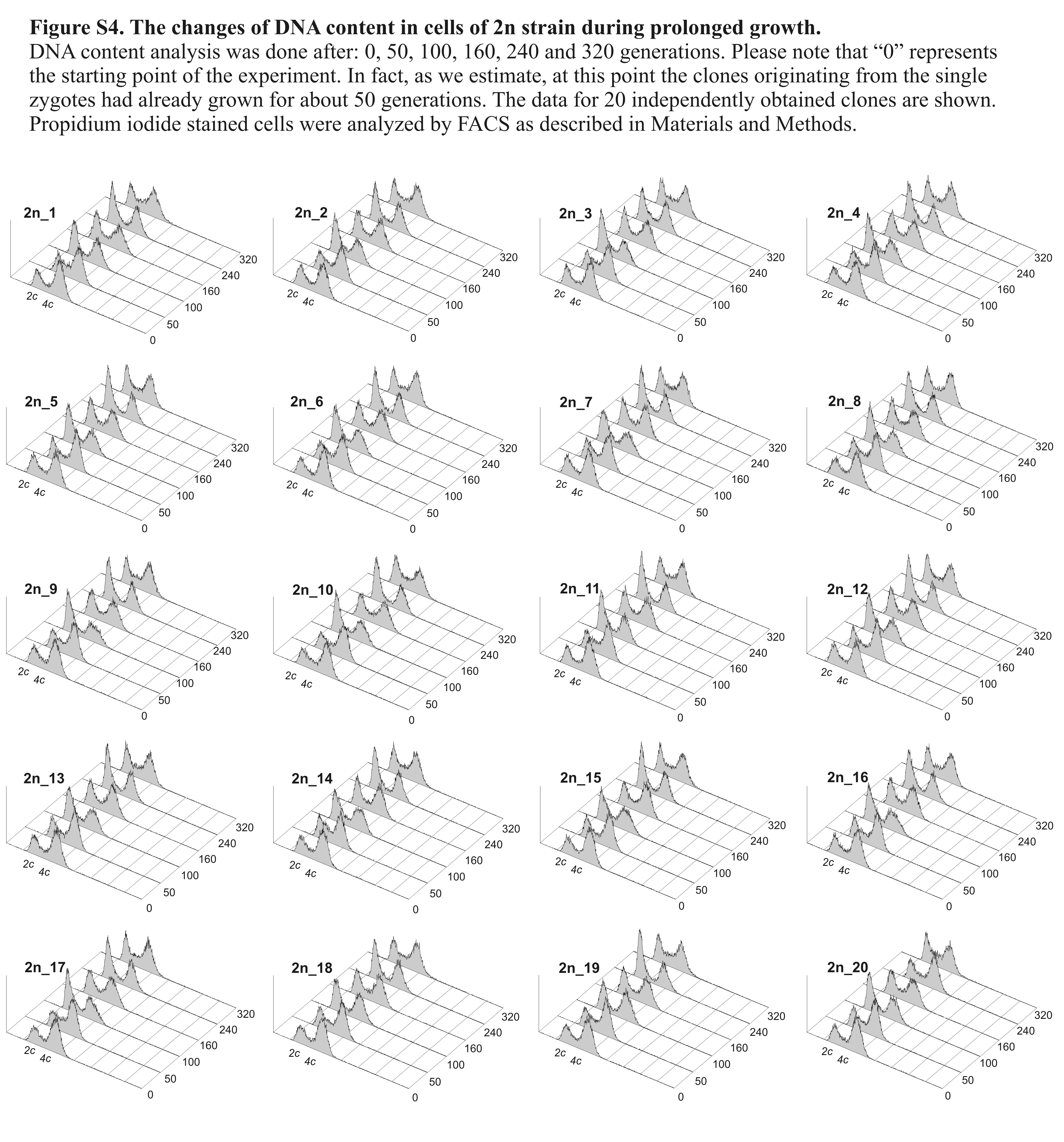

Supplement: Figure S4 — The changes of DNA content in cells of 2n strain during prolonged growth. DNA content analysis was done after: 0, 50, 100, 160, 240 and 320 generations. Please note that “0” represents the starting point of the experiment. In fact, as we estimate, at this point the clones originating from the single zygotes had already grown for about 50 generations. The data for 20 independently obtained clones are shown. Propidium iodide stained cells were analyzed by FACS as described in Materials and Methods. (TIF) [file pone.0021124.s004.tif]

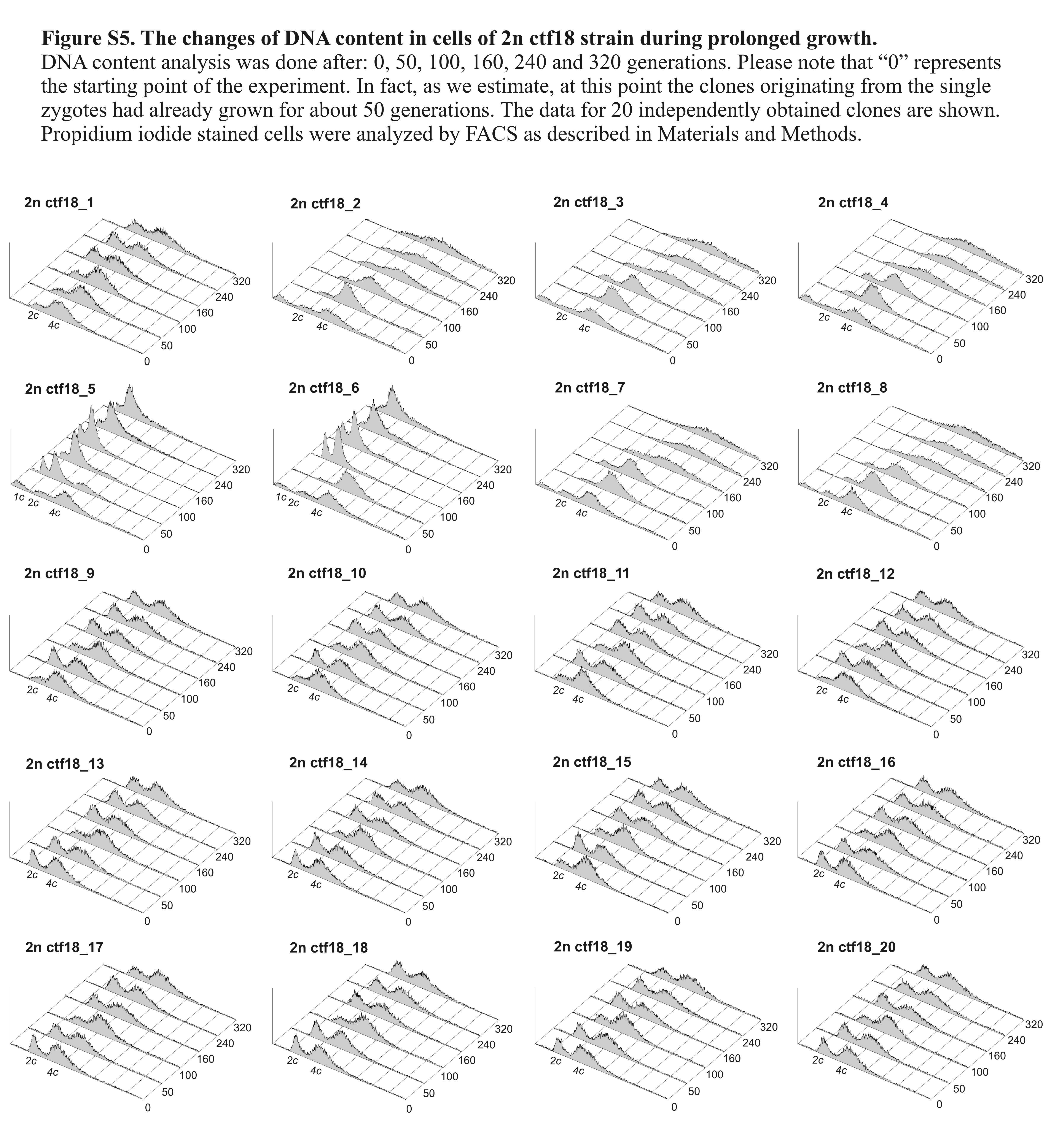

Supplement: Figure S5 — The changes of DNA content in cells of 2n ctf18 strain during prolonged growth. DNA content analysis was done after: 0, 50, 100, 160, 240 and 320 generations. Please note that “0” represents the starting point of the experiment. In fact, as we estimate, at this point the clones originating from the single zygotes had already grown for about 50 generations. The data for 20 independently obtained clones are shown. Propidium iodide stained cells were analyzed by FACS as described in Materials and Methods. (TIF) [file pone.0021124.s005.tif]

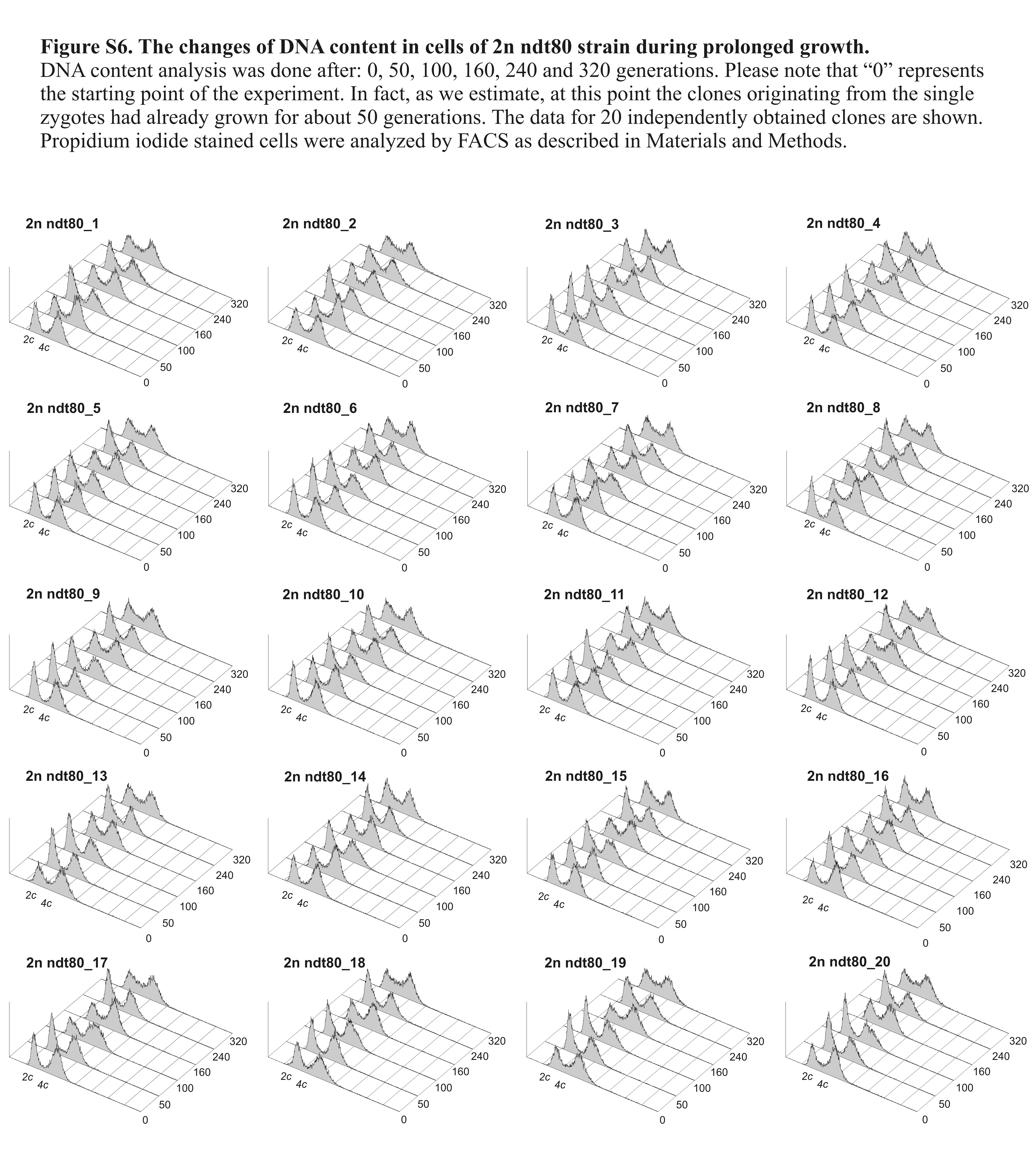

Supplement: Figure S6 — The changes of DNA content in cells of 2n ndt80 strain during prolonged growth. DNA content analysis was done after: 0, 50, 100, 160, 240 and 320 generations. Please note that “0” represents the starting point of the experiment. In fact, as we estimate, at this point the clones originating from the single zygotes had already grown for about 50 generations. The data for 20 independently obtained clones are shown. Propidium iodide stained cells were analyzed by FACS as described in Materials and Methods. (TIF) [file pone.0021124.s006.tif]

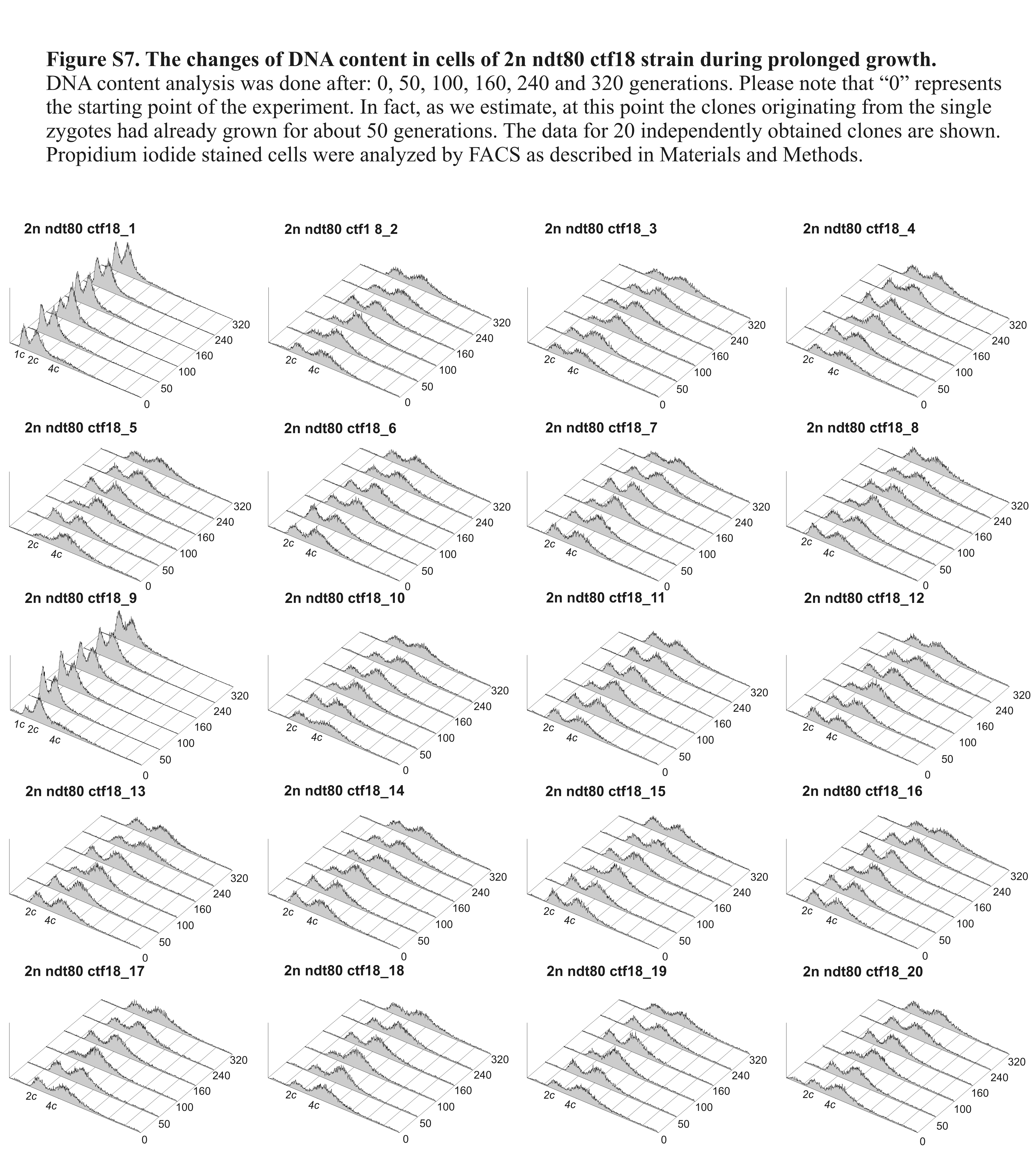

Supplement: Figure S7 — The changes of DNA content in cells of 2n ndt80 ctf18 strain during prolonged growth. DNA content analysis was done after: 0, 50, 100, 160, 240 and 320 generations. Please note that “0” represents the starting point of the experiment. In fact, as we estimate, at this point the clones originating from the single zygotes had already grown for about 50 generations. The data for 20 independently obtained clones are shown. Propidium iodide stained cells were analyzed by FACS as described in Materials and Methods. (TIF) [file pone.0021124.s007.tif]

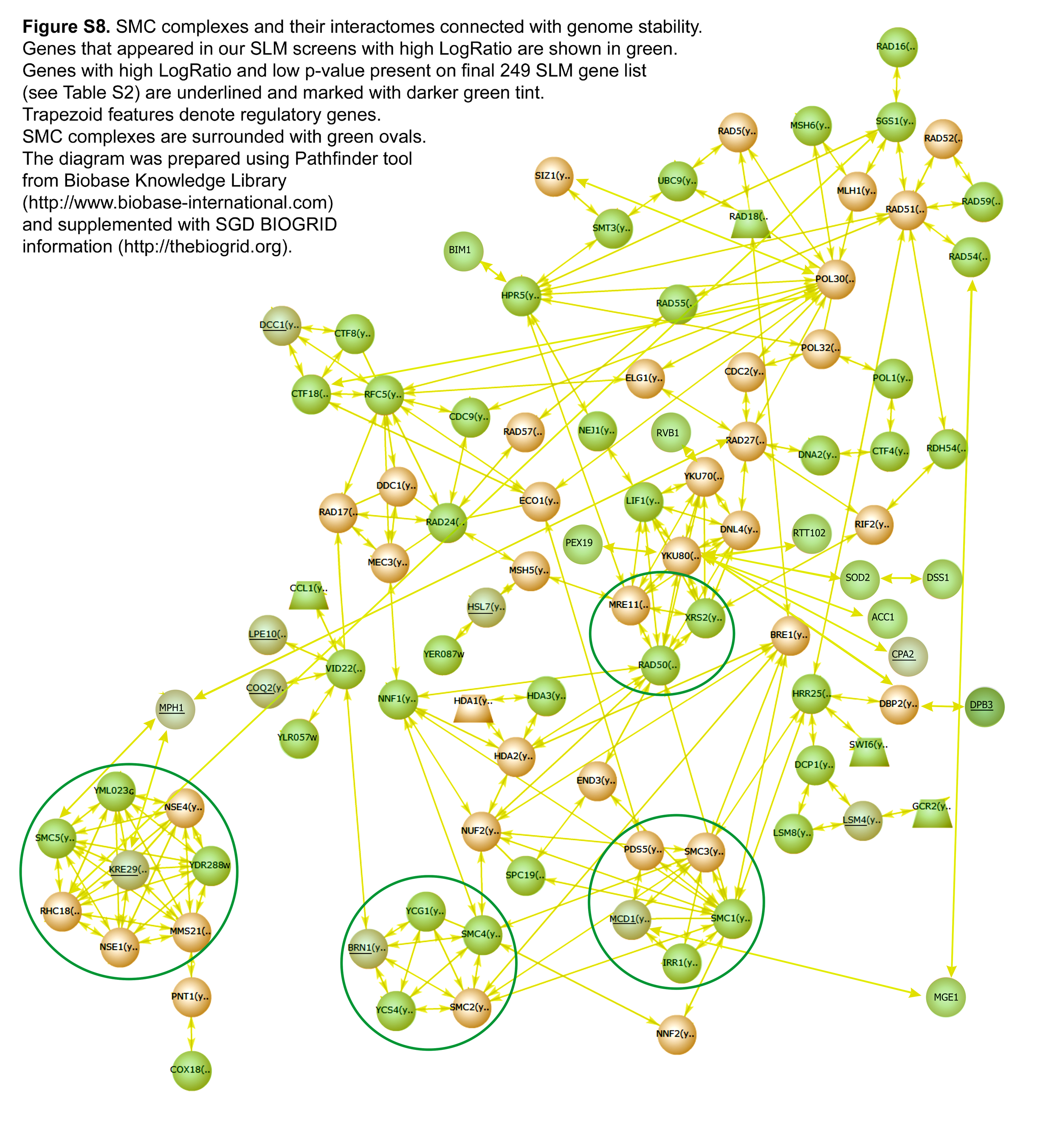

Supplement: Figure S8 — SMC complexes and their interactomes connected with genome stability. Genes that appeared in our SLM screens with high LogRatio are shown in green. Genes with high LogRatio and low p-value present on final 249 SLM gene list (see Table S2) are underlined. Trapezoid features denote regulatory genes. SMC complexes are surrounded with green ovals. The diagram was prepared using Pathfinder tool from Biobase Knowledge Library (http://www.biobase-international.com) and supplemented with SGD BIOGRID information (http://thebiogrid.org). (TIF) [file pone.0021124.s008.tif]
