# Supplementary material for: Dipoid-Specific Genome Stability Genes of S. cerevisiae: Genomic Screen Reveals Haploidization as an Escape from Persisting DNA Rearrangement Stress
Source: PLoS One. 2011 Jun 17;6(6):e21124. doi: 10.1371/journal.pone.0021124 (PMC3117874; doi:10.1371/journal.pone.0021124)
Supplement: Table S1 — Summary of results of all three large scale SLM screens. (PDF) [file pone.0021124.s009.pdf]

Table S1. Summary of results of all three large scale SLM screens.

		Strains with p-val<0.1		Strains with significant LogRatio		Strains with p-val<0.1 and significant LogRatio	
		Strain count	% of total*	Strain count	% of total*	Strain count	% of total*
1	Strains with elevated SLM at *CAN1 locus* (LogRatio SLM *CAN1* > 2)	4610	80,74	1250	21,89	748	13,10
2	Strains with elevated SLM at *URA3 locus* (LogRatio SLM *URA3* > 2)	2115	37,04	2670	46,76	998	17,48
3	Strains with elevated SLM at both *loci CAN1* and *URA3* [(LogRatio SLM *CAN1* > 2) AND (LogRatio SLM *URA3* > 2)]	**1694**	**29,67**	**757**	**13,26**	**158**	**2,77**
4	Strains which mate as *MATa* (LogRatio cross with *MATα* > 1)	5089	89,12	637	11,16	458	8,02
5	Strains which mate as *MATα* (LogRatio cross with *MATa* > 1)	4894	85,71	693	12,14	351	6,15
6	Strains which mate with *MATa* or *MATα* [(LogRatio cross with *MATα* > 1) OR (LogRatio cross with *MATa* > 1)]	4563	79,91	825	14,45	637	11,16
7	Strains with elevated SLM at *URA3 locus*, which mate with *MATα* or *MATa*, but with slight if any SLM at *CAN1 locus*, {[(LogRatio SLM *URA3* > 2) AND [(LogRatio cross with *MATα* > 1) OR (LogRatio cross with *MATa* > 1)] AND [LogRatio SLM *CAN1* < 2]}	**1442**	**25,25**	**264**	**4,62**	**74**	**1,30**
8	Strains with elevated SLM at *CAN1 locus* which mate with *MATα* or *MATa*, but slight if any SLM at *URA3 locus*, **might be haploid** {[(LogRatio SLM *CAN1* > 2) AND [(LogRatio cross with *MATα* > 1) OR (LogRatio cross with *MATa* > 1)] AND [LogRatio SLM *URA3* < 2]}	**1442**	**25,25**	**97**	**1,70**	**17**	**0,30**
9	Strains with elevated SLM at both *loci CAN1* and *URA3*, or with elevated SLM at *CAN1* or *URA3 loci*, which mate with *MATα* or *MATa*; **potential mutator strains** (3 OR 7 OR 8)	**1694**	**29,67**	**1118**	**19,58**	**249**	**4,36**
10	Strains with significant signal in all experiments**, mutators** {[(LogRatio SLM *CAN1* > 2) AND (LogRatio SLM *URA3* > 2)] AND [(LogRatio cross with *MATα* > 1) OR (LogRatio cross with *MATa* > 1)]}	**1442**	**25,25**	**146**	**2,56**	**21**	**0,37**

*) – Relative to total number of 5710 strains included in the analysis.
