# Supplementary material for: Dipoid-Specific Genome Stability Genes of S. cerevisiae: Genomic Screen Reveals Haploidization as an Escape from Persisting DNA Rearrangement Stress
Source: PLoS One. 2011 Jun 17;6(6):e21124. doi: 10.1371/journal.pone.0021124 (PMC3117874; doi:10.1371/journal.pone.0021124)
Supplement: Table S2 — 249 strains with elevated SLM. The table lists the deletion strains with elevated SLM at both CAN1 and URA3 loci, or with elevated SLM at CAN1 or URA3 locus that mate with MATα or MATa; potential mutator strains. Strains in particular groups are listed in order of decreasing phenotype intensity. The descriptions of genes with two most prevalent cellular localizations, nuclear and mitochondrial, are highlighted in green and yellow respectively. (PDF) [file pone.0021124.s010.pdf]

Table S2. 249 strains with elevated SLM at both *CAN1* and *URA3 loci*, or with elevated SLM at *CAN1* or *URA3 locus* that mate with *MATα* or *MATa*; potential mutator strains. Strains in particular groups are listed in order of decreasing phenotype intensity. The descriptions of genes with two most prevalent cellular localizations, nuclear and mitochondrial, are highlighted in green and yellow respectively.

Strains with significant signal in all experiments, **mutators**

{[(LogRatio SLM *CAN1* >2) AND (LogRatio SLM *URA3* > 2)] AND [(LogRatio cross with *MATα* > 1) OR (LogRatio cross with *MATa* > 1)]}

ORF	Name	SLM at *CAN1* locus		SLM at *URA3* locus		cross with *MATα*		cross with *MATa*		Description as in SGD	Localization	Status	Collection	Instability phenotypes	Genotoxic stress sensitivity	Other genome related phenotypes
		LogR	p-val	LogR	p-val	LogR	p-val	LogR	p-val							
YGL236C	*MTO1*	12,721	0,000	6,079	0,093	6,403	0,000	-3,226	0,000	Mitochondrial protein, forms a heterodimer complex with Mss1p that performs the 5-carboxymethylaminomethyl modification of the wobble uridine base in mitochondrial tRNAs; required for respiration in paromomycin-resistant 15S rRNA mutants	mitochondrion	Verified	Homodiploid	RMCD, mtCM		SPO*
YMR078C	*CTF18*	12,493	0,001	5,627	0,004	5,293	0,000	-2,245	0,061	Subunit of a complex with Ctf8p that shares some subunits with Replication Factor C and is required for sister chromatid cohesion; may have overlapping functions with Rad24p in the DNA damage replication checkpoint	nucleus	Verified	Homodiploid	ALF, CM, CSA, CS, TL	HU, MMS, Phleomycin, Camptothecin, Bleomycin A2, Cisplatin,	SPO*, Benomyl
YDR281C	*PHM6*	12,129	0,000	2,010	0,053	5,582	0,002	-3,847	0,040	Protein of unknown function, expression is regulated by phosphate levels	vacuole	Verified	Homodiploid	RMCD		SPO*
YIL039W	*TED1*	11,652	0,000	5,129	0,000	5,954	0,000	-3,837	0,000	Conserved phosphoesterase domain-containing protein that acts together with Emp24p/Erv25p in cargo exit from the ER; deletion confers sensitivity to 4-(N-(S-glutathionylacetyl)amino) phenylarsenoxide (GSAO)	Endoplasmic Reticulum	Uncharacterized	Homodiploid			SPO*
YLR137W	*YLR137W*	9,806	0,000	3,392	0,048	-5,429	0,000	3,706	0,000	Putative protein of unknown function	unknown	Uncharacterized	Homodiploid			
YBR078W	*ECM33*	9,589	0,093	2,077	0,045	1,681	0,000	-3,175	0,001	GPI-anchored protein of unknown function, has a possible role in apical bud growth; GPI-anchoring on the plasma membrane crucial to function; phosphorylated in mitochondria; similar to Sps2p and Pst1p	mitochondrion	Verified	Homodiploid	RMCD	Doxorubicin	
YJR037W	*YJR037W*	9,046	0,003	2,619	0,095	3,573	0,000	-5,194	0,000	Dubious open reading frame unlikely to encode a protein, based on available experimental and comparative sequence data; partially overlaps verified gene HUL4/YJR036C; deletion mutant has decreased spore survival in Drosophila feces	unknown	Dubious	Homodiploid			
YML042W	*CAT2*	7,032	0,000	2,794	0,070	4,343	0,000	-4,789	0,000	Carnitine acetyl-CoA transferase present in both mitochondria and peroxisomes, transfers activated acetyl groups to carnitine to form acetylcarnitine which can be shuttled across membranes	mitochondrion, peroxisome	Verified	Homodiploid			
YER175C	*TMT1*	6,746	0,003	4,216	0,045	2,744	0,000	-3,920	0,000	Trans-aconitate methyltransferase, cytosolic enzyme that catalyzes the methyl esterification of 3-isopropylmalate, an intermediate of the leucine biosynthetic pathway, and trans-aconitate, which inhibits the citric acid cycle	cytoplasm	Verified	Homodiploid			
YIL066C	*RNR3*	6,406	0,001	3,042	0,044	2,949	0,000	4,476	0,000	One of two large regulatory subunits of ribonucleotide-diphosphate reductase; the RNR complex catalyzes rate-limiting step in dNTP synthesis, regulated by DNA replication and DNA damage checkpoint pathways via localization of small subunits	mitochondrion, cytoplasm	Verified	Homodiploid		Tirapazamine	
YDR334W	*SWR1*	4,010	0,018	2,631	0,007	2,560	0,000	-6,000	0,000	Swi2/Snf2-related ATPase that is the structural component of the SWR1 complex, which exchanges histone variant H2AZ (Htz1p) for chromatin-bound histone H2A	nucleus	Verified	Homodiploid	BIM	HU	Benomyl
YOR311C	*HSD1*	4,002	0,065	3,463	0,075	2,968	0,000	-4,687	0,000	Diacylglycerol kinase, localized to the endoplasmic reticulum (ER); overproduction induces enlargement of ER-like membrane structures and suppresses a temperature-sensitive sly1 mutation; contains a CTP transferase domain	Endoplasmic Reticulum	Verified	Homodiploid			
YNL032W	*SIW14*	3,920	0,004	7,242	0,002	1,523	0,000	-3,299	0,000	Tyrosine phosphatase that plays a role in actin filament organization and endocytosis; localized to the cytoplasm	cytoplasm	Verified	Homodiploid	TL		
YOL087C	*YOL087C*	3,649	0,005	6,076	0,055	-2,273	0,001	2,853	0,000	Putative protein of unknown function; green fluorescent protein (GFP)-fusion protein localizes to the cytoplasm; deletion mutant is sensitive to various chemicals including phenanthroline, sanguinarine, and nordihydroguaiaretic acid	cytoplasm	Uncharacterized	Homodiploid			

YCL014W	*BUD3*	3,621	0,002	2,222	0,048	**1,647**	0,001	-3,141	0,000	Protein involved in bud-site selection and required for axial budding pattern; localizes with septins to bud neck in mitosis and may constitute an axial landmark for next round of budding	bud neck	Verified	Homodiploid			
YNR065C	*YNR065C*	3,264	0,051	2,539	0,025	1,138	0,000	-2,210	0,000	Protein of unknown function; protein-protein interactions suggest a possible role in actin patch formation; YNR065C is not an essential gene	actin path	Uncharacterized	Homodiploid			
YGL091C	*NBP35*	3,025	0,067	3,036	0,072	-3,664	0,000	**1,690**	0,033	Essential iron-sulfur cluster binding protein localized in the cytoplasm; forms a complex with Cfd1p that is involved in iron-sulfur protein assembly in the cytosol; similar to P-loop NTPases	cytoplasm	Verified	Essential			
YBL096C	*YBL096C*	2,482	0,001	3,806	0,090	-4,425	0,000	1,260	0,000	Non-Essential protein of unknown function	unknown	Dubious	Homodiploid			
YNL076W	*MKS1*	2,380	0,001	3,543	0,016	**2,160**	0,000	-0,577	0,026	Pleiotropic negative transcriptional regulator involved in Ras-CAMP and lysine biosynthetic pathways and nitrogen regulation; involved in retrograde (RTG) mitochondria-to-nucleus signaling	nucleus, mitochondrion	Verified	Homodiploid		HU	CCP
YDR067C	*YDR067C*	2,378	0,001	6,168	0,019	-5,538	0,000	**2,589**	0,000	Cytoplasmic protein required for replication of Brome mosaic virus in *S. cerevisiae*, which is a model system for studying replication of positive-strand RNA viruses in their natural hosts	cytoplasm	Uncharacterized	Homodiploid			
YKL160W	*ELF1*	2,358	0,007	5,029	0,025	**4,944**	0,000	-3,594	0,000	Transcription elongation factor that contains a conserved zinc finger domain; implicated in the maintenance of proper chromatin structure in actively transcribed regions; deletion inhibits Brome mosaic virus (BMV) gene expression	nucleus	Verified	Homodiploid	TE-T		

Strains with elevated SLM at both CAN1 and URA3 loci

[(LogRatio SLM CAN1 > 2) AND (LogRatio SLM URA3 > 2)]

ORF	Name	SLM at *CAN1* locus		SLM at *URA3* locus		cross with *MATalpha*		cross with *MATa*		Description	Localization	Status	Collection	Instability phenotypes	Genotoxic stress sensitivity	Other genome related phenotypes
		LogR	p-val	LogR	p-val	LogR	p-val	LogR	p-val							
YNL099C	*OCA1*	3,493	0,014	7,453	0,006	-2,259	0,009	-2,374	0,018	Putative protein tyrosine phosphatase, required for cell cycle arrest in response to oxidative damage of DNA	nucleus	Verified	Homodiploid			
YJR002W	*MPP10*	2,458	0,003	7,433	0,078	-4,301	0,001	-3,263	0,000	Component of the SSU processome and 90S preribosome, required for pre-18S rRNA processing, interacts with and controls the stability of Imp3p and Imp4p, Essential for viability; similar to human Mpp10p	nucleolus	Verified	Essential			
YDR265W	*PEX10*	4,228	0,024	6,821	0,091	-5,115	0,000	-6,009	0,000	Peroxisomal membrane E3 ubiquitin ligase required for for Ubc4p-dependent Pex5p ubiquitination and peroxisomal matrix protein import; contains zinc-binding RING domain; mutations in human homolog cause various peroxisomal disorders	peroxisome	Verified	Homodiploid			
YDR408C	*ADE8*	3,845	0,003	6,694	0,006	-3,455	0,002	-4,365	0,000	Phosphoribosyl-glycinamide transformylase, catalyzes a step in the 'de novo' purine nucleotide biosynthetic pathway	nucleus, cytoplasm	Verified	Homodiploid	ALF, HAP		
YBR110W	*ALG1*	2,948	0,014	6,527	0,072	-3,426	0,008	-3,247	0,004	Mannosyltransferase, involved in asparagine-linked glycosylation in the endoplasmic reticulum (ER); Essential for viability, mutation is functionally complemented by human ortholog	Endoplasmic Reticulum	Verified	Essential			
YDL209C	*CWC2*	3,484	0,009	6,499	0,055	-4,016	0,050	-3,798	0,003	Protein involved in pre-mRNA splicing, component of a complex containing Cef1p; interacts with Prp19p; contains an RNA recognition motif; has similarity to *S. pombe* Cwf2p	nucleus	Verified	Essential	CS		
YBR087W	*RFC5*	2,892	0,001	6,370	0,084	-3,025	0,006	-0,052	0,929	Subunit of heteropentameric Replication factor C (RF-C), which is a DNA binding protein and ATPase that acts as a clamp loader of the proliferating cell nuclear antigen (PCNA) processivity factor for DNA polymerases delta and epsilon	nucleus	Verified	Essential	GCR, CSA, CM, MF		

YFL007W	***BLM10***	**4,394**	**0,004**	**6,243**	**0,043**	-2,485	0,019	-3,173	0,000	Proteosome activator subunit; found in association with core particles, with and without the 19S regulatory particle; required for resistance to bleomycin, may be involved in protecting against oxidative damage; similar to mammalian PA200	proteasome	Verified	Homodiploid		Phleomycin	
YKL053C-A	***MDM35***	**3,043**	**0,003**	**6,084**	**0,043**	-0,947	0,350	**2,306**	0,066	Mitochondrial intermembrane space cysteine motif protein; mutation affects mitochondrial distribution and morphology	mitochondrion	Verified	Homodiploid		Doxorubicin	
YML015C	***TAF11***	**3,656**	**0,011**	**6,042**	**0,089**	-1,086	0,253	-3,242	0,004	TFIID subunit (40 kDa), involved in RNA polymerase II transcription initiation, similar to histone H3 with atypical histone fold motif of Spt3-like transcription factors	nucleus	Verified	Essential			
YHL029C	***YHL029C***	**2,688**	**0,027**	**5,954**	**0,012**	-4,626	0,000	-0,751	0,000	Cytoplasmic protein required for replication of Brome mosaic virus in *S. cerevisiae*, which is a model system for studying replication of positive-strand RNA viruses in their natural hosts	cytoplasm	Uncharacterized	Homodiploid			
YDR164C	***SEC1***	**2,289**	**0,011**	**5,918**	**0,067**	-3,720	0,001	-2,172	0,010	Sm-like protein involved in docking and fusion of exocytic vesicles through binding to assembled SNARE complexes at the membrane; localization to sites of secretion (bud neck and bud tip) is dependent on SNARE function	bud neck	Verified	Essential			
YDR177W	***UBC1***	**3,103**	**0,097**	**5,790**	**0,050**	-4,939	0,002	-3,122	0,046	Ubiquitin-conjugating enzyme that mediates selective degradation of short-lived and abnormal proteins; plays a role in vesicle biogenesis and ER-associated protein degradation (ERAD); component of the cellular stress response	proteasome	Verified	Essential			
YDR060W	***MAK21***	**2,562**	**0,015**	**5,787**	**0,076**	-5,287	0,017	-2,502	0,042	Constituent of 66S pre-ribosomal particles, required for large (60S) ribosomal subunit biogenesis; involved in nuclear export of pre-ribosomes; required for maintenance of dsRNA virus; homolog of human CAATT-binding protein	nucleolus	Verified	Essential			
YDL003W	***MCD1***	**3,181**	**0,004**	**5,489**	**0,003**	-3,948	0,000	-4,530	0,001	Essential protein required for sister chromatid cohesion in mitosis and meiosis; subunit of the cohesin complex; expression is cell cycle regulated and peaks in S phase	nucleus	Verified	Essential		Camptothecin	
YPR168W	***NUT2***	**4,379**	**0,002**	**5,401**	**0,008**	-5,367	0,000	-3,485	0,000	Subunit of the RNA polymerase II mediator complex; associates with core polymerase subunits to form the RNA polymerase II holoenzyme; required for transcriptional activation and has a role in basal transcription	nucleus	Verified	Essential			
YPR107C	***YTH1***	**3,519**	**0,035**	**5,303**	**0,003**	-3,818	0,001	-1,039	0,019	Essential RNA-binding component of cleavage and polyadenylation factor, contains five zinc fingers; required for pre-mRNA 3'-end processing and polyadenylation	nucleus	Verified	Essential			
YML079W	***YML079W***	**2,859**	**0,000**	**5,142**	**0,096**	-4,203	0,023	-1,898	0,054	Non-Essential protein of unknown function with structural resemblance to plant storage and ligand binding proteins (canavalin, glycinin, auxin binding protein) and to some enzymes (epimerase, germin); localizes to the nucleus and cytoplasm	nucleus, cytoplasm	Uncharacterized	Homodiploid			
YMR111C	***YMR111C***	**6,658**	**0,002**	**5,118**	**0,032**	-5,807	0,000	-0,678	0,010	Protein of unknown function; green fluorescent protein (GFP)-fusion protein localizes to the nucleus; YMR111C is not an Essential gene	nucleus	Uncharacterized	Homodiploid			
YBR059C	***AKL1***	**3,436**	**0,001**	**4,927**	**0,046**	-4,282	0,000	-0,967	0,003	Ser-Thr protein kinase, member (with Ark1p and Prk1p) of the Ark kinase family; involved in endocytosis and actin cytoskeleton organization	unknown	Verified	Homodiploid		Doxorubicin, Tirapazamine	
YML127W	***RSC9***	**3,883**	**0,038**	**4,763**	**0,040**	-6,356	0,008	-5,377	0,000	Component of the RSC chromatin remodeling complex; DNA-binding protein involved in the synthesis of rRNA and in transcriptional repression and activation of genes regulated by the Target of Rapamycin (TOR) pathway	nucleus	Verified	Essential			
YHR006W	***STP2***	**3,491**	**0,000**	**4,753**	**0,093**	-0,552	0,015	0,679	0,006	Transcription factor, activated by proteolytic processing in response to signals from the SPS sensor system for external amino acids; activates transcription of amino acid permease genes	nucleus	Verified	Homodiploid			

YLR222C	*UTP13*	4,130	0,006	4,683	0,086	-5,523	0,001	-5,374	0,000	Nucleolar protein, component of the small subunit (SSU) processome containing the U3 snoRNA that is involved in processing of pre-18S rRNA	nucleolus	Verified	Essential	CS, MR	HU	Benomyl
YAL055W	*PEX22*	2,763	0,000	4,635	0,016	-4,246	0,004	-1,696	0,012	Putative peroxisomal membrane protein required for import of peroxisomal proteins, functionally complements a *Pichia pastoris* pex22 mutation	peroxisome	Verified	Homodiploid			
YOR303W	*CPA1*	9,744	0,010	4,614	0,040	-3,987	0,000	-2,199	0,000	Small subunit of carbamoyl phosphate synthetase, which catalyzes a step in the synthesis of citrulline, an arginine precursor; translationally regulated by an attenuator peptide encoded by YOR302W within the CPA1 mRNA 5'-leader	carbamoyl-phosphate synthase complex	Verified	Homodiploid			
YHR018C	*ARG4*	8,512	0,026	4,612	0,058	-3,960	0,001	-2,178	0,000	Argininosuccinate lyase, catalyzes the final step in the arginine biosynthesis pathway	cytoplasm	Verified	Homodiploid	TE-T		
YPL060W	*LPE10*	4,812	0,002	4,607	0,079	-4,552	0,000	-4,665	0,001	Mitochondrial inner membrane magnesium transporter, involved in maintenance of magnesium concentrations inside mitochondria; indirectly affects splicing of group II introns; functionally and structurally related to Mrs2p	mitochondrion	Verified	Homodiploid			
YKR008W	*RSC4*	2,147	0,007	4,587	0,010	-4,914	0,000	-5,086	0,000	Component of the RSC chromatin remodeling complex; found in close proximity to nucleosomal DNA; displaced from the surface of nucleosomal DNA after chromatin remodeling	nucleus	Verified	Essential	TL		
YGR048W	*UFD1*	5,193	0,024	4,527	0,052	-3,011	0,004	-3,515	0,005	Protein that interacts with Cdc48p and Npl4p, involved in recognition of polyubiquitinated proteins and their presentation to the 26S proteasome for degradation; involved in transporting proteins from the ER to the cytosol	Endoplasmic Reticulum, cytoplasm	Verified	Essential			
YGR021W	*YGR021W*	2,107	0,000	4,480	0,094	-3,797	0,000	-3,000	0,002	Putative protein of unknown function; the authentic, non-tagged protein is detected in highly purified mitochondria in high-throughput studies	mitochondrion	Uncharacterized	Homodiploid			
YIR006C	*PAN1*	2,785	0,014	4,430	0,082	-3,698	0,001	-3,793	0,002	Part of actin cytoskeleton-regulatory complex Pan1p-Sla1p-End3p, associates with actin patches on the cell cortex; promotes protein-protein interactions Essential for endocytosis; previously thought to be a subunit of poly(A) ribonuclease	actin patches	Verified	Essential			
YER025W	*GCD11*	2,572	0,001	4,414	0,005	-3,662	0,000	-3,494	0,000	Gamma subunit of the translation initiation factor eIF2, involved in the identification of the start codon; binds GTP when forming the ternary complex with GTP and tRNAi-Met	cytoplasm, rybosome	Verified	Essential			
YGL233W	*SEC15*	3,247	0,029	4,380	0,054	-5,167	0,000	-3,751	0,000	Essential 113kDa subunit of the exocyst complex (Sec3p, Sec5p, Sec6p, Sec8p, Sec10p, Sec15p, Exo70p, and Exo84p), which mediates polarized targeting of vesicles to active sites of exocytosis; Sec15p associates with Sec4p and vesicles	vesicles	Verified	Essential			
YGR095C	*RRP46*	2,457	0,006	4,379	0,093	-3,419	0,000	-2,800	0,013	Protein involved in rRNA processing; component of the exosome 3->5 exonuclease complex	nuclear and cytoplasmic egsosom	Verified	Essential			
YJR141W	*YJR141W*	2,890	0,041	4,351	0,039	-5,082	0,003	-3,115	0,004	Essential protein of unknown function	unknown	Uncharacterized	Essential			
YGL086W	*MAD1*	2,410	0,028	4,270	0,054	-3,533	0,002	0,390	0,270	Coiled-coil protein involved in the spindle-assembly checkpoint; phosphorylated by Mps1p upon checkpoint activation which leads to inhibition of the activity of the anaphase promoting complex; forms a complex with Mad2p	nucleus	Verified	Homodiploid	BIM, CTF, CM, CS		Benomyl
YIL089W	*YIL089W*	2,026	0,000	4,191	0,095	-1,693	0,001	-4,266	0,000	Putative protein of unknown function	unknown	Uncharacterized	Homodiploid			
YDR217C	*RAD9*	5,075	0,034	4,185	0,082	-5,063	0,000	-5,156	0,000	DNA damage-dependent checkpoint protein, required for cell-cycle arrest in G1/S, intra-S, and G2/M; transmits checkpoint signal by activating Rad53p and Chk1p; hyperphosphorylated by Mec1p and Tel1p; potential Cdc28p substrate	nucleus	Verified	Homodiploid	BIM, ALF, GCR, CM, MF	HU, MMS, Phleomycin, Camptothecin, OXY, Metal, γR, UV	

YER038C	***KRE29***	**4,118**	**0,023**	**4,147**	**0,028**	-5,205	0,000	-4,913	0,000	Essential subunit of the Mms21-Smc5-Smc6 complex; protein of unknown function; required for growth and DNA repair; heterozygous mutant shows haploinsufficiency in K1 killer toxin resistance	nucleus	Verified	Essential			
YBR155W	***CNS1***	**2,940**	**0,022**	**4,127**	**0,029**	-4,750	0,000	-4,887	0,000	TPR-containing co-chaperone; binds both Hsp82p (Hsp90) and Ssa1p (Hsp70) and stimulates the ATPase activity of SSA1, ts mutants reduce Hsp82p function while over expression suppresses the phenotypes of an HSP82 ts allele and a cpr7 deletion	cytoplasm	Verified	Essential			
YAR031W	***PRM9***	**2,480**	**0,001**	**4,116**	**0,019**	-4,372	0,002	-4,321	0,004	Pheromone-regulated protein with 3 predicted transmembrane segments and an FF sequence, a motif involved in COPII binding; member of DUP240 gene family	Endoplasmic Reticulum, plasma membrane	Verified	Homodiploid		UV	
YPL181W	***CTI6***	**2,661**	**0,044**	**4,050**	**0,054**	-4,238	0,002	-4,006	0,000	Protein that relieves transcriptional repression by binding to the Cyc8p-Tup1p corepressor and recruiting the SAGA complex to the repressed promoter; contains a PHomodiploid finger domain	nucleus	V	Homodiploid		Metal, Doxorubicin	
YOR038C	***HIR2***	**2,465**	**0,001**	**4,020**	**0,010**	-3,125	0,001	0,390	0,056	Subunit of the HIR complex, a nucleosome assembly complex involved in regulation of histone gene transcription; recruits Swi-Snf complexes to histone gene promoters; promotes heterochromatic gene silencing with Asf1p	nucleus	Verified	Homodiploid	TE-T		
YJL024C	***APS3***	**3,637**	**0,060**	**4,012**	**0,007**	-3,108	0,002	-0,508	0,405	Small subunit of the clathrin-associated adaptor complex AP-3, which is involved in vacuolar protein sorting; related to the sigma subunit of the mammalian clathrin AP-3 complex; suppressor of loss of casein kinase 1 function	vacuole	Verified	Homodiploid		Metal	
YPL137C	***GIP3***	**3,467**	**0,013**	**3,998**	**0,065**	-3,268	0,000	-4,624	0,000	Glc7-interacting protein whose overexpression relocalizes Glc7p from the nucleus and prevents chromosome segregation; may interact with ribosomes, based on co-purification experiments	nucleus	Verified	Homodiploid			CCP
YDR313C	***PIB1***	**2,820**	**0,001**	**3,965**	**0,072**	-4,751	0,000	-3,527	0,000	RING-type ubiquitin ligase of the endosomal and vacuolar membranes, binds phosphatidylinositol(3)-phosphate; contains a FYVE finger domain	vacuole	Verified	Homodiploid			
YBR103W	***SIF2***	**6,165**	**0,090**	**3,943**	**0,016**	-3,644	0,000	-3,774	0,000	WD40 repeat-containing subunit of the Set3C histone deacetylase complex, which represses early/middle sporulation genes; antagonizes telomeric silencing; binds specifically to the Sir4p N-terminus	nucleus	Verified	Homodiploid			
YGR127W	***YGR127W***	**2,548**	**0,001**	**3,933**	**0,015**	-4,416	0,000	-2,006	0,000	Putative protein of unknown function; expression is regulated by Msn2p/Msn4p, indicating a possible role in stress response	unknown	Uncharacterized	Homodiploid			
YMR194C-A	***YMR194C-A***	**2,869**	**0,068**	**3,927**	**0,023**	-4,751	0,000	-5,110	0,000	Dubious open reading frame unlikely to encode a functional protein, based on available experimental and comparative sequence data	unknown	Dubious	Homodiploid			
YER112W	***LSM4***	**2,210**	**0,017**	**3,916**	**0,094**	-3,589	0,002	-3,099	0,001	Lsm (Like Sm) protein; part of heteroheptameric complexes (Lsm2p-7p and either Lsm1p or 8p): cytoplasmic Lsm1p complex involved in mRNA decay; nuclear Lsm8p complex part of U6 snRNP and possibly involved in processing tRNA, snoRNA, and rRNA	nucleus, cytoplasm, nucleolus	Verified	Essential			
YLR347C	***KAP95***	**2,684**	**0,000**	**3,905**	**0,088**	-3,631	0,001	0,750	0,037	Karyopherin beta, forms a dimeric complex with Srp1p (Kap60p) that mediates nuclear import of cargo proteins via a nuclear localization signal (NLS), interacts with nucleoporins to guide transport across the nuclear pore complex	nucleus	Verified	Essential			NT
YMR290C	***HAS1***	**5,005**	**0,003**	**3,870**	**0,038**	-5,450	0,003	-4,408	0,003	ATP-dependent RNA helicase; localizes to both the nuclear periphery and nucleolus; highly enriched in nuclear pore complex fractions; constituent of 66S pre-ribosomal particles	nucleolus	Verified	Essential			
YOR372C	***NDD1***	**3,899**	**0,001**	**3,858**	**0,021**	-5,057	0,000	-3,965	0,000	Transcriptional activator Essential for nuclear division; localized to the nucleus; Essential component of the mechanism that activates the expression of a set of late-S-phase-specific genes	nucleus	Verified	Essential			

YKL111C	***YKL111C***	4,471	0,008	3,857	0,091	-4,885	0,000	-3,588	0,000	Dubious open reading frame, unlikely to encode a protein; not conserved in closely related *Saccharomyces* species; partially overlaps the verified Essential gene ABF1	unknown	Dubious	Essential			
YGL226C-A	***OST5***	2,464	0,012	3,842	0,055	-0,577	0,113	0,186	0,758	Zeta subunit of the oligosaccharyltransferase complex of the ER lumen, which catalyzes asparagine-linked glycosylation of newly synthesized proteins	Endoplasmic Reticulum	Verified	Homodiploid			
YGR246C	***BRF1***	3,097	0,003	3,827	0,005	-4,523	0,001	-4,890	0,002	TFIIIB B-related factor, one of three subunits of RNA polymerase III transcription initiation factor TFIIIB, binds TFIIIC and TBP and recruits RNA pol III to promoters, amino-terminal half is homologous to TFIIB	nucleus	Verified	Essential			
YBR072W	***HSP26***	3,176	0,007	3,821	0,078	-4,319	0,000	-0,965	0,001	Small heat shock protein (sHSP) with chaperone activity; forms hollow, sphere-shaped oligomers that suppress unfolded proteins aggregation; oligomer activation requires a heat-induced conformational change; not expressed in unstressed cells	nucleus, cytoplasm	Verified	Homodiploid			
YNL131W	***TOM22***	5,754	0,069	3,769	0,094	-2,218	0,008	-2,879	0,010	Component of the TOM (translocase of outer membrane) complex responsible for initial import of mitochondrially directed proteins; acts as a receptor for precursor proteins and mediates interaction between the TOM and TIM complexes	mitochondrion	Verified	Essential			CCP
YDR485C	***VPS72***	2,133	0,030	3,686	0,088	-4,328	0,001	-0,835	0,069	Htz1p-binding component of the SWR1 complex, which exchanges histone variant H2AZ (Htz1p) for chromatin-bound histone H2A; required for vacuolar protein sorting	nucleus	Verified	Homodiploid	MR		ionic
YPL028W	***ERG10***	5,165	0,005	3,680	0,071	-1,497	0,137	-4,151	0,005	Acetyl-CoA C-acetyltransferase (acetoacetyl-CoA thiolase), cytosolic enzyme that transfers an acetyl group from one acetyl-CoA molecule to another, forming acetoacetyl-CoA; involved in the first step in mevalonate biosynthesis	cytoplasm	Verified	Essential			
YJL009W	***YJL009W***	4,454	0,099	3,650	0,031	-4,982	0,000	-4,118	0,004	Dubious open reading frame unlikely to encode a protein, based on available experimental and comparative sequence data; partially overlaps CCT8/YJL008C, a verified gene encoding a subunit of the cytosolic chaperonin Cct ring complex	unknown	Dubious	Essential			
YJL019W	***MPS3***	2,900	0,005	3,597	0,021	-3,899	0,001	-4,501	0,005	Nuclear envelope protein required for SPB duplication and nuclear fusion; localizes to the SPB half bridge and at telomeres during meiosis; required with Ndj1p and Csm4p for meiotic bouquet formation and telomere-led rapid prophase movement	nucleus, Spindle Pole Body, telomere	Verified	Essential			
YIR011C	***STS1***	2,727	0,008	3,589	0,063	-6,233	0,000	-4,033	0,000	Protein that interacts with the karyopherin Srp1p; may have a role with Srp1p in ubiquitin-mediated protein degradation	nucleus	Verified	Essential	CS		
YPL022W	***RAD1***	4,882	0,014	3,566	0,001	-5,507	0,011	-0,893	0,007	Single-stranded DNA endonuclease (with Rad10p), cleaves single-stranded DNA during nucleotide excision repair and double-strand break repair; subunit of Nucleotide Excision Repair Factor 1 (NEF1); homolog of human XPF protein	nucleus	Verified	Homodiploid	ALF, Can^R, MR, MF	MMS, UV, Cisplatin	
YIL101C	***XBP1***	2,631	0,047	3,552	0,047	-4,474	0,000	-0,163	0,252	Transcriptional repressor that binds to promoter sequences of the cyclin genes, CYS3, and SMF2; expression is induced by stress or starvation during mitosis, and late in meiosis; member of the Swi4p/Mbp1p family; potential Cdc28p substrate	nucleus	Verified	Homodiploid			
YAL028W	***FRT2***	3,522	0,007	3,548	0,009	-3,994	0,003	-3,382	0,008	Tail-anchored endoplasmic reticulum membrane protein, interacts with homolog Frt1p but is not a substrate of calcineurin (unlike Frt1p), promotes growth in conditions of high Na+, alkaline pH, or cell wall stress; potential Cdc28p substrate	Endoplasmic Reticulum	Verified	Homodiploid		UV	
YJR109C	***CPA2***	6,665	0,012	3,530	0,060	-5,424	0,000	-4,025	0,000	Large subunit of carbamoyl phosphate synthetase, which catalyzes a step in the synthesis of citrulline, an arginine precursor	carbamoyl-phosphate synthase complex	Verified	Homodiploid			

YPR018W	***RLF2***	**2,678**	**0,013**	**3,524**	**0,021**	-3,109	0,001	-1,253	0,002	Largest subunit (p90) of the Chromatin Assembly Complex (CAF-1) with Cac2p and Msi1p that assembles newly synthesized histones onto recently replicated DNA; involved in the maintenance of transcriptionally silent chromatin	nucleus	Verified	Homodiploid	LOH, CM	Camptothecin, UV	SA
YPL055C	***LGE1***	**3,297**	**0,071**	**3,520**	**0,014**	**2,894**	0,620	-0,010	1,000	Protein of unknown function; null mutant forms abnormally large cells, and homozygous diploid null mutant displays delayed premeiotic DNA synthesis and reduced efficiency of meiotic nuclear division	nucleus	Verified	Homodiploid	BIM, CTF, ALF, CM, CS, CSA	HU, UV, Doxorubicin	SA
YBR070C	***ALG14***	**2,032**	**0,031**	**3,512**	**0,046**	-1,530	0,102	-1,124	0,173	Component of UDP-GlcNAc transferase required for the second step of dolichyl-linked oligosaccharide synthesis; anchors the catalytic subunit Alg13p to the ER membrane; similar to bacterial and human glycosyltransferases	unknown	Verified	Essential			
YDR221W	***GTB1***	**2,935**	**0,002**	**3,456**	**0,025**	0,226	0,009	-2,337	0,000	Glucosidase II beta subunit, forms a complex with alpha subunit Rot2p, involved in removal of two glucose residues from N-linked glycans during glycoprotein biogenesis in the ER	Endoplasmic Reticulum	Verified	Homodiploid			
YLR044C	***PDC1***	**3,496**	**0,004**	**3,452**	**0,065**	-4,288	0,000	-2,904	0,000	Major of three pyruvate decarboxylase isozymes, key enzyme in alcoholic fermentation, decarboxylates pyruvate to acetaldehyde; subject to glucose-, ethanol-, and autoregulation; involved in amino acid catabolism	nucleus, cytoplasm	Verified	Homodiploid		OXY	
YPL238C	***YPL238C***	**3,089**	**0,036**	**3,440**	**0,021**	-5,048	0,020	-5,450	0,001	Dubious open reading frame unlikely to encode a functional protein, based on available experimental and comparative sequence data; partially overlaps 5' end of the verified Essential gene SUI3/YPL237W	unknown	Dubious	Essential			
YBR174C	***YBR174C***	**2,663**	**0,072**	**3,435**	**0,082**	-3,717	0,001	-2,623	0,000	Dubious open reading frame unlikely to encode a protein, based on available experimental and comparative sequence data; partially overlaps the verified ORF YBR175W; null mutant is viable and sporulation defective	unknown	Dubious	Homodiploid		HU, UV	
YGR156W	***PTI1***	**3,660**	**0,020**	**3,415**	**0,049**	-3,571	0,002	-3,784	0,001	Pta1p Interacting protein	nucleus	Verified	Essential			
YDR293C	***SSD1***	**4,841**	**0,000**	**3,406**	**0,004**	-4,083	0,000	-4,440	0,000	Protein with a role in maintenance of cellular integrity, interacts with components of the TOR pathway; ssd1 mutant of a clinical S. cerevisiae strain displays elevated virulence	cytoplasm	Verified	Homodiploid	CM		
YJL010C	***YJL010C***	**2,904**	**0,025**	**3,389**	**0,011**	-2,430	0,002	-5,469	0,005	Essential component of pre-40S ribosomes that is required for early cleavages of 35S pre-rRNA and hence formation of 18S rRNA; binds RNA in vitro and contains multiple pumilio-like repeats	nucleolus	Verified	Essential			
YNL272C	***SEC2***	**4,926**	**0,025**	**3,361**	**0,021**	-4,449	0,003	-4,329	0,001	Guanyl-nucleotide exchange factor for the small G-protein Sec4p, located on cytoplasmic vesicles; Essential for post-Golgi vesicle transport	Golgi apparatus, vesicles	Verified	Essential			
YBL097W	***BRN1***	**3,900**	**0,090**	**3,339**	**0,035**	-3,566	0,013	-1,252	0,008	Essential protein required for chromosome condensation, likely to function as an intrinsic component of the condensation machinery, may influence multiple aspects of chromosome transmission and dynamics	nucleus	Verified	Essential	CSA		SA, CCP
YNL222W	***SSU72***	**4,216**	**0,001**	**3,289**	**0,061**	-3,565	0,002	-3,342	0,001	Transcription/RNA-processing factor that associates with TFIIB and cleavage/polyadenylation factor Pta1p; exhibits phosphatase activity on serine-5 of the RNA polymerase II C-terminal domain; affects start site selection in vivo	nucleolus	Verified	Essential			
YPL029W	***SUV3***	**2,431**	**0,046**	**3,269**	**0,009**	-2,937	0,000	-1,667	0,005	ATP-dependent RNA helicase, component of the mitochondrial degradosome along with the RNase Dss1p; the degradosome associates with the ribosome and mediates turnover of aberrant or unprocessed RNAs	mitochondrion	Verified	Homodiploid	rho-MF	Doxorubicin	
YBR252W	***DUT1***	**2,589**	**0,007**	**3,234**	**0,013**	-4,931	0,002	-2,375	0,033	dUTPase, catalyzes hydrolysis of dUTP to dUMP and PPi, thereby preventing incorporation of uracil into DNA during replication; critical for the maintenance of genetic stability	nucleus	Verified	Essential	MF		

YAR035W	***YAT1***	**2,551**	**0,007**	**3,138**	**0,015**	0,549	0,167	-1,036	0,036	Outer mitochondrial carnitine acetyltransferase, minor ethanol-inducible enzyme involved in transport of activated acyl groups from the cytoplasm into the mitochondrial matrix; phosphorylated	mitochondrion	Verified	Homodiploid		UV	
YOR122C	***PFY1***	**2,064**	**0,010**	**3,119**	**0,070**	-4,512	0,000	-2,207	0,000	Profilin, actin- and phosphatidylinositol 4,5-bisphosphate-binding protein, involved in cytoskeleton organization, required for normal timing of actin polymerization in response to thermal stress; localizes to plasma membrane and cytosol	cytoplasm, plasma membrane	Verified	Essential			
YER018C	***SPC25***	**5,749**	**0,082**	**3,116**	**0,055**	-2,878	0,002	-1,804	0,302	Component of the evolutionarily conserved kinetochore-associated Ndc80 complex (Ndc80p-Nuf2p-Spc24p-Spc25p); involved in chromosome segregation, spindle checkpoint activity and kinetochore clustering	nucleus	Verified	Essential			
YOR143C	***THI80***	**2,538**	**0,087**	**3,111**	**0,057**	-4,597	0,000	-4,381	0,001	Thiamine pyrophosphokinase, phosphorylates thiamine to produce the coenzyme thiamine pyrophosphate (thiamine diphosphate)	cytoplasm	Verified	Essential			
YAL036C	***RBG1***	**2,079**	**0,000**	**3,049**	**0,002**	-4,151	0,006	-4,614	0,002	Member of the DRG family of GTP-binding proteins; interacts with translating ribosomes and with Tma46p	cytoplasm	Verified	Homodiploid			
YAL065C	***YAL065C***	**2,221**	**0,002**	**3,041**	**0,002**	-4,295	0,000	-1,888	0,009	Putative protein of unknown function; has homology to FLO1; possible pseudogene	unknown	Uncharacterized	Homodiploid		Tirapazamine	
YKL008C	***LAC1***	**2,700**	**0,001**	**3,029**	**0,025**	-4,456	0,000	-1,431	0,000	Ceramide synthase component, involved in synthesis of ceramide from C26(acyl)-coenzyme A and dihydrosphingosine or phytosphingosine, functionally equivalent to Lag1p	Endoplasmic Reticulum	Verified	Homodiploid			
YMR185W	***YMR185W***	**4,990**	**0,000**	**3,002**	**0,035**	-4,694	0,001	-5,973	0,001	Putative protein of unknown function; Essential gene required for viability	unknown	Uncharacterized	Essential			
YBR032W	***YBR032W***	**3,000**	**0,002**	**2,991**	**0,048**	-5,040	0,000	-5,651	0,000	Dubious open reading frame unlikely to encode a protein, based on experimental and comparative sequence data	unknown	Dubious	Homodiploid			Benomyl
YLL031C	***GPI13***	**4,086**	**0,079**	**2,973**	**0,039**	-3,228	0,000	-2,460	0,003	ER membrane localized phosphoryltransferase that adds phosphoethanolamine onto the third mannose residue of the glycosylphosphatidylinositol (GPI) anchor precursor; similar to human PIG-O protein	Endoplasmic Reticulum	Verified	Essential			
YGR204W	***ADE3***	**2,247**	**0,007**	**2,956**	**0,039**	-2,245	0,000	-2,694	0,001	Cytoplasmic trifunctional enzyme C1-tetrahydrofolate synthase, involved in single carbon metabolism and required for biosynthesis of purines, thymidylate, methionine, and histidine	cytoplasm	Verified	Homodiploid	HAP	Doxorubicin	
YOR098C	***NUP1***	**3,462**	**0,001**	**2,942**	**0,007**	-4,155	0,004	-3,512	0,023	Nuclear pore complex (NPC) subunit, involved in protein import/export and in export of RNAs, possible karyopherin release factor that accelerates release of karyopherin-cargo complexes after transport across NPC; potential Cdc28p substrate	nucleus	Verified	Essential			NT
YDR139C	***RUB1***	**2,067**	**0,002**	**2,895**	**0,090**	-5,428	0,000	-4,259	0,000	Ubiquitin-like protein with similarity to mammalian NEDD8; conjugation (neddylation) substrates include the cullins Cdc53p, Rtt101p, and Cul3p; activated by Ula1p and Uba3p (E1 enzyme pair); conjugation mediated by Ubc12p (E2 enzyme)	cytoplasm	Verified	Homodiploid			
YDR370C	***YDR370C***	**3,180**	**0,001**	**2,870**	**0,044**	-6,054	0,000	-0,091	0,580	Putative protein of unknown function	unknown	Uncharacterized	Homodiploid			
YNL182C	***IPI3***	**2,087**	**0,036**	**2,839**	**0,037**	-4,871	0,000	-4,304	0,000	Essential component of the Rix1 complex (Rix1p, Ipi1p, Ipi3p) that is required for processing of ITS2 sequences from 35S pre-rRNA; highly conserved and contains WD40 motifs; Rix1 complex associates with Mdn1p in pre-60S ribosomal particles	nucleolus	Verified	Essential			
YDL143W	***CCT4***	**3,466**	**0,042**	**2,819**	**0,049**	-5,264	0,000	-2,886	0,000	Subunit of the cytosolic chaperonin Cct ring complex, related to Tcp1p, required for the assembly of actin and tubulins in vivo	T-complex	Verified	Essential			
YIL083C	***YIL083C***	**2,374**	**0,086**	**2,811**	**0,029**	-3,206	0,002	-3,964	0,003	Putative phosphopantothenoylcysteine synthetase (PPCS) that catalyzes the second step in the biosynthesis of coenzyme A from pantothenate; conserved in bacteria, humans, and plants; Essential for viability	nucleus, cytoplasm	Uncharacterized	Essential			

YIR002C	MPH1	5,464	0,050	2,800	0,001	-4,243	0,000	-1,523	0,000	Member of the DEAH family of helicases, functions in an error-free DNA damage bypass pathway that involves homologous recombination, mutations confer a mutator phenotype	nucleus	Verified	Homodiploid	BIM	MMS	
YBR116C	YBR116C	2,215	0,000	2,792	0,077	-3,977	0,000	-2,885	0,000	Dubious open reading frame unlikely to encode a protein, based on available experimental and comparative sequence data; partially overlaps the verified gene TKL2	unknown	Dubious	Homodiploid			
YPL078C	ATP4	2,194	0,014	2,722	0,015	-3,381	0,000	-1,373	0,000	Subunit b of the stator stalk of mitochondrial F1F0 ATP synthase, which is a large, evolutionarily conserved enzyme complex required for ATP synthesis; phosphorylated	mitochondrion	Verified	Homodiploid			CCP
YKL034W	TUL1	3,469	0,021	2,696	0,027	0,996	0,000	-0,488	0,110	Golgi-localized RING-finger ubiquitin ligase (E3), involved in ubiquitinating and sorting membrane proteins that contain polar transmembrane domains to multivesicular bodies for delivery to the vacuole for quality control purposes	Golgi apparatus	Verified	Homodiploid			
YGR171C	MSM1	2,121	0,077	2,667	0,040	0,478	0,008	-2,221	0,000	Mitochondrial methionyl-tRNA synthetase (MetRS), functions as a monomer in mitochondrial protein synthesis; functions similarly to cytoplasmic MetRS although the cytoplasmic form contains a zinc-binding domain not found in Msm1p	mitochondrion	Verified	Homodiploid	ALF	Doxorubicin	
YGL007W	BRP1	2,580	0,089	2,655	0,024	-2,092	0,060	-1,831	0,207	Dubious ORF located in the upstream region of PMA1, deletion leads to polyamine resistance due to downregulation of PMA1	unknown	Dubious	Homodiploid			
YJL148W	RPA34	4,447	0,007	2,628	0,061	-3,278	0,000	-2,581	0,000	RNA polymerase I subunit A34.5	nucleolus	Verified	Homodiploid	LOH, CM, CS	Doxorubicin	
YDL076C	RXT3	2,245	0,071	2,618	0,043	-5,193	0,000	0,697	0,028	Subunit of the RPD3L complex; involved in histone deacetylation	nucleus	Verified	Homodiploid			
YBR126C	TPS1	5,447	0,086	2,618	0,033	**2,434**	0,158	-0,353	0,644	Synthase subunit of trehalose-6-phosphate synthase/phosphatase complex, which synthesizes the storage carbohydrate trehalose; also found in a monomeric form; expression is induced by the stress response and repressed by the Ras-cAMP pathway	cytoplasm	Verified	Homodiploid		Doxorubicin	
YBR124W	YBR124W	4,217	0,024	2,612	0,067	-2,434	0,000	-3,893	0,000	Putative protein of unknown function	unknown	Dubious	Essential			
YLR371W	ROM2	3,305	0,015	2,605	0,050	-1,985	0,103	-3,281	0,004	GDP/GTP exchange protein (GEP) for Rho1p and Rho2p; mutations are synthetically lethal with mutations in rom1, which also encodes a GEP	bud tip	Verified	Homodiploid			Benomyl
YGL260W	YGL260W	2,255	0,000	2,593	0,020	-5,306	0,000	0,449	0,034	Putative protein of unknown function; transcription is significantly increased in a NAP1 deletion background; deletion mutant has increased accumulation of nickel and selenium	unknown	Uncharacterized	Homodiploid			
YBR233W	PBP2	3,102	0,023	2,535	0,078	-4,472	0,000	-4,222	0,000	RNA binding protein with similarity to mammalian heterogeneous nuclear RNP K protein, involved in the regulation of telomere position effect and telomere length	nucleus	Verified	Homodiploid			
YDL062W	YDL062W	2,293	0,001	2,503	0,008	-4,236	0,000	-2,774	0,003	Dubious open reading frame unlikely to encode a protein, based on available experimental and comparative sequence data; partially overlaps uncharacterized ORF YDL063C; YDL062W is not Essential	unknown	Dubious	Homodiploid			
YMR298W	LIP1	2,657	0,007	2,496	0,013	-5,286	0,000	-5,216	0,000	Ceramide synthase subunit; single-span ER membrane protein associated with Lag1p and Lac1p and required for ceramide synthase activity, null mutant grows extremely slowly and is defective in ceramide synthesis	Endoplasmic Reticulum	Verified	Essential			
YLR336C	SGD1	2,190	0,045	2,495	0,021	-1,948	0,027	-0,999	0,581	Essential nuclear protein with a possible role in the osmoregulatory glycerol response; interacts with phospholipase C (Plc1p); putative homolog of human NOM1 which is implicated in acute myeloid leukemia	nucleus	Verified	Essential			NT
YNL143C	YNL143C	2,716	0,001	2,492	0,042	-5,116	0,000	-2,657	0,000	Dubious open reading frame unlikely to encode a protein, based on available experimental and comparative sequence data	unknown	Dubious	Homodiploid			
YIL055C	YIL055C	2,600	0,020	2,472	0,030	-4,365	0,000	-5,397	0,000	Putative protein of unknown function	unknown	Uncharacterized	Homodiploid	RMCD		

YLR397C	***AFG2***	**2,659**	**0,027**	**2,467**	**0,018**	-1,486	0,207	-1,560	0,020	ATPase of the CDC48/PAS1/SEC18 (AAA) family, forms a hexameric complex; may be involved in degradation of aberrant mRNAs	intracellular	Verified	Essential			NT
YDR211W	***GCD6***	**2,333**	**0,018**	**2,463**	**0,056**	-4,128	0,023	-4,001	0,017	Catalytic epsilon subunit of the translation initiation factor eIF2B, the guanine-nucleotide exchange factor for eIF2; activity subsequently regulated by phosphorylated eIF2; first identified as a negative regulator of GCN4 expression	nucleus	Verified	Essential			
YBL018C	***POP8***	**4,801**	**0,000**	**2,418**	**0,019**	-4,101	0,000	-3,384	0,000	Subunit of both RNase MRP, which cleaves pre-rRNA, and nuclear RNase P, which cleaves tRNA precursors to generate mature 5' ends	nucleolus	Verified	Essential			
YKL022C	***CDC16***	**2,846**	**0,011**	**2,396**	**0,006**	-3,500	0,004	-3,399	0,001	Subunit of the anaphase-promoting complex/cyclosome (APC/C), which is a ubiquitin-protein ligase required for degradation of anaphase inhibitors, including mitotic cyclins, during the metaphase/anaphase transition; required for sporulation	nucleus	Verified	Essential	TL		CCP
YBR133C	***HSL7***	**2,041**	**0,096**	**2,396**	**0,076**	-2,954	0,016	-0,637	0,326	Protein arginine N-methyltransferase that exhibits septin and Hsl1p-dependent bud neck localization and periodic Hsl1p-dependent phosphorylation; required along with Hsl1p for bud neck recruitment, phosphorylation, and degradation of Swe1p	bud neck	Verified	Homodiploid		HU, Camptothecin	
YIL091C	***YIL091C***	**3,739**	**0,015**	**2,341**	**0,046**	-2,306	0,029	-2,664	0,002	Nucleolar protein of unknown function; proposed to function as an RNA helicase based on structure prediction and remote homology searches; Essential for viability	nuclolus	Uncharacterized	Essential			
YBR031W	***RPL4A***	**5,104**	**0,027**	**2,340**	**0,077**	-3,843	0,007	-0,970	0,005	N-terminally acetylated protein component of the large (60S) ribosomal subunit, nearly identical to Rpl4Bp and has similarity to E. coli L4 and rat L4 ribosomal proteins	Endoplasmic Reticulum, cytoplasm	Verified	Homodiploid			CCP
YDR290W	***YDR290W***	**3,681**	**0,055**	**2,340**	**0,012**	-4,289	0,000	-2,076	0,238	Dubious open reading frame unlikely to encode a protein, based on available experimental and comparative sequence data; partially overlaps the verified ORF RTT103	unknown	Dubious	Homodiploid	CTF, ALF, CS, TE-T		
YDR283C	***GCN2***	**2,644**	**0,070**	**2,339**	**0,099**	-4,297	0,000	0,250	0,084	Protein kinase, phosphorylates the alpha-subunit of translation initiation factor eIF2 (Sui2p) in response to starvation; activated by uncharged tRNAs and the Gcn1p-Gcn20p complex	cytoplasm	Verified	Homodiploid			
YMR206W	***YMR206W***	**2,138**	**0,004**	**2,300**	**0,074**	-4,842	0,000	-5,187	0,000	Putative protein of unknown function; YMR206W is not an Essential gene	unknown	Uncharacterized	Homodiploid			
YJL174W	***KRE9***	**2,557**	**0,077**	**2,254**	**0,084**	-4,358	0,002	-4,594	0,000	Glycoprotein involved in cell wall beta-glucan assembly; null mutation leads to severe growth defects, aberrant multibudded morphology, and mating defects	extracellular region	Verified	Essential			
YNL086W	***YNL086W***	**2,068**	**0,002**	**2,226**	**0,074**	-5,899	0,000	-3,027	0,000	Putative protein of unknown function; green fluorescent protein (GFP)-fusion protein localizes to endosomes	endosome	Uncharacterized	Homodiploid	ALF		
YBR014C	***YBR014C***	**5,785**	**0,049**	**2,212**	**0,090**	-4,085	0,000	-3,609	0,000	Cis-golgi localized monothiol glutaredoxin; more similar in activity to dithiol than other monothiol glutaredoxins; involved in the oxidative stress response; does not bind metal ions; functional overlap with GRX6	Golgi apparatus	Verified	Homodiploid			
YPR189W	***SKI3***	**2,625**	**0,031**	**2,190**	**0,009**	-3,661	0,000	-1,880	0,005	Protein involved in exosome mediated 3' to 5' mRNA degradation and translation inhibition of non-poly(A) mRNAs; forms complex with Ski2p and Ski8p; required for repressing propagation of dsRNA viruses	cytoplasm	Verified	Homodiploid			
YJL190C	***RPS22A***	**3,241**	**0,001**	**2,182**	**0,041**	-6,099	0,000	0,053	0,706	Protein component of the small (40S) ribosomal subunit; nearly identical to Rps22Bp and has similarity to E. coli S8 and rat S15a ribosomal proteins	cytoplasm	Verified	Homodiploid	TE-T, TL		Benomyl
YBR278W	***DPB3***	**2,286**	**0,000**	**2,158**	**0,053**	-6,370	0,000	-0,174	0,568	Third-largest subunit of DNA polymerase II (DNA polymerase epsilon), required to maintain fidelity of chromosomal replication and also for inheritance of telomeric silencing; mRNA abundance peaks at the G1/S boundary of the cell cycle	nucleus	Verified	Homodiploid	LOH, CM		SA

YML009C	***MRPL39***	**2,687**	**0,012**	**2,153**	**0,070**	-3,722	0,000	-0,291	0,106	Mitochondrial ribosomal protein of the large subunit	mitochondrion	Verified	Homodiploid	ALF		
YOL163W	***YOL163W***	**2,383**	**0,001**	**2,127**	**0,021**	-0,129	0,351	-0,535	0,020	Putative protein of unknown function; member of the Dal5p subfamily of the major facilitator family	membrane	Uncharacterized	Homodiploid		Tirapazamine	
YGR119C	***NUP57***	**2,137**	**0,021**	**2,107**	**0,046**	-3,122	0,000	-1,929	0,000	Nucleoporin, Essential subunit of the nuclear pore complex (NPC), functions as the organizing center of an NPC subcomplex containing Nsp1p, Nup49p, Nup57p, and Nic96p	nucleus	Verified	Essential			
YMR161W	***HLJ1***	**2,928**	**0,011**	**2,032**	**0,066**	-3,460	0,000	-3,759	0,001	Co-chaperone for Hsp40p, anchored in the ER membrane; with its homolog Hdj1p promotes ER-associated protein degradation (ERAD) of integral membrane substrates; similar to E. coli DnaJ	Endoplasmic Reticulum	Verified	Homodiploid			

Strains with elevated SLM at CAN1 locus which mate with MATα or MATa, but slight if any SLM at URA3 locus, might be haploid

{[(LogRatio SLM CAN1 > 2) AND [(LogRatio cross with MATα > 1) OR (LogRatio cross with MATa > 1)] AND [LogRatio SLM URA3 < 2]}

ORF	***Name***	**SLM at *CAN1* locus**		**SLM at *URA3* locus**		**cross with *MATalpha***		**cross with *MATa***		**Description**	**Localization**	**Status**	**Collection**	**Instability phenotypes**	**Genotoxic stress sensitivity**	**Other genome related phenotypes**
		**LogR**	**p-val**	**LogR**	**p-val**	**LogR**	**p-val**	**LogR**	**p-val**							
YHR191C	***CTF8***	**13,475**	**0,000**	**-1,079**	**0,749**	4,959	0,002	**-3,360**	0,001	Subunit of a complex with Ctf18p that shares some subunits with Replication Factor C and is required for sister chromatid cohesion	nucleus	Verified	Homodiploid	ALF, CM, CS, CSA, TL	HU, MMS, Camptothecin, Bleomycin A2, Cisplatin,	SPO*, Benomyl
YBR084W	***MIS1***	**12,917**	**0,000**	**1,421**	**0,066**	**5,435**	0,000	-3,569	0,000	Mitochondrial C1-tetrahydrofolate synthase, involved in interconversion between different oxidation states of tetrahydrofolate (THF); provides activities of formyl-THF synthetase, methenyl-THF cyclohydrolase, and methylene-THF dehydrogenase	mitochondrion	Verified	Homodiploid			
YOR008C	***SLG1***	**11,934**	**0,000**	**-0,905**	**0,077**	**3,710**	0,000	-4,594	0,000	Sensor-transducer of the stress-activated PKC1-MPK1 kinase pathway involved in maintenance of cell wall integrity; involved in organization of the actin cytoskeleton; secretory pathway Wsc1p is required for the arrest of secretion response	bud neck, plasma membrane	Verified	Homodiploid	RMCD	MMS	CCP
YPL262W	***FUM1***	**8,833**	**0,000**	**0,274**	**0,070**	-3,781	0,000	**4,853**	0,001	Fumarase, converts fumaric acid to L-malic acid in the TCA cycle; cytosolic and mitochondrial localization determined by the N-terminal mitochondrial targeting sequence and protein conformation; phosphorylated in mitochondria	mitochondrion, cytoplasm	Verified	Homodiploid	RMCD		
YKL029C	***MAE1***	**8,763**	**0,002**	**1,448**	**0,075**	-3,919	0,000	**2,607**	0,015	Mitochondrial malic enzyme, catalyzes the oxidative decarboxylation of malate to pyruvate, which is a key intermediate in sugar metabolism and a precursor for synthesis of several amino acids	mitochondrion	Verified	Homodiploid			
YJR033C	***RAV1***	**7,792**	**0,000**	**1,751**	**0,097**	**4,951**	0,000	-3,034	0,000	Subunit of the RAVE complex (Rav1p, Rav2p, Skp1p), which promotes assembly of the V-ATPase holoenzyme; required for transport between the early and late endosome/PVC and for localization of TGN membrane proteins; potential Cdc28p substrate	vacuole	Verified	Homodiploid	RMCD	Doxorubicin, Metal	
YJR031C	***GEA1***	**6,209**	**0,003**	**0,169**	**0,026**	**4,171**	0,000	-1,743	0,000	Guanine nucleotide exchange factor for ADP ribosylation factors (ARFs), involved in vesicular transport between the Golgi and ER, Golgi organization, and actin cytoskeleton organization; similar to but not functionally redundant with Gea2p	Golgi apparatus, Endoplasmic Reticulum, actin cytoskeleton	Verified	Homodiploid			
YDR247W	***VHS1***	**6,020**	**0,004**	**1,349**	**0,045**	-3,165	0,000	**3,125**	0,009	Cytoplasmic serine/threonine protein kinase; identified as a high-copy suppressor of the synthetic lethality of a sis2 sit4 double mutant, suggesting a role in G1/S phase progression; homolog of Sks1p	cytoplasm	Verified	Homodiploid			

YKR020W	VPS51	**3,437**	**0,054**	**-0,256**	**0,088**	**3,912**	0,000	-2,335	0,002	Component of the GARP (Golgi-associated retrograde protein) complex, Vps51p-Vps52p-Vps53p-Vps54p, which is required for the recycling of proteins from endosomes to the late Golgi; links the (VFT/GARP) complex to the SNARE Tlg1p	Golgi apparatus	Verified	Homodiploid	RMCD	Metal	SPO, ionic
YBR236C	ABD1	**3,159**	**0,017**	**1,544**	**0,078**	**1,798**	0,000	-4,204	0,000	Methyltransferase, catalyzes the transfer of a methyl group from S-adenosylmethionine to the GpppN terminus of capped mRNA	nucleus	Verified	Essential			
YNR018W	YNR018W	**2,601**	**0,036**	**-0,658**	**0,028**	1,056	0,008	-0,571	0,008	Putative protein of unknown function; non-tagged protein is detected in purified mitochondria; null mutant displays decreased frequency of mitochondrial genome loss (petite formation) and severe growth defect in minimal glycerol media	mitochondrion	Uncharacterized	Homodiploid			
YAL022C	FUN26	**2,337**	**0,001**	**-1,036**	**0,065**	**2,047**	0,016	-4,441	0,013	Nucleoside transporter with broad nucleoside selectivity; localized to intracellular membranes	intracellular membranes	Verified	Homodiploid		UVerified	
YDR466W	PKH3	**2,325**	**0,003**	**0,569**	**0,048**	1,175	0,000	-3,828	0,000	Protein kinase with similarity to mammalian phosphoinositide-dependent kinase 1 (PDK1) and yeast Pkh1p and Pkh2p, two redundant upstream activators of Pkc1p; identified as a multicopy suppressor of a pkh1 pkh2 double mutant	unknown	Verified	Homodiploid			
YBR107C	IML3	**2,307**	**0,007**	**0,089**	**0,049**	-3,289	0,000	1,295	0,005	Protein with a role in kinetochore function, localizes to the outer kinetochore in a Ctf19p-dependent manner, interacts with Chl4p and Ctf19p	nucleus	Verified	Homodiploid	BIM, CTF, ALF, CS, CSA		
YML036W	CGI121	**2,238**	**0,067**	**1,907**	**0,006**	-1,934	0,035	**2,539**	0,029	Protein involved in telomere uncapping and elongation as component of the KEOPS protein complex with Bud32p, Kae1p, Pcc1p, and Gon7p; also shown to be a component of the EKC protein complex; homolog of human CGI-121	nucleus	Verified	Homodiploid			
YGR078C	PAC10	**2,213**	**0,060**	**0,358**	**0,013**	1,284	0,001	-3,733	0,002	Part of the heteromeric co-chaperone GimC/prefoldin complex, which promotes efficient protein folding	cytoplasm	Verified	Homodiploid	BIM, ALF, CM, CSA, MR	HU	Benomyl
YOL079W	YOL079W	**2,040**	**0,003**	**0,195**	**0,086**	-4,010	0,000	**1,570**	0,000	Dubious open reading frame unlikely to encode a functional protein, based on available experimental and comparative sequence data	unknown	Dubious	Homodiploid			

Strains with elevated SLM at URA3 locus which mate with MATα or MATa, but slight if any SLM at CAN1 locus

{[(LogRatio SLM URA3 > 2) AND [(LogRatio cross with MATα > 1) OR (LogRatio cross with MATa > 1)] AND [LogRatio SLM CAN1 < 2]}

ORF	Name	SLM at *CAN1* locus		SLM at *URA3* locus		cross with *MATα*		cross with *MATa*		Description	Localization	Status	Collection	Instability phenotypes	Genotoxic stress sensitivity	Other genome related phenotypes
		LogR	p-val	LogR	p-val	LogR	p-val	LogR	p-val							
YML086C	ALO1	**0,802**	**0,002**	**6,329**	**0,021**	-4,198	0,000	1,075	0,002	D-Arabinono-1,4-lactone oxidase, catalyzes the final step in biosynthesis of D-erythroascorbic acid, which is protective against oxidative stress	mitochondrion	Verified	Homodiploid	GCR	OXY	CCP
YML126C	ERG13	**1,151**	**0,016**	**5,564**	**0,046**	1,142	0,000	-1,538	0,001	3-hydroxy-3-methylglutaryl-CoA (HMG-CoA) synthase, catalyzes the formation of HMG-CoA from acetyl-CoA and acetoacetyl-CoA; involved in the second step in mevalonate biosynthesis	Endoplasmic Reticulum	Verified	Essential			
YIR029W	DAL2	**-0,071**	**0,000**	**5,546**	**0,045**	1,028	0,004	-0,819	0,014	Allantoicase, converts allantoate to urea and ureidoglycolate in the second step of allantoin degradation; expression sensitive to nitrogen catabolite repression and induced by allophanate, an intermediate in allantoin degradation	unknown	Verified	Homodiploid			
YJL016W	YJL016W	**0,842**	**0,013**	**5,074**	**0,002**	-5,780	0,002	**2,034**	0,001	Putative protein of unknown function; GFP-fusion protein localizes to the cytoplasm; conserved in closely related *Saccharomyces* species	cytoplasm	Uncharacterized	Homodiploid			
YIL057C	YIL057C	**-0,240**	**0,010**	**5,049**	**0,017**	**1,649**	0,000	-0,937	0,011	Putative protein of unknown function; expression induced under carbon limitation and repressed under high glucose	unknown	Uncharacterized	Homodiploid			

YDR209C	YDR209C	-0,349	0,001	4,854	0,034	-5,481	0,000	1,368	0,020	Dubious open reading frame unlikely to encode a protein, based on available experimental and comparative sequence data; partially overlaps uncharacterized gene YDR210W.	unknown	Dubious	Homodiploid			
YGR081C	SLX9	1,239	0,006	4,238	0,087	2,385	0,000	-0,942	0,047	Protein required for pre-rRNA processing; associated with the 90S pre-ribosome and 43S small ribosomal subunit precursor; interacts with U3 snoRNA; deletion mutant has synthetic fitness defect with an sgs1 deletion mutant	nucleolus	Verified	Homodiploid			NT
YBL067C	UBP13	1,054	0,015	4,069	0,042	3,493	0,000	2,648	0,000	Putative ubiquitin carboxyl-terminal hydrolase, ubiquitin-specific protease that cleaves ubiquitin-protein fusions	unknown	Verified	Homodiploid			
YLR390W-A	CCW14	0,317	0,006	3,865	0,057	1,798	0,000	-0,535	0,052	Covalently linked cell wall glycoprotein, present in the inner layer of the cell wall	cell wall	Verified	Homodiploid	TL		
YJR034W	PET191	1,204	0,017	3,852	0,084	1,472	0,004	-3,139	0,016	Protein required for assembly of cytochrome c oxidase; exists as an oligomer that is integral to the mitochondrial inner membrane and faces the intermembrane space; contains a twin Cx9C motif	mitochondrion	Verified	Homodiploid	RMCD		
YOR139C	YOR139C	0,279	0,025	3,848	0,000	-2,795	0,005	2,585	0,013	Dubious open reading frame unlikely to encode a protein, based on available experimental and comparative sequence data; partially overlaps the verified ORF SFL1/YOR140W	unknown	Dubious	Homodiploid			Benomyl
YDR237W	MRPL7	0,729	0,073	3,839	0,032	1,561	0,000	-0,759	0,057	Mitochondrial ribosomal protein of the large subunit	mitochondrion	Verified	Homodiploid		MMS	
YIL023C	YKE4	-0,740	0,004	3,731	0,020	1,340	0,000	0,296	0,050	Zinc transporter; localizes to the ER; null mutant is sensitive to calcofluor white, leads to zinc accumulation in cytosol; ortholog of the mouse KE4 and member of the ZIP (ZRT, IRT-like Protein) family	Endoplasmic Reticulum	Verified	Homodiploid			
YPR083W	MDM36	0,317	0,002	3,651	0,097	-3,430	0,000	1,589	0,000	Protein required for normal mitochondrial morphology and inheritance	unknown	Verified	Homodiploid	RMCD		
YLR193C	UPS1	1,014	0,027	3,636	0,053	5,847	0,000	5,505	0,000	Mitochondrial intermembrane space protein that regulates alternative processing and sorting of Mgm1p and other proteins; required for normal mitochondrial morphology; ortholog of human PRELI	mitochondrion	Verified	Homodiploid			
YOR083W	WHI5	-0,129	0,001	3,547	0,023	2,657	0,000	-3,938	0,000	Repressor of G1 transcription that binds to SCB binding factor (SBF) at SCB target promoters in early G1; phosphorylation of Whi5p by the CDK, Cln3p/Cdc28p relieves repression and promoter binding by Whi5; periodically expressed in G1	nucleus	Verified	Homodiploid	CM	UVerified	
YDR175C	RSM24	1,894	0,050	3,419	0,029	1,816	0,000	-2,191	0,001	Mitochondrial ribosomal protein of the small subunit	mitochondrion	Verified	Homodiploid	mtGM		Benomyl
YBR251W	MRPS5	-2,157	0,059	3,409	0,032	1,928	0,000	-1,820	0,000	Mitochondrial ribosomal protein of the small subunit	mitochondrion	Verified	Homodiploid			
YJR016C	ILV3	-0,578	0,025	3,393	0,014	1,219	0,064	-3,364	0,017	Dihydroxyacid dehydratase, catalyzes third step in the common pathway leading to biosynthesis of branched-chain amino acids	mitochondrion	Verified	Essential			
YML131W	YML131W	-0,237	0,004	3,356	0,058	-4,344	0,000	2,116	0,000	Putative protein of unknown function with similarity to medium chain dehydrogenase/reductases; expression induced by stresses including osmotic shock, DNA damaging agents, and other chemicals; GFP-fusion protein localizes to the cytoplasm	cytoplasm	Uncharacterized	Homodiploid			
YER067C-A	YER067C-A	0,968	0,001	3,283	0,013	1,072	0,000	-1,004	0,005	Dubious open reading frame unlikely to encode a protein, based on available experimental and comparative sequence data; partially overlaps the uncharacterized ORF YER067W	unknown	Dubious	Homodiploid			
YDR462W	MRPL28	0,311	0,089	3,228	0,037	1,812	0,015	-1,749	0,017	Mitochondrial ribosomal protein of the large subunit	mitochondrion	Verified	Homodiploid			Benomyl
YFL027C	GYP8	-0,868	0,001	3,170	0,009	-3,446	0,000	2,203	0,000	GTPase-activating protein for yeast Rab family members; Ypt1p is the preferred in vitro substrate but also acts on Sec4p, Ypt31p and Ypt32p; involved in the regulation of ER to Golgi vesicle transport	Endoplasmic Reticulum, Golgi apparatus	Verified	Homodiploid			

YPR179C	HDA3	-0,177	0,000	3,157	0,002	1,592	0,000	-4,925	0,000	Subunit of a possibly tetrameric trichostatin A-sensitive class II histone deacetylase complex that contains an Hda1p homodimer and an Hda2p-Hda3p heterodimer; required for the activity of the complex; has similarity to Hda2p	nucleus	Verified	Homodiploid			SA
YBL091C-A	SCS22	0,119	0,000	3,134	0,041	1,278	0,000	-5,210	0,000	Protein involved in regulation of phospholipid metabolism; homolog of Scs2p; similar to D. melanogaster inturned protein	unknown	Verified	Homodiploid			
YBL038W	MRPL16	1,880	0,092	3,098	0,016	-1,947	0,000	2,016	0,000	Mitochondrial ribosomal protein of the large subunit	mitochondrion	Verified	Homodiploid		UVerified	
YML090W	YML090W	-0,103	0,049	3,054	0,008	4,814	0,000	-4,042	0,000	Dubious open reading frame unlikely to encode a protein, based on available experimental and comparative sequence data; partially overlaps the dubious ORF YML089C; exhibits growth defect on a non-fermentable (respiratory) carbon source	unknown	Dubious	Homodiploid	ALF		
YHR034C	PIH1	-1,704	0,044	3,038	0,001	4,772	0,000	-1,781	0,013	Protein of unresolved function; may function in protein folding and/or rRNA processing, interacts with a chaperone (Hsp82p), two chromatin remodeling factors (Rvb1p, Rvb2p) and two rRNA processing factors (Rrp43p, Nop58p)	nucleus, cytoplasm	Verified	Homodiploid			
YJR053W	BFA1	1,579	0,005	3,020	0,056	2,773	0,000	-2,402	0,000	Component of the GTPase-activating Bfa1p-Bub2p complex involved in multiple cell cycle checkpoint pathways that control exit from mitosis	Spindle Pole Body	Verified	Homodiploid	ALF		Benomyl, CCP
YAR047C	YAR047C	1,647	0,001	3,007	0,005	3,219	0,002	-1,224	0,028	Dubious open reading frame unlikely to encode a protein, based on available experimental and comparative sequence data	unknown	Dubious	Homodiploid			
YLR414C	YLR414C	-0,001	0,058	3,004	0,048	-4,964	0,000	2,092	0,000	Putative protein of unknown function; green fluorescent protein (GFP)-fusion protein localizes to the bud and cytoplasm; Hog1p is required for transcriptional induction in response to cell wall damage; YLR414C is not an Essential gene	bud, cytoplasm	Uncharacterized	Homodiploid			
YMR072W	ABF2	1,395	0,084	2,982	0,009	1,017	0,000	-1,085	0,003	Mitochondrial DNA-binding protein involved in mitochondrial DNA replication and recombination, member of HMG1 DNA-binding protein family; activity may be regulated by protein kinase A phosphorylation	mitochondrion	Verified	Homodiploid	mtGM		CCP
YNL111C	CYB5	0,858	0,022	2,982	0,035	-2,622	0,006	3,763	0,001	Cytochrome b5, involved in the sterol and lipid biosynthesis pathways; acts as an electron donor to support sterol C5-6 desaturation	Endoplasmic Reticulum, microsome	Verified	Homodiploid		HU	
YMR228W	MTF1	-0,226	0,001	2,973	0,035	1,638	0,000	-5,192	0,000	Mitochondrial RNA polymerase specificity factor with structural similarity to S-adenosylmethionine-dependent methyltransferases and functional similarity to bacterial sigma-factors, interacts with mitochondrial core polymerase Rpo41p	mitochondrion	Verified	Homodiploid	mtGM	UVerified	
YNL140C	YNL140C	1,462	0,010	2,965	0,049	-4,458	0,000	1,544	0,000	Dubious open reading frame unlikely to encode a protein, based on available experimental and comparative sequence data; partially overlaps the verified gene RLR1/YNL139C	unknown	Dubious	Homodiploid	CTF, ALF, CS	Doxorubicin, Metal	
YPL194W	DDC1	1,530	0,050	2,944	0,008	-2,307	0,011	3,184	0,000	DNA damage checkpoint protein, part of a PCNA-like complex required for DNA damage response, required for pachytene checkpoint to inhibit cell cycle in response to unrepaired recombination intermediates; potential Cdc28p substrate	nucleus	Verified	Homodiploid	BIM, CTF, CS, MR, MF	HU, MMS, γR, Camptothecin	
YNL001W	DOM34	0,422	0,003	2,899	0,034	1,717	0,000	-4,603	0,001	Endoribonuclease; functions in no-go mRNA decay, protein translation to promote G1 progression and differentiation, required for meiotic cell division; similar to the eukaryotic Pelota	cytoplasm	Verified	Homodiploid			SPO, ionic
YMR279C	YMR279C	-0,626	0,001	2,818	0,021	1,032	0,000	1,786	0,000	Putative protein of unknown function; identified as a heat-induced gene in a high-throughput screen; YMR279C is not an Essential gene	unknown	Uncharacterized	Homodiploid			

YER055C	***HIS1***	1,632	0,000	2,807	0,073	4,812	0,000	-3,677	0,000	ATP phosphoribosyltransferase, a hexameric enzyme, catalyzes the first step in histidine biosynthesis; mutations cause histidine auxotrophy and sensitivity to Cu, Co, and Ni salts; transcription is regulated by general amino acid control	intracellular	Verified	Homodiploid		Metal	
YLR037C	***DAN2***	1,995	0,042	2,789	0,091	-4,553	0,000	1,769	0,000	Cell wall mannoprotein with similarity to Tir1p, Tir2p, Tir3p, and Tir4p; expressed under anaerobic conditions, completely repressed during aerobic growth	cell wall	Verified	Homodiploid			
YER173W	***RAD24***	-0,292	0,049	2,779	0,017	1,003	0,001	-5,199	0,001	Checkpoint protein, involved in the activation of the DNA damage and meiotic pachytene checkpoints; subunit of a clamp loader that loads Rad17p-Mec3p-Ddc1p onto DNA; homolog of human and S. pombe Rad17 protein	nucleus	Verified	Homodiploid	BIM, ALF, GCR, CM, MF	HU, MMS, γR, UVerified, Camptothecin	
YKR010C	***TOF2***	0,294	0,000	2,766	0,019	1,137	0,001	-4,446	0,000	Protein required for rDNA silencing and nucleolar segregation during mitosis; stimulates Cdc14p phosphatase activity; identified as a topoisomerase I (Top1p) binding protein; displays synthetic genetic interactions with TOP1 and HPR1	nucleus, mitochondrion	Verified	Homodiploid			
YGR055W	***MUP1***	1,009	0,005	2,748	0,033	1,495	0,000	-1,707	0,000	High affinity methionine permease, integral membrane protein with 13 putative membrane-spanning regions; also involved in cysteine uptake	membrane	Verified	Homodiploid		HU	
YBR197C	***YBR197C***	0,999	0,003	2,742	0,100	1,432	0,000	-0,850	0,005	Putative protein of unknown function; green fluorescent protein (GFP)-fusion protein localizes to the cytoplasm and nucleus; YBR197C is not an Essential gene	nucleus, cytoplasm	Uncharacterized	Homodiploid			
YBR104W	***YMC2***	0,406	0,022	2,731	0,043	2,113	0,000	-2,132	0,000	Mitochondrial protein, putative inner membrane transporter with a role in oleate metabolism and glutamate biosynthesis; member of the mitochondrial carrier (MCF) family; has similarity with Ymc1p	mitochondrion	Verified	Homodiploid		MMS	
YGR161C	***RTS3***	-0,874	0,028	2,678	0,079	0,492	0,079	2,216	0,096	Putative component of the protein phosphatase type 2A complex	nucleus, cytoplasm	Uncharacterized	Homodiploid	RMCD		
YDR097C	***MSH6***	-0,918	0,000	2,651	0,007	2,545	0,000	-0,600	0,009	Protein required for mismatch repair in mitosis and meiosis, forms a complex with Msh2p to repair both single-base & insertion-deletion mispairs; potentially phosphorylated by Cdc28p	nucleus	Verified	Homodiploid	Can^R, MF		
YKR007W	***MEH1***	0,946	0,012	2,634	0,030	-5,167	0,000	2,207	0,000	Component of the EGO complex, which is involved in the regulation of microautophagy, and of the GSE complex, which is required for proper sorting of amino acid permease Gap1p; loss results in a defect in vacuolar acidification	vacuole, endosome	Verified	Homodiploid			
YLR109W	***AHP1***	0,067	0,045	2,632	0,036	-2,864	0,000	2,362	0,003	Thiol-specific peroxiredoxin, reduces hydroperoxides to protect against oxidative damage; function in vivo requires covalent conjugation to Urm1p	cytoplasm, plasma membrane	Verified	Homodiploid		OXY	
YOR037W	***CYC2***	0,499	0,045	2,628	0,076	2,217	0,000	-4,804	0,000	Mitochondrial peripheral inner membrane protein, contains a FAD cofactor in a domain exposed in the intermembrane space; exhibits redox activity in vitro; likely participates in ligation of heme to acytochromes c and c1 (Cyc1p and Cyt1p)	mitochondrion	Verified	Homodiploid			
YLR253W	***YLR253W***	-0,599	0,000	2,626	0,023	-4,072	0,000	1,818	0,002	Putative protein of unknown function; the authentic, non-tagged protein is detected in highly purified mitochondria in high-throughput studies; transcription is periodic during the metabolic cycle; YLR253W is not an Essential gene	mitochondrion	Uncharacterized	Homodiploid			
YPL013C	***MRPS16***	1,288	0,028	2,620	0,040	-4,584	0,000	1,199	0,001	Mitochondrial ribosomal protein of the small subunit	mitochondrion	Verified	Homodiploid		OXY	
YDR407C	***TRS120***	-0,785	0,004	2,566	0,009	2,982	0,000	-3,396	0,001	One of 10 subunits of the transport protein particle (TRAPP) complex of the cis-Golgi which mediates vesicle docking and fusion; involved in endoplasmic reticulum (ER) to Golgi membrane traffic	Golgi apparatus, Endoplasmic Reticulum	Verified	Essential			

YIR001C	SGN1	-0,875	0,008	2,563	0,004	1,057	0,001	-2,723	0,000	Cytoplasmic RNA-binding protein, contains an RNA recognition motif (RRM); may have a role in mRNA translation, as suggested by genetic interactions with genes encoding proteins involved in translational initiation	cytoplasm	Verified	Homodiploid	RMCD		CCP
YJR091C	JSN1	-1,487	0,000	2,515	0,072	1,783	0,000	-4,129	0,000	Member of the Puf family of RNA-binding proteins, interacts with mRNAs encoding membrane-associated proteins; overexpression suppresses a tub2-150 mutation and causes increased sensitivity to benomyl in wild-type cells	mitochondrion	Verified	Homodiploid			
YLR187W	SKG3	0,681	0,001	2,465	0,001	3,030	0,000	-1,035	0,001	Protein of unknown function; green fluorescent protein (GFP)-fusion protein localizes to the cell periphery, cytoplasm, bud, and bud neck; potential Cdc28p substrate; similar to Caf120p and Skg4p	bud, cytoplasm	Uncharacterized	Homodiploid			
YOR162C	YRR1	0,861	0,010	2,460	0,089	1,384	0,000	-4,589	0,002	Zn2-Cys6 zinc-finger transcription factor that activates genes involved in multidrug resistance; paralog of Yrm1p, acting on an overlapping set of target genes	nucleus	Verified	Homodiploid			
YIL001W	YIL001W	1,840	0,010	2,384	0,008	-4,450	0,000	2,468	0,000	Putative protein of unknown function; contains a BTB/POZ domain which generally function in protein interactions; deletion slightly improved competitive fitness in rich media; GFP-tagged protein is localized to the cytoplasm	cytoplasm	Uncharacterized	Homodiploid			
YOR304C-A	YOR304C-A	-0,560	0,000	2,316	0,049	1,503	0,000	-3,433	0,001	Protein of unknown function; green fluorescent protein (GFP)-fusion protein localizes to the cell periphery, cytoplasm, bud, and bud neck	bud, cytoplasm	Uncharacterized	Homodiploid			
YDR079W	PET100	0,548	0,026	2,316	0,021	3,042	0,000	-3,921	0,001	Chaperone that specifically facilitates the assembly of cytochrome c oxidase, integral to the mitochondrial inner membrane; interacts with a subcomplex of subunits VII, VIIa, and VIII (Cox7p, Cox9p, and Cox8p) but not with the holoenzyme	mitochondrion	Verified	Homodiploid	mtGM	OXY, Metal	
YMR175W	SIP18	1,322	0,001	2,303	0,044	-4,651	0,001	1,524	0,007	Protein of unknown function whose expression is induced by osmotic stress	soluble fraction	Verified	Homodiploid			
YML117W	NAB6	-0,930	0,001	2,265	0,010	-3,054	0,006	3,898	0,004	Putative RNA-binding protein, based on computational analysis of large-scale protein-protein interaction data	unknown	Verified	Homodiploid		γR, Doxorubicin	
YFL033C	RIM15	0,206	0,001	2,237	0,015	-4,726	0,000	2,444	0,000	Glucose-repressible protein kinase involved in signal transduction during cell proliferation in response to nutrients, specifically the establishment of stationary phase; identified as a regulator of IME2; substrate of Pho80p-Pho85p kinase	nucleus, cytoplasm	Verified	Homodiploid			SPO
YNL046W	YNL046W	-0,133	0,000	2,209	0,008	1,832	0,018	-4,019	0,000	Putative protein of unknown function; expression depends on Swi5p; GFP-fusion protein localizes to the endoplasmic reticulum; deletion confers sensitivity to 4-(N-(S-glutathionylacetyl)amino) phenylarsenoxide (GSAO)	Endoplasmic Reticulum	Uncharacterized	Homodiploid			
YBR300C	YBR300C	1,378	0,005	2,156	0,073	1,991	0,000	-1,029	0,010	Dubious open reading frame unlikely to encode a protein, based on available experimental and comparative sequence data; partially overlaps the verified gene YBR301W; YBR300C is not an Essential gene	unknown	Dubious	Homodiploid			
YBR244W	GPX2	-0,545	0,036	2,116	0,026	5,742	0,000	-4,306	0,000	Phospholipid hydroperoxide glutathione peroxidase induced by glucose starvation that protects cells from phospholipid hydroperoxides and nonphospholipid peroxides during oxidative stress	nucleus, cytoplasm	Verified	Homodiploid	RMCD	γR	
YKR075C	YKR075C	-0,349	0,004	2,115	0,020	-4,263	0,000	1,566	0,000	Protein of unknown function; similar to YOR062Cp and Reg1p; expression regulated by glucose and Rgt1p; GFP-fusion protein is induced in response to the DNA-damaging agent MMS	unknown	Uncharacterized	Homodiploid			
YDR193W	YDR193W	-0,261	0,000	2,071	0,046	1,560	0,000	-1,999	0,000	Dubious open reading frame unlikely to encode a functional protein, based on available experimental and comparative sequence data	unknown	Dubious	Homodiploid			
YLR312W-A	MRPL15	1,810	0,042	2,070	0,047	4,544	0,000	-0,548	0,008	Mitochondrial ribosomal protein of the large subunit	mitochondrion	Verified	Homodiploid			

YLL024C	***SSA2***	**0,842**	**0,001**	**2,047**	**0,046**	-4,631	0,000	**1,921**	0,000	ATP binding protein involved in protein folding and vacuolar import of proteins; member of heat shock protein 70 (HSP70) family; associated with the chaperonin-containing T-complex; present in the cytoplasm, vacuolar membrane and cell wall	cytoplasm, cell wall, vacuole, mitochondrion	Verified	Homodiploid			
YDL059C	***RAD59***	**-0,018**	**0,006**	**2,036**	**0,005**	1,440	0,000	-0,876	0,003	Protein involved in the repair of double-strand breaks in DNA during vegetative growth via recombination and single-strand annealing; anneals complementary single-stranded DNA; homologous to Rad52p	nucleus	Verified	Homodiploid	ALF, BIM, MR, MF	HU, MMS, Cisplatin, γR, Camptothecin, Doxorubicin, Tirapazamine	
YLR017W	***MEU1***	**0,655**	**0,001**	**2,014**	**0,036**	1,450	0,000	-2,010	0,004	Methylthioadenosine phosphorylase (mitochondrionAP), catalyzes the initial step in the methionine salvage pathway; affects polyamine biosynthesis through regulation of ornithine decarboxylase (Spe1p) activity; regulates ADH2 gene expression	cytoplasm	Verified	Homodiploid			
YKR049C	***FMP46***	**-1,325**	**0,001**	**2,014**	**0,080**	**1,532**	0,000	-1,372	0,000	Putative redox protein containing a thioredoxin fold; the authentic, non-tagged protein is detected in highly purified mitochondria in high-throughput studies	mitochondrion	Verified	Homodiploid			
YLL001W	***DNM1***	**-0,797**	**0,005**	**2,013**	**0,059**	**1,891**	0,000	-4,479	0,000	Dynamin-related GTPase required for mitochondrial fission and the maintenance of mitochondrial morphology, assembles on the cytoplasmic face of mitochondrial tubules at sites at which division will occur; also participates in endocytosis	mitochondrion, peroxisome	Verified	Homodiploid			

*this work

Instability phenotypes (the genomic screens data):

ALF – A-like faker [Yuen et al.]

BIM – diploid bimater [Yuen et al.]

CTF – chromosome transmission fidelity [Yuen et al.; Smith,S. et al.]

Can^R- canavanine resistance [Huang et al., Smith,S. et al]

GCR – gross chromosomal rearangements [Smith,S. et al.; Myung, K. 2001; Myung, K. 2003]

HAP – sensitivity to the mutagenic and toxic action of base analogs 6-hydroxylaminopurine [Stepchenkova,E.I. et al.]

LOH – loss of heterozygosity [Andersen,M.P. et al.]

RMCD - - restore mating competence to diploid [Kaeberlein,M et al.]

Instability phenotypes (the SGD data):

CPM - chromosome maintenance decreased

CS – colony sectoring

CSA – chromosome segregation abnormal

MF – mutation frequency increased

MR - mitotic recombination increased

mtGM - mitochondrial genome maintenance

rho-MF – rho- mutation frequency

TL – telomere length decreased

TE-T - transposable element transposition increased

Genotoxic stress sensitivity (SGD data):

Bleomycin A2 – resistance to bleomycin A2 decreased

Camptothecin – resistance to camptothecin decreased

Cisplatin – resistance to cisplatin decreased

Doxorubicin - resistance to doxorubicin decreased

γR - gamma radiation resistance decreased

HU – resistance to hydroxyurea decreased

Metal – metal resistance decreased

MMS – resistance to methyl methanesulfonate decreased

OXY – oxidative stress resistance decreased

Phleomycin – resistance to phleomycin decreased

Tirapazamine - resistance to tirapazamine decreased

UV - UV resistance decreased

Other genome related phenotypes

Benomyl- gamma radiation resistance decreased

CCP – cell cycle progression

ionic – ionic stress resistance decreased

NT – nuclear transport

SA – silencing abnormal

SPO – sporulation efficiency decreased

Andersen,M.P., Nelson,Z.W., Hetrick,E.D. and Gottschling,D.E. (2008) A Genetic screen for increased loss of heterozygosity in *Saccharomyces cerevisiae*. Genetics, 179, 1179–1195.

Huang,M.E., Rio,A.G., Nicolas,A. and Kolodner,R.D. (2003) A genomewide screen in Saccharomyces cerevisiae for genes that suppress the accumulation of mutations. Proc. Natl. Acad. Sci. USA, 100, 11529–11534.

Kaeberlein,M., Kudlow,B.A., MacKay,V., Lockshon,D. and Kennedy,B.K. (2008) Single-gene deletions that restore mating competence to diploid yeast. FEMS Yeast Res., 8(2), 276–286.]

Myung,K., Datta,A., and Kolodner,R.D. (2001) Suppression of Spontaneous Chromosomal Rearrangements by S Phase Checkpoint Functions in *Saccharomyces cerevisiae.* Cell, 104, 397–408.

Myung,K., Pennaneach,V., Kats,E.S. and Kolodner,R.D. (2003) Saccharomyces cerevisiae chromatin-assembly factors that act during DNA replication function in the maintenance of genome stability. PNAS, 100, 6640–6645.

Smith,S., Hwang,J.Y., Banerjee,S., Majeed,A., Gupta,A., and Myung,K. (2004) Mutator genes for suppression of gross chromosomal rearrangements identified by a genome-wide screening in *Saccharomyces cerevisiae*. Proc. Natl. Acad. Sci. USA, 101, 9039-9044.

Stepchenkova,E.I., Kozmin,S.G, Alenin,V.V., and Pavlov,Y.I (2005) Genome-wide screening for genes whose deletions confer sensitivity to mutagenic purine base analogs in yeast. BMC Genetics, 6, 31

Yuen,K.W.Y., Warren,C.D., Chen,O., Kwok,T., Hieter,P. and Spencer,F.A. (2007) Systematic genome instability screens in yeast and their potential relevance to cancer. Proc. Natl. Acad. Sci. USA, 104, 3925–3930.
