# Supplementary material for: Dipoid-Specific Genome Stability Genes of S. cerevisiae: Genomic Screen Reveals Haploidization as an Escape from Persisting DNA Rearrangement Stress
Source: PLoS One. 2011 Jun 17;6(6):e21124. doi: 10.1371/journal.pone.0021124 (PMC3117874; doi:10.1371/journal.pone.0021124)
Supplement: Table S3 — Correlation between the results of large scale SLM screens and semi-quantitative individual SLM assay. (PDF) [file pone.0021124.s011.pdf]

Table S3. Correlation between the results of large scale SLM screens and semi-quantitative SLM assay performed individually on selected deletion clones.

| | CAN selection | | FOA selection | |
|---|---|---|---|---|
| Strains, for which the phenotype determined by large scale microarray screen was confirmed by individual tests | 39[a] | 76.5% | 36[f] | 76.6% |
| Strains that gave a positive signal in large scale microarray screen, which in individual tests turned out to be the result of the resistant phenotype of respiratory incompetent cells appearing in the backgrounds of these particular deletions | 2[b] | 3.9% | 2[g] | 4.3% |
| Strains showing mutator phenotype in individual tests, hence expected to give a positive signal in large scale microarray screen, but probably too sensitive to the selection conditions used and therefore eliminated from the population | 6[c] | 11.8% | 1[h] | 2.1% |
| Strains that gave a positive signal in large scale microarray screen, but unreliable due to severe underrepresentation in analyzed population, that was not confirmed by the individual tests | 3[d] | 5.9% | 6[i] | 12.7% |
| True false positives: strains that gave reliable positive signals in large scale microarray screen that was not confirmed by the individual tests | 1[e] | 2.0% | 2[j] | 4.3% |
| All strains examined in individual tests | 51 | 100.0% | 47 | 100.0% |
| Strains showing positive correlation between large scale microarray screen and data obtained in all individual tests | 41 | 80.4% | 38 | 80.9% |

[a] *COX14, CPA1, CTF18, CTF8, DAP2, DBP6, DPH1, DSK2, FAP1, LPE10, MLH2, MSS116, MTO1, PEX19, PHM6, POL31, PRX1, ROX1, SAE2, SEN2, SLG1, SMM1, SPO11, TRP5, UFD1, VPS33, XRS2, YBL059W, YBR099C, YBR144C, YDR278C, YER068C-A, YIL039W, YLR111W, YML084W, YMR111C, YNR068C, RTR1, YPL183W-A*

[b] *ISW2, YLL017W*

[c] *LSM2, MLH1, PRE5, SHU1, SUR1, YKU70*

[d] *PHO23, SWD3, UBC12*

[e] *ARP1*

[f] *ARG2, BDF1, BUD23, CHS5, COX11, FAD1, FKS1, FPS1, FYV12, GET2, HAP2, IRA2, LPE10, MMS22, MRS1, MTO1, OCA1, PAT1, PDR17, PDX3, PUP3, RAD6, RAS2, REF2, STB5, SWI6, THP1, UBC12, UFD1, YBR194W, YCL045C, YDL118W, YGR064W, YGR150C, YGR190C, YMR111C*

[g] *NIP1, YIL057C*

[h] *YJR018W*

[i] *CBS2, CCR4, RPN2, MPP10, TAF3, YDR034W-B*

[j] *MOT2, VAN1*
