# Supplementary material for: Dipoid-Specific Genome Stability Genes of S. cerevisiae: Genomic Screen Reveals Haploidization as an Escape from Persisting DNA Rearrangement Stress
Source: PLoS One. 2011 Jun 17;6(6):e21124. doi: 10.1371/journal.pone.0021124 (PMC3117874; doi:10.1371/journal.pone.0021124)
Supplement: Table S4 — Subcellular localization of 249 ORFs selected in our genome-wide SLM screens. (PDF) [file pone.0021124.s012.pdf]

Table S4. Subcellular localization of 249 ORFs selected in our genome-wide SLM screens.

Subcellular localization	Number of ORFs	% of ORFs
nucleus	80	32.12
unknown	43	17.27
mitochondrion	39	15.67
cytoplasm	34	13.67
Endoplasmic Reticulum	19	7.63
bud	8	3.21
Golgi Apparatus	5	2.01
vacuole	5	2.01
peroxisome	4	1.61
membrane	3	1.20
actin patch	2	0.80
Spindle Pole Body	2	0.80
vesicles	2	0.80
wall	2	0.80
extracellular region	1	0.40
