# Supplementary material for: Dipoid-Specific Genome Stability Genes of S. cerevisiae: Genomic Screen Reveals Haploidization as an Escape from Persisting DNA Rearrangement Stress
Source: PLoS One. 2011 Jun 17;6(6):e21124. doi: 10.1371/journal.pone.0021124 (PMC3117874; doi:10.1371/journal.pone.0021124)
Supplement: Materials and Methods S1 — (DOC) [file pone.0021124.s013.doc]

**Supplementary Materials and Methods**

*Strains and plasmids construction*

*S. cerevisiae* gene knock-out collections version 2, created by *Saccharomyces* Genome Deletion Project (http://www-sequence.stanford.edu/group/yeast_deletion_project/) were obtained from Open Biosystems (Huntsville, USA) as deep-frozen glycerol stocks in 96 well microtiter plates.

Haploid BY4741 strain (*MAT*a *his3Δ leu2Δ ura3Δ met15Δ*), haploid BY4742 strain (*MAT*α *his3Δ leu2Δ ura3Δ lys2Δ*) and diploid BY4743 strain *(MATa/alpha his3Δ/his3Δ leu2Δ/leu2Δ ura3Δ/ura3Δ LYS2/lys2Δ MET15/met15Δ*) and their derivatives were used, unless stated otherwise. HB1-4Da (*MATa thr1-1*) and HB2-1Aα (*MATα thr1-1*) strains were used for LOF mutagenesis screen at the mating type locus.

A DNA fragment carrying *can1::LEU2* was obtained by amplifying *LEU2* gene from pRS315 plasmid with primers Can1K.up: 5'-CTTCAGACTTCTTAACTCCTGTAAAAACAAAAAAAA
AAAAAGGCATAGCATCGGTGATGACGGTGAAAAC-3' and Can1K.lw: 5'- AGAATGCGA
AATGGCGTGGAAATGTGATCAAAGGTAATAAAACGTCATATAAAAACTTGATTAGGG
TGAT-3', both having segments of 20 bp from the *LEU2* selection marker and 50 bp fragments from upstream or downstream of the *CAN1* target gene. We introduced this fragment into the BY4741 strain. The resulting strain YMR2 was the source of genomic DNA used as a template in PCR reaction with primers: Can1.up 5'-AGAGCTCGATACGAGATAAAGCACAAAT-3' and Can1.lw 5'-GGCTGCAGTGAAGATAACGAAAAATGAGT-3'. The resulting 3.28 kb PCR fragment containing *CAN1* gene with 255 bp upstream and 300 bp downstream flanks was digested with PstI, SacI and ligated into pUC19 vector digested with the same enzymes. Plasmid pUC-can1::LEU2 was used for subsequent PCR reactions as template for preparing a disruption cassette for synchronous disruption transformations.

To prepare the 1.7kb *URA3* cassette for reconstitution of *URA3* locus in BY474X strain we have used SP4 (*MAT*α *arg4 leu1*) strain genomic DNA as a template and the pair of primers:
Ura3.up 5'-AATCTAGACGATGATAACAAACCGAAGT-3'
and Ura3.lw 5'-GGATCCGGCTACACCAGAGATACATA-3'.

To construct heterodiploid *CAN1/can1::LEU2* or *URA3/ura3Δ* strains for individual tests of LOF mutagenesis, we have used strains from the YKO diploid collection or wild-type BY4743 strain as parental strains and transformed them with *can1::LEU2* or *URA3* cassettes, respectively.

Diploid strains 2n ctf18, 2n ndt80 and 2n ndt80 ctf18, homozygous for *ctf18Δ* and *ndt80Δ* gene deletions were prepared as follows:

To create *ctf18Δ* deletion strains, DNA fragment bearing *ctf18::kanMX4* was amplified with primers: ctf18.up 5' TTCCTTTTCTTGTTGCTATT 3' and ctf18.lw 5' GCTCTTCGGGGTTCCATTTA 3' using genomic DNA from BY4741 *ctf18Δ* strain as template. The resulting PCR product was used to replace *CTF18* with *ctf18::kanMX4* allele in two haploid strains of opposite mating types: 1n (BY4741 *URA3*) and α c:L (BY4742 *can1::LEU2*).

To make *ndt80Δ* deletion strains *ndt80::HIS3* deletion cassette was prepared by PCR, using the pRS303 vector as a source of *HIS3* gene and the pair of hybrid primers carrying 20 nt sequences of *HIS3* gene flanks and 50 nt sequences of upstream and downstream regions of the *NDT80* gene: ndt80_k.up: 5' TACTAACCTTTCATTAAAGAGAAATAACAATATTATAAAAAGCGCTTA
AATCGGTGATGACGGTGAAAAC 3' and ndt80_k.lw: 5' GATCCACAGAATTCGCATAT
TTTTTTAACGATTTAAAATCATTAGTTTATAAAAACTTGATTAGGGTGAT 3'. The resulting PCR product was used to replace *NDT80* gene in the same haploid strains as described above, namely: 1n (BY4741 *URA3*) and α c:L (BY4742 *can1::LEU2*). Primers: ndt80.up:
5' AAAACAAGGCACAACAATGA 3', ndt80.lw: 5' TGAAGGGAACTGATAACAAA 3' and his3_c.up: 5' ATGGTACCAACGATGTTC 3' were used for checking correctness of disruption cassette integration into *NDT80* gene locus.

To make *ndt80Δ ctf18Δ* double deletion strains *ctf18::kanMX4* cassette was introduced into haploids with opposite mating types, 1n ndt80 and α c:L ndt80 strains.

Respective haploids of opposite mating types were crossed to give 2n ctf18 diploid (BY4743 *CAN1/can1::LEU2 URA3/ura3Δ ctf18::kanMX4/ctf18::kanMX4*), 2n ndt80 diploid (BY4743 *CAN1/can1::LEU2 URA3/ura3Δ ndt80::HIS3/ndt80::HIS3*) and 2n ndt80 ctf18 double deletion diploid (BY4743 *CAN1/can1::LEU2 URA3/ura3Δ ndt80::HIS3/ndt80::HIS3 ctf18::kanMX4/ctf18::kanMX4*). Wild-type reference diploid strain was obtained by crossing 1n with α c:L parental haploids.

Diploid 2n ctf8, 2n mto1, 2n phm6 and 2n ted1 strains homozygous for *ctf8Δ*, *mto1Δ*, *phm6Δ* and *ted1Δ* respectively were prepared in a similar way using the following primer pairs:
ctf8.up: 5' TGAACAAGATTAGCGAGCCTA 3', ctf8.lw: 5' TGAAAACGAACAACTGGGGT 3', mto1.up: 5' CGGGGGTCTTCTACTTTTGT 3', mto1.lw: 5' GGCGTTCTTGTTCCTCTTCC 3', phm6.up: 5' ATTTGGATGTTCTGCCCTAT 3', phm6.lw: 5' TTTTCTTGACTGCGGTGTTC 3', ted1.up: 5' CGCAGATGAAAATGGAACAG 3', ted1.lw: 5' AGAACAACAAAAACAAGAAA 3', and genomic DNA prepared from the appropriate deletion strains of the haploid YKO collection.

Zygotes were isolated with the aid of micromanipulator (Singer Instruments, UK).

*Culture media and conditions*

Rich YPD medium contained 1% yeast extract (Difco), 2% bactopeptone (Difco), 2% glucose (POCh). YPD-GPS was YPD medium supplemented with G-418 (100 μg per ml), penicillin (50 μg per ml) and streptomycin (50 μg per ml). Solid YPD-GPS also contained 2% agar (Difco). Synthetic complete (SC) medium contained 0.67% Yeast Nitrogen Base (Difco), 2% glucose, supplemented with all amino acids, uracil and adenine. Solid SC also contained 2% agar.

SC medium without leucine or uracil was used for selecting transformants. Solid SC medium without arginine, supplemented with 30 μg per ml of canavanine and solid SC medium supplemented with 1 mg per ml of 5’-fluoroorotic acid (5’-FOA) were used for selection of mutant colonies.

Unless stated otherwise, liquid cultures were grown at 28°C with agitation at ~ 200 RPM. Growth on solid media was at 28°C.

*Preparation of Yeast Knockout (YKO) diploid pool*

YKO homodiploid (HD) collection and YKO essential gene heterodiploid (ESS) collection were inoculated into 96 well plates filled with ~150 µl of liquid YPD-GPS using a 96 pin replicator. After 2 days of growth without agitation culture plates were examined visually for cell density in each well, and the lower density cultures due to slow growth of some deletion strains were concentrated by removing 70 to 120 µl of medium from above the cell sediment. Liquid cultures were then copied with a 96-pin replicator onto Omnitray plates (Nunc) filled with solid YPD-GPS, while ensuring that every yeast strain transferred well. Plates containing a high proportion of slow growers were replicated in duplicate to increase their representation and to balance the pool with respect to every strain in the collection. Plates were incubated at 28°C for 3 days. Yeast colonies were then washed off the plates with liquid YPD-GPS, the density of the cell suspension was adjusted to 1 x 10^9^ cells per ml, and glycerol was added to final concentration of 15%. The cell suspension was aliquoted into cryogenic vials (~10^9^ cells per vial), flash frozen in liquid nitrogen and kept in –80°C freezer.

***Preparation of derivative YKO diploid pools heterozygous with respect to mutagenesis marker, suitable for SLM assay***

*Preparation of CAN1/can1::LEU2 heterodiploid pool suitable for LOH mutagenesis assay by synchronous disruption of single CAN1 marker in the complete YKO diploid pool.*

The transformation procedure was adapted from Pan *et al.* [1]. We optimized the ratio of the amount of transforming DNA to the number of cells used to reach an acceptable yield of transformants while keeping the proportion of clones with the disruption of both copies of *CAN1* gene negligible. 10^9^ cells of YKO diploid mutant pool were inoculated into 1 liter of liquid YPD-GPS medium. Cells were grown at 28°C with agitation until they reached a density of 2-3 x 10^7^ cells per ml. Cells were centrifuged at 1500-2000 RPM for 5 min, washed once with water and once with 0.1 M LiOAc. The cell pellet was resuspended in 0.1 M LiOAc at 3.3 x 10^9^ cells per ml. The following were added to a sterile Falcon tube: 250 µl of Herring Sperm DNA (10 mg per ml, freshly boiled at 100°C for 5 min and cooled on ice), 25-50 µg of PCR amplified disruption cassette as transforming DNA, 3.75 ml of competent cells (3.3 x 10^9^ cells per ml), 10 ml 50% PEG3350, 1.1 ml 1 M LiOAc. The contents were mixed thoroughly but carefully and incubated at 28°C for 30 min with occasional stirring. 1.68 ml of DMSO was then added to 10% final concentration and the cells were heat-shocked at 42°C for 13 min. Cells were centrifuged at 3000 RPM for 1 min, washed once in water and resuspended in 25 ml of 5 mM CaCl_2_. After 5 min incubation in this solution at room temperature, the cells were immediately plated in 500 μl aliquots onto fifty 150 mm diameter plates containing SC medium without leucine. This step was done quickly enough to avoid keeping cells longer than 15 min in CaCl_2_ solution. To estimate transformation efficiency 100x diluted cell suspension was plated. Plates were incubated at 28°C for three days. Using this protocol, at least 3 x 10^5^ transformants were obtained, which is equivalent to >50 fold coverage of the deletion clone collection. Transformed cells were then washed from the plates with a glass spreader and ~5 ml of liquid YPD per plate, followed by an additional 3 ml YPD wash. Combined cell suspensions were mixed well and adjusted to 1x10^9^ cells per ml with YPD. Glycerol was added to the final concentration of 15%, cell suspension was aliquoted into cryogenic vials (~10^9^ cells per vial), flash frozen in liquid nitrogen and kept in –80°C freezer.

*Preparation of URA3/ura3Δ heterodiploid pool suitable for SLM assay by synchronic reconstitution of single wild-type URA3 marker in the whole YKO diploid pool.*

The procedure was essentially the same except that PCR amplified genomic fragment containing *URA3* gene was used as transforming DNA to replace one of the *ura3Δ* loci with the wild-type *URA3*, and the selection used SC without uracil.

To verify that the derivative pools prepared this way are still sufficiently representative, the clone representation in the pools before and after transformation was compared using the same method of labeling and hybridization to the barcode microarray, as used for spontaneous mutagenesis screening.

*Genomic DNA Isolation*

10^9^ cells (2 x 10^5^ fold coverage of yeast genome) were thawed, resuspended in 0.5 ml of 1 M Sorbitol/0.1 M EDTA, and 20 μl of 2.5 mg per ml zymolyase 100T solution was added. The suspension was mixed well and incubated 1 h at 37°C with occasional mixing by inverting the tube. Spheroplasted cells were spun down in microcentrifuge at top speed for 1 min, resuspended in 0.5 ml 10 mM Tris, 50 mM EDTA buffer and 50 µl of 10% SDS was added to the suspension. Tube content was mixed very delicately by inverting, and the tube was incubated for 1 h at 65°C. After adding 200 µl of 5 M potassium acetate the tube was mixed vigorously and immediately put on ice for 1 h. The tube was spun in the microfuge at top speed at 4°C for 15 min, and the supernatant (~ 750 µl) was transfered to a new tube. Genomic DNA was precipitated by adding an equal volume (~750 µl) of isopropanol and gentle mixing by inverting the tube. The precipitate was collected by centrifugation at top speed at 4°C for 5 min, DNA pellet was washed with 80% EtOH and dried by leaving the tube open on the bench at RT for 5-10 min. Dried DNA sample was dissolved in 400 µl of 10 mM Tris-HCl buffer pH 8.0. To remove the RNA from the DNA solution 5 µl of 10 mg per ml of RNase A (Sigma) was added and the mixture was incubated at 37°C for 30 min. DNA sample was then extracted with 400 µl of phenol pH 8.0. The aqueous phase was transferred to a Phase Lock Gel tube (Eppendorf) and centrifuged in the microfuge at top speed for 10 min to remove phenol residues. The aqueous phase was transferred to a new tube and the DNA was precipitated by the addition of 40 µl of 3 M sodium acetate pH 5.4 and 1 ml of 100% EtOH, followed by gentle mixing by inverting the tube. After several min at room temperature the DNA precipitate was collected in the microfuge at top speed at 4°C for 10 min, washed with 80% EtOH, air dried and resuspended in 100 µl of 10mM Tris-HCl buffer pH 8.0. The concentration of genomic DNA was determined using NanoDrop spectrophotometer. The quality of the DNA preparation was estimated by separation of 2‑3 µl of the solution on 1% agarose gel. The entire genomic DNA preparation formed a discrete band with an apparent size of >20 kb. For labeling procedure the genomic DNA was diluted to 100ng/μl.

*Microarrays*

To design our barcode microarrays we used e-Array on-line tool from Agilent, Santa Clara, USA, (Agilent Technologies eArray**,** http://earray.chem.agilent.com/earray). We chose 4x44k format. The barcode oligo sequences were made exactly as in most recent Yeast Knock-Out collection version 2 of Saccharomyces Genome Deletion Project (SGDP). Single set of 44k probes contained all barcodes in triplicate, split into separate subsets of UPTAGs and DOWNTAGs and randomized within subsets. Some initial experiments were done with a different microarray design, namely, in 1x22k format custom made by Agilent (GEO Accession viewer, http://www.ncbi.nlm.nih.gov/geo/query/acc.cgi?acc=GPL1444).

*DNA labeling and hybridization to Agilent Barcode Arrays*

*Sequences of primers used for labeling blocking and hybridization:*

D1 – CGGTGTCGGTCTCGTAG

D2 – ATCGATGAATTCGAGCTCG

U1 – GATGTCCACGAGGTCTCT

U2 – CGTACGCTGCAGGTCGAC

Cy3-U3 – 5’-Cy3-GTCGACCTGCAGCGTACG

Cy5-U3 – 5’-Cy5-GTCGACCTGCAGCGTACG

Cy3-D3 – 5’-Cy3-CGAGCTCGAATTCATCGAT

Cy5-D3 – 5’-Cy5-CGAGCTCGAATTCATCGAT

Cy3-G – 5’-Cy3-GGATACACTGACCAGCTACGATGAT

Cy5-G – 5’-Cy5-GGATACACTGACCAGCTACGATGAT

*PCR labeling*

Each PCR reaction for UPTAG or DNTAG labeling contained in a final volume of 100 μl: 2.5 U of BIOTOOL polymerase (BIOMOL), 10 μl 10x Reaction Buffer, 10 μl 2 mM dNTP mix, 150 ng genomic DNA and 10 μl of 5 µM primer mix: one of U1+Cy3-U3, U1+Cy5-U3, D1+Cy3-D3, or D1+Cy5-D3. Each reaction mix was split into two 50 μl aliquots kept in separate tubes. The reaction was started with 3 min of melting at 94°C followed by 30 cycles of PCR reaction (94°C 15 sec - 50°C 15 sec - 72°C 30 sec) and 3 min incubation at 72°C. Subsequently the aliquots of reaction mixes were recombined and the labeled probes were stored frozen in the dark until use.

*Microarray hybrydization*

Four PCR labeling reactions were used for a single hybridization: UPTAGs and DNTAGs from the first DNA sample labeled with Cy3 and UPTAGs and DNTAGs from the second DNA sample labeled with Cy5. 50 μl each of the two UPTAG reactions were mixed with 40 μl of 250 μM U1 and 40 μl of 250 μM U2 primers to block the common primer sequences on the labeled strand. Likewise, 50 μl each of the two DNTAG reactions were mixed with 40 μl of 250 μM D1 and 40 μl of 250 μM D2 primers. Both mixes were incubated for 2-3 min at 95-100°C, then put on ice for 2 min. The mixes were then combined, 36 μl 3 M sodium acetate pH 5.4 and 840 μl 95% EtOH were added and after mixing well the probe was put into liquid nitrogen bath for 2 min to precipitate the DNA. The probe was collected by a 15 min centrifugation at top speed in a microcentrifuge. A small violet precipitate was visible at this point. The pellet was washed with 80% EtOH, air dried and dissolved in 7.5 μl of water. Dissolved probe was incubated for 5 min in 75°C and added to hybridization solution containing 110 μl 1 x SSTE (1 M NaCl, 10 mM Tris, pH 7.5, 0.5% Triton X-100), 1 μl each of 5 nM Cy3-G and Cy5-G gridline primers and 0.22 μl 0.5 M DTT prewarmed to 42°C. The hybridization probe was mixed gently to avoid generating foam and kept at 42°C until the microarray slide was ready. Gasket slide (Agilent) was put into the hybridization chamber (Agilent), probes in hybridization solution were pipetted onto each well of the gasket and covered with microarray slide (oligo probes down). The hybridization chamber was closed, rotated slowly to moisten the microarray slide with the hybridization mix without producing excessive air bubbles and put into the hybridization oven for 16 h, 42°C, 10 RPM.

*Microarray washing*

Washing was done in Falcon tubes in three washing buffers based on 20x SSPE (3 M NaCl, 200 mM NaH_2_PO_4_, 50 mM EDTA pH 7.4). Hybridization chamber was opened, microarray-gasket sandwich was dropped into the first wash solution (6x SSPE + 0.05% Triton X100) and separated. The gasket slide was removed and the microarray slide was washed by rocking the Falcon tube several times. The slide was transferred into the second wash solution (1x SSPE) and washing was repeated. The final wash was done in 0.06x SSPE. The slide was taken out from the final wash solution with a slow steady move to allow its hydrophobic surface to repel the solution without leaving droplets.

*Scanning and feature extraction*

Immediately after washing, the slides were scanned using Axon GenePix 4000B scanner (Molecular Devices, USA) at 5 μm and 100% laser power. PMT gain was adjusted individually (between 600 and 800 volts) to obtain optimal images. Feature extraction was done using GenePix Pro 3.0 or GenePix Pro 6.1 (Molecular Devices, USA).

For each biological replicate, two independent labeling reactions with dye swapping were prepared, and two hybridizations were done.

**References:**

1. Pan X, Yuan DS, Ooi SL, Wang X, Sookhai-Mahadeo S, et al. (2007) dSLAM analysis of genome-wide genetic interactions in *Saccharomyces cerevisiae*. Methods 41: 206-221.
